# Preparation of Pyridylamido Hafnium Complexes for Coordinative Chain Transfer Polymerization

**DOI:** 10.3390/polym12051100

**Published:** 2020-05-11

**Authors:** Kyung Lee Park, Jun Won Baek, Seung Hyun Moon, Sung Moon Bae, Jong Chul Lee, Junseong Lee, Myong Sun Jeong, Bun Yeoul Lee

**Affiliations:** 1 Department of Molecular Science and Technology, Ajou University, Suwon 443-749, Korea; rudfl93@ajou.ac.kr (K.L.P.); btw91@ajou.ac.kr (J.W.B.); freethemoon@ajou.ac.kr (S.H.M.); bsm1029@ajou.ac.kr (S.M.B.); leejc@ajou.ac.kr (J.C.L.); 2 Department of Chemistry, Chonnam National University, Gwangju 500-757, Korea; leespy@chonnam.ac.kr; 3 Intellectual Property Education Center, Anyang University, Anyang 708-113, Korea; jmsun99@daum.net

**Keywords:** polyolefin, pyridylamido hafnium complex, coordinative chain transfer polymerization, dialkylzinc, post-metallocene

## Abstract

The pyridylamido hafnium complex (**I**) discovered at Dow is a flagship catalyst among postmetallocenes, which are used in the polyolefin industry for PO-chain growth from a chain transfer agent, dialkylzinc. In the present work, with the aim to block a possible deactivation process in prototype compound **I**, the corresponding derivatives were prepared. A series of pyridylamido Hf complexes were prepared by replacing the 2,6-diisopropylphenylamido part in **I** with various 2,6-R_2_C_6_H_3_N-moieties (R = cycloheptyl, cyclohexyl, cyclopentyl, 3-pentyl, ethyl, or Ph) or by replacing 2-iPrC_6_H_4_C(H)- in **I** with the simple PhC(H)-moiety. The isopropyl substituent in the 2-iPrC_6_H_4_C(H)-moiety influences not only the geometry of the structures (revealed by X-ray crystallography), but also catalytic performance. In the complexes bearing the 2-iPrC_6_H_4_C(H)-moiety, the chelation framework forms a plane; however, this framework is distorted in the complexes containing the PhC(H)-moiety. The ability to incorporate α-olefin decreased upon replacing 2-iPrC_6_H_4_C(H)-with the PhC(H)-moiety. The complexes carrying the 2,6-di(cycloheptyl)phenylamido or 2,6-di(cyclohexyl)phenylamido moiety (replacing the 2,6-diisopropylphenylamido part in **I**) showed somewhat higher activity with greater longevity than did prototype catalyst **I**.

## 1. Introduction

**Scheme 1:**
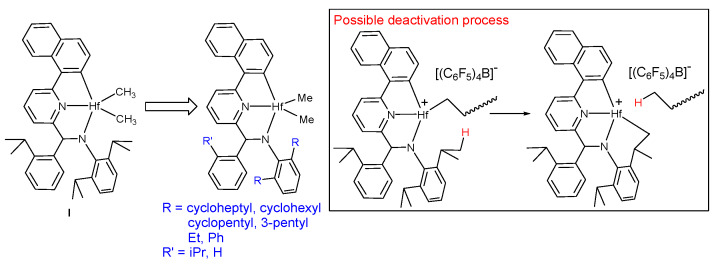
The flagship catalyst used in coordinative chain transfer copolymerization, a possible process of its deactivation, and the desired complexes that were an aim of this work.

Homogeneous single-site catalysts, which were introduced by Kaminsky with the serendipitous discovery of methylaluminoxane, have evolved from the original metallocenes (constructed via two cyclopentadienyl ligands) to half-metallocenes (constructed using a cyclopentadienyl ligand) and further to postmetallocenes (constructed via noncyclopentadienyl ligands). A representative flagship catalyst among postmetallocenes is a pyridylamido hafnium complex (**I** in Scheme 1) discovered by high-throughput screening in the early 2000s [1,2,3,4]. It shows advantageous performance in ethylene and α-olefin (co)polymerization reactions, though its activation reaction is rather tricky [5,6,7]. In ethylene/α-olefin copolymerization reactions, it is capable of incorporating a large amount of α-olefins (uniquely among Hf catalysts) [8,9]. α-Olefin content in the copolymer generated with I is comparable with that in the copolymer generated with a constrained geometry complex, [Me_2_Si(η^5^-C_5_Me_4_)N*t*Bu]TiMe_2_, though its activity is significantly lower than that of the constrained geometry complex (~^1^/_15_). Besides this, it can polymerize a propylene monomer with high isoselectivity [10,11,12]. The most surprising feature is that the β-elimination process (or a β-hydrogen transfer reaction) is completely prevented with **I**, enabling living olefin polymerization and consequently enabling the architecture of high-molecular-weight polyolefin chains of various block compositions [13]. Density functional theory calculations have shown that an agostic interaction between a Hf center and a β-hydrogen, via which the β-elimination process takes place, is absent in the activated complex of **I** [12]. Such a β-elimination process is inevitable especially in ethylene/α-olefin copolymerization reactions performed with the conventional Zr-based metallocene and Ti-based half-metallocene catalysts [14].

In living olefin polymerization, only one polymer chain is limitedly grown per a molecule of catalyst. A practical and commercially relevant method—coordinative chain transfer polymerization (CCTP)—has been developed, in which chain transfer agents (e.g., Et_2_Zn) are deliberately added in excess relative to **I** (e.g., [Zn]/[Hf] > 100) [15,16,17]. A rapid alkyl exchange between the chain-growing Hf center and chain transfer agent Zn sites results in transformation of the fed Et_2_Zn to (polyolefinyl)_2_Zn. In CCTP performed with **I**, PO chains are generated as a form of (polyolefinyl)_2_Zn with a rather narrow molecular-weight distribution (*M*_w_/*M*_n_ ≈ 1.7) and with negligible formation of PO chains not attached to Zn sites, owing to a feature of I ensuring the rapid alkyl exchange process and the absence of the β-elimination process [16,18]. Due to these characteristics, it is possible to grow PO chains with variation of ethylene/α-olefin composition, either by sequential variation of the ethylene/α-olefin feed ratio or by means of a dual catalytic system with distinctly different monomer reactivity, enabling commercial production of olefin block copolymers composed of hard crystalline and soft rubbery PO blocks [19,20,21,22,23]. A method has also been developed to grow polystyrene (PS) chains further from the CCTP product (polyolefinyl)_2_Zn, thereby allowing for the synthesis of commercially relevant PO-*block*-PS and PS-*block*-PO-*block*-PS via one-pot synthesis [14,24,25,26,27].

A drawback of **I** is that the activated complex of **I** had short lifetime (became completely deactivated within ~40 min), when the CCTP was performed at a typical polymerization temperature of ~100 °C. A possible deactivation process was hypothesized: σ-bond metathesis between CH(Me)CH_2_−H and Hf−C bonds thus forming a 6-membered metallacycle with liberation of a polymer chain (Scheme 1) [28]. With the expectation to find a long-lived catalyst through blockage of such a deactivation process, a series of derivatives was prepared in this work by replacement of the 2,6-diisopropylphenylamido part in **I** with various arylamido 2,6-R_2_C_6_H_3_N-moieties (R = cycloheptyl, cyclohexyl, cyclopentyl, 3-pentyl, ethyl, or Ph). Previously, it has been observed that such a σ-bond metathesis reaction can be blocked by replacing the 2,6-diisopropylphenylamido part with a 2,6-diethylphenylamido moiety in related pincer Hf complexes [29]. In other catalysis [performed with Pd complexes constructed via N-heterocyclic carbene (NHC) ligands], the influence of substituents at the 2,6-position in aryl-N moieties is substantial, and derivatization of the NHC ligand has been carried out by replacing common 2,6-diisopropylphenyl-N parts with 2,6-di(3-pentyl)phenyl-N and 2,6-di(3-heptyl)phenyl-N moieties [30,31]. Much research has also been performed on **I** [32,33,34,35,36,37,38], and the synthesis of its analogs with the aim to improve the catalytic performance deserves attention [7,29,39,40,41,42]. Subtle change in the ligand framework sometimes results in dramatic improvement in the polymerization performance and, hence, modification of the substituents has been a main research theme in the development of postmetallocecenes [43,44,45,46,47,48,49].

## 2. Materials and Methods

All manipulations were performed in an inert atmosphere in a standard glove box and by Schlenk techniques. Toluene, hexane, and tetrahydrofuran (THF) were distilled from benzophenone ketyl. Methylcyclohexane (anhydrous grade) utilized for the polymerization reactions was purchased from Tokyo Chemical Industry and was purified over a Na/K alloy. Sublimed-grade HfCl_4_ was bought from Streme and was used as received. An ethylene–propylene gas mixture was purified over trioctylaluminum (0.6 M in mineral spirits) in a bomb reactor (2.0 L). The ^1^H NMR (600 MHz) and ^13^C NMR (150 MHz) spectra were recorded on a JEOL ECZ 600 instrument. Elemental analyses were performed at the Analytical Center of Ajou University. The GPC data were obtained in 1,2,4-trichlorobenzene at 160 °C by means of a PL-GPC 220 system equipped with a refractive-index detector and two columns (PLgel mixed-B 7.5 × 300 mm from Polymer Lab, Salop, UK).

2,6-Dicycloheptylaniline [31]. Zn dust (9.60 g, 0.147 mol) was dried by heating at 200 °C for 5 min in vacuum and was cooled to room temperature under atmospheric N_2_. THF (78 mL) was added to form a gray suspension, which was then cooled to 0 °C. Trimethylsilyl chloride (0.320 g, 2.94 mmol) was added to the solution and stirred at room temperature for 30 min. Bromocycloheptane (13.0 g, 73.4 mmol) was added dropwise at room temperature, and the resulting solution was stirred at 60 °C for 8 h. The solution was filtered to remove a Zn dust excess. The solvent was removed through a vacuum line to obtain clear oil, which was assigned the (cycloheptyl)ZnBr⋅1.4(THF) formula (12.3 g, 69%) through the analysis of the ^1^H NMR spectrum. ^1^H NMR (C_6_D_6_): δ 2.24 (m, 2H), 2.12 (m, 2H), 1.93 (m, 2H), 1.84 (m, 6H), 1.48 (m, 1H, CHZn) ppm. ^13^C NMR (C_6_D_6_): δ 28.66, 30.44, 32.29, 35.67 ppm. 2,6-Dibromoaniline (4.13 g, 16.5 mmol) and Pd-PEPPSI (pyridine-enhanced precatalyst preparation stabilization and initiation)-IHEP^Cl^ cat (76.4 mg, 0.0824 mmol) were mixed in a Schlenk flask, and toluene (65 mL) was added. (Cycloheptyl)ZnBr⋅1.4(THF) (13.5 g, 39.5 mmol) dissolved in THF (20 mL) was added, and the resulting solution was stirred overnight at 40 °C. After cooling to room temperature, the solvent was removed in a rotary evaporator. Diethyl ether (30 mL) was added, and the product was extracted with water (3 × 15 mL). The solvent was removed again in the rotary evaporator. Yellow oil was obtained, which was used without further purification (4.22 g, 90%). ^1^H NMR (C_6_D_6_): δ 7.07 (d, *J* = 7.8 Hz, 2H), 6.93 (t, *J* = 7.8 Hz, 1H), 2.59 (m, 2H, CH), 1.94 (m, 4H), 1.72 (m, 4H), 1.62 (m, 8H), 1.60 (m, 4H), 1.53 (m, 4H) ppm. ^13^C NMR (C_6_D_6_): δ 27.97, 28.21, 35.64, 41.00, 119.13, 123.85, 133.87, 139.62 ppm. High-resolution mass spectrometry (HRMS) (EI): *m/z* calcd. ([M^+^] C_20_H_31_N) 285.2457. Found: 285.2458. 2,6-Dicyclohexylaniline, 2,6-dicyclopentylaniline, and 2,6-di(3-pentyl)aniline were prepared via the same procedure and conditions (see ESI).

Compound **1**. 2,6-Dicycloheptylaniline (1.94 g, 6.78 mmol) and 6-bromo-2-pyridinecarboxaldehyde (1.26 g, 6.78 mmol) were dissolved in toluene (8 mL), and molecular sieves were added. The mixture was heated to 70 °C overnight with stirring. After filtration, the solvent was removed in the rotary evaporator. A yellow solid was obtained, which was used without further purification (2.35 g, 77%). ^1^H NMR (C_6_D_6_): δ 8.42 (s, 1H, NCH), 8.11 (d, *J* = 6.6 Hz, 1H), 7.14 (m, 3H), 6.84 (d, *J* = 8.4 Hz, 1H), 6.64 (t, *J* = 7.2 Hz, 1H), 2.94 (m, 2H), 1.92 (m, 4H), 1.62 (m, 8H), 1.45 (m, 12H) ppm. ^13^C NMR (C_6_D_6_): δ 27.79, 28.21, 36.54, 40.95, 119.38, 124.24, 125.36, 129.83, 138.79, 138.82, 142.56, 147.48, 156.00, 162.28 ppm. HRMS(EI): *m/z* calcd. ([M^+^] C_26_H_33_BrN_2_) 452.1827. Found: 452.1830. Compounds **2**–**6** were prepared by means of the same procedure and conditions (see ESI).

Compound **7**. A Schlenk flask was charged with **1** (2.35 g, 5.18 mmol), 1-naphthylboronic acid (0.936 g, 5.44 mmol), Na_2_CO_3_ (1.45 g, 13.6 mmol), and toluene (10 mL) under N_2_. A degassed H_2_O–EtOH mixture (1:1 [*v/v*], 5 mL) and a solution of (Ph_3_P)_4_Pd (16.2 mg, 0.0140 mmol) in toluene (2 mL) were added next. The biphasic solution was heated at 70 °C overnight with stirring. After cooling to room temperature, water (15 mL) was added, and the product was extracted with toluene (3 × 10 mL). The collected organic phase was dried over anhydrous MgSO_4_, and the solvent was removed in the rotary evaporator. A yellow solid was obtained, which was used without further purification (2.17 g, 84%). ^1^H NMR (C_6_D_6_): δ 8.70 (s, 1H, NCH), 8.44 (d, *J* = 7.8 Hz, 1H), 8.35 (d, *J* = 9.0 Hz, 1H), 7.68 (d, *J* = 7.2 Hz, 1H), 7.65 (d, *J* = 8.4 Hz, 1H), 7.53 (d, *J* = 7.2 Hz, 1H), 7.28 (m, 4H), 7.18 (m, 4H), 3.11 (m, 2H), 2.00 (m, 4H), 1.66 (m, 8H), 1.52 (m, 12H) ppm. ^13^C NMR (C_6_D_6_): δ 27.87, 28.23, 36.66, 40.94, 119.14, 124.22, 125.13, 125.49, 126.16, 126.27, 126.61, 126.73, 128.35, 128.74, 129.39, 131.83, 134.54, 137.18, 138.54, 139.06, 147.97, 155.11, 159.84, 164.29 ppm. HRMS(EI): *m/z* calcd. ([M^+^] C_36_H_40_N_2_) 500.3191. Found: 500.3188. Compounds **8**–**12** were prepared by means of the same procedure and conditions (see ESI).

Compound **13**. 1-Bromo-2-isopropylbenzene (23.0 g, 0.116 mol) in diethyl ether (115 mL) was reacted with n-BuLi (49.7 mL, a 2.5 M solution in hexane, 0.123 mol) for 4 h at room temperature. Volatiles including the solvent and 1-bromobutane were completely removed using a high-vacuum line. The residue was dissolved in hexane (80 mL), and some of the insoluble phase was removed by filtration over Celite. The removal of the solvent afforded 2-isopropylphenyllithium as a white solid, which was used without further purification (13.2 g, 91%). ^1^H NMR (C_6_D_6_): δ 8.39 (m, 1H), 7.38 (m, 3H), 3.24 (septet, *J* = 3.6 Hz, 1H, CH), 1.56 (d, *J* = 7.2 Hz, 6H, CH_3_) ppm. ^13^C NMR (C_6_D_6_): δ 25.81, 40.86, 121.47, 123.64, 125.06, 142.59, 162.00, 182.71 ppm. 2-Isopropylphenyllithium (0.436 g, 3.46 mmol) dissolved in diethyl ether (8 mL) was added dropwise into a Schlenk flask containing **7** (1.00 g, 2.00 mmol) in diethyl ether (20 mL). After stirring for 3 h, an aqueous solution (10 mL) of ammonium chloride (0.30 g) was added, and the product was extracted with diethyl ether (3 × 10 mL). The resulting oil was dried overnight in high vacuum at 60 °C. A yellow solid was obtained, which was used without further purification (0.912 g, 74%). ^1^H NMR (C_6_D_6_): δ 8.24 (m, 1H), 7.82 (m, 1H), 7.63 (m, 1H), 7.61 (d, *J* = 8.4 Hz, 1H), 7.56 (d, *J* = 7.2 Hz, 1H), 7.23 (m, 8H), 7.11 (m, 4H), 5.72 (s, 1H, NCH), 4.46 (s, 1H, NH), 3.27 (septet, *J* = 7.2 Hz, 1H, CH), 2.89 (m, 2H), 1.82 (m, 2H), 1.74 (m, 2H), 1.58 (m, 8H), 1.39 (m, 8H), 1.24 (m, 2H), 1.14 (m, 2H), 1.00 (d, *J* = 6.6 Hz, 3H, CH_3_), 0.98 (d, *J* = 6.0 Hz, 3H, CH_3_) ppm. ^13^C NMR (C_6_D_6_): δ 23.60, 24.53, 27.83, 27.95, 27.98, 29.10, 37.22, 37.50, 40.34, 67.23, 119.93 122.91, 124.31, 124.59, 125.34, 125.77, 126.03, 126.53, 126.58, 126.72, 127.56, 128.53, 129.34, 131.84, 134.63, 136.97, 138.74, 142.09, 142.95, 144.24, 146.46, 159.23, 164.01 ppm. HRMS(EI): *m/z* calcd. ([M^+^] C_45_H_52_N_2_) 620.4130. Found: 620.4128. Compounds **14**–**18** were prepared by the same procedure and under the same conditions (see ESI).

Complex **19**. A Schlenk flask was charged with **13** (0.241 g, 0.388 mmol) in toluene (1.5 g), and n-BuLi (0.25 mL, a 1.6 M solution in hexane, 0.41 mmol) was next added dropwise at room temperature. After stirring for 1 h, HfCl_4_ (0.125 g, 0.390 mmol) was added as a solid. The reaction mixture was heated at 100 °C and stirred for 2 h. After cooling, MeMgBr (0.44 mL, a 3.1 M solution in diethyl ether, 1.4 mmol) was introduced, and the resultant solution was stirred overnight at room temperature. After volatiles were removed using the vacuum line, the product was extracted with toluene (12 mL). The extract was collected through filtration over Celite. After removal of the solvent through the vacuum line, the residue was triturated in hexane (2 mL). A yellow solid was obtained (0.211 g, 66%). ^1^H NMR (C_6_D_6_): δ 8.59 (d, *J* = 7.2 Hz, 1H), 8.34 (d, *J* = 8.4 Hz, 1H), 7.78 (d, *J* = 8.4 Hz, 1H), 7.69 (d, *J* = 6.6 Hz, 1H), 7.58 (d, *J* = 7.2 Hz, 1H), 7.47 (m, 1H), 7.32 (m, 1H), 7.27 (t, *J* = 7.2 Hz, 1H), 7.18 (m, 1H), 7.09 (m, 5H), 6.90 (d, *J* = 7.8 Hz, 1H), 6.64 (d, *J* = 7.8 Hz, 1H), 6.44 (s, 1H, NCH), 3.49 (m, 1H), 3.04 (t, *J* = 10.2 Hz, 1H), 2.89 (septet, *J* = 7.2 Hz, 1H, CH), 2.32 (m, 1H), 2.10 (m, 2H), 1.90 (m, 1H), 1.80 (m, 1H), 1.62 (m, 10H), 1.24 (m, 6H), 1.19 (d, *J* = 7.2 Hz, 3H, CH_3_), 1.13 (m, 2H), 0.98 (s, 3H, HfCH_3_), 0.79 (d, *J* = 6.6 Hz, 3H, CH_3_), 0.69 (s, 3H, HfCH_3_), 0.56 (m, 1H) ppm. ^13^C NMR (C_6_D_6_): δ 23.17, 25.27, 27.17, 27.45, 27.50, 27.62, 28.15, 28.37, 28.89, 28.93, 29.20, 37.01, 38.22, 39.24, 39.57, 40.30, 41.05, 62.44, 66.71, 77.22, 119.61, 120.23, 124.18, 125.30, 125.43, 125.51, 126.04, 126.97, 127.14, 129.94, 130.04, 130.20, 130.85, 134.31, 135.81, 140.70, 141.02, 143.95, 144.35, 146.27, 147.83, 148.19, 164.39, 171.96, 206.43 ppm. Anal. calcd. (C_47_H_56_HfN_2_): C, 68.22; H, 6.82; N, 3.39%. Found: C, 68.44; H, 6.95; N, 3.07%.

Complex **20**. It was prepared by means of the same procedure and conditions as those employed for **19** using **14** (0.150 g, 0.253 mmol), n-BuLi (0.17 mL, a 1.6 M solution in hexane, 0.27 mmol), HfCl_4_ (0.0814 g, 0.254 mmol), MeMgBr (0.29 mL, a 3.1 M solution in diethyl ether, 0.89 mmol), and toluene (1.5 g). A yellow solid was obtained (0.128 g, 63%). ^1^H NMR (C_6_D_6_): δ 8.58 (d, *J* = 7.8 Hz, 1H), 8.29 (d, *J* = 8.4 Hz, 1H), 7.79 (d, *J* = 7.8 Hz, 1H), 7.71 (d, *J* = 7.2 Hz, 1H), 7.54 (d, *J* = 7.8 Hz, 1H), 7.46 (m, 1H), 7.30 (m, 2H), 7.15 (m, 3H), 7.09 (m, 3H), 6.88 (t, *J* = 7.8 Hz, 1H), 6.62 (d, *J* = 8.4 Hz, 1H), 6.48 (s, 1H, NCH), 3.39 (m, 1H), 2.92 (m, 2H), 2.15 (d, *J* = 13.8 Hz, 1H), 2.10 (d, *J* = 13.8 Hz, 2H), 1.80 (m, 2H), 1.65 (m, 3H), 1.29 (m, 6H), 1.17 (d, *J* = 7.2 Hz, 3H, CH_3_), 1.07 (m, 3H), 0.99 (s, 3H, HfCH_3_), 0.95 (m, 2H), 0.73 (d, *J* = 7.2 Hz, 3H, CH_3_), 0.70 (s, 3H, HfCH_3_), 0.23 (m, 1H) ppm. ^13^C NMR (C_6_D_6_): δ 23.31, 25.04, 26.63, 26.74, 27.70, 27.76, 27.81, 28.29, 28.89, 35.00, 35.66, 36.62, 37.02, 38.13, 40.88, 62.53, 67.00, 77.27, 119.30, 120.30, 124.29, 125.52, 125.60, 125.97, 126.95, 127.06, 127.73, 129.91, 130.00, 130.09, 130.85, 134.36, 135.80, 140.73, 140.89, 144.02, 145.12, 146.31, 146.38, 146.49, 164.46, 170.79, 206.40 ppm. Anal. calcd. (C_45_H_52_HfN_2_): C, 67.61; H, 6.56; N, 3.50%. Found: C, 67.98; H, 6.88; N, 3.19%.

Complex **21**. It was prepared via the same procedure and conditions as those described for **19** from **15** (0.300 g, 0.531 mmol), n-BuLi (0.348 mL, a 1.6 M solution in hexane, 0.560 mmol), HfCl_4_ (0.171 g, 0.533 mmol), MeMgBr (0.60 mL, a 3.1 M solution in diethyl ether, 1.9 mmol), and toluene (3.0 g). A yellow solid was obtained (0.278 g, 68%). ^1^H NMR (C_6_D_6_): δ 8.59 (d, *J* = 7.8 Hz, 1H), 8.24 (d, *J* = 8.4 Hz, 1H), 7.81 (d, *J* = 7.2 Hz, 1H), 7.71 (d, *J* = 7.8 Hz, 1H), 7.47 (d, *J* = 8.4 Hz, 1H), 7.30 (m, 3H), 7.21 (m, 2H), 7.11 (m, 2H), 7.01 (m, 2H), 6.80 (t, *J* = 7.8 Hz, 1H), 6.62 (s, 1H, NCH), 6.52 (d, *J* = 7.8 Hz, 1H), 3.74 (m, 1H), 3.55 (quintet, *J* = 8.4 Hz, 1H), 2.90 (septet, *J* = 6.6 Hz, 1H, CH), 2.38 (m, 1H), 2.28 (m, 1H), 2.13 (m, 1H), 1.69 (m, 8H), 1.29 (m, 3H), 1.19 (d, *J* = 7.2 Hz, 3H, CH_3_), 1.04 (m, 1H), 0.92 (s, 3H, HfCH_3_), 0.72 (d, *J* = 6.6 Hz, 3H, CH_3_), 0.70 (s, 3H, HfCH_3_), 0.29 (m, 1H) ppm. ^13^C NMR (C_6_D_6_): δ 23.22, 25.23, 26.23, 26.31, 27.15, 27.45, 28.66, 36.27, 37.46, 38.06, 38.54, 40.40, 41.01, 62.13, 66.83, 119.52, 120.37, 124.24, 125.09, 125.26, 125.51, 125.61, 125.86, 126.17, 126.50, 126.63, 126.95, 129.88, 129.97, 130.00, 130.78, 134.11, 134.30, 135.73, 140.79, 140.87, 144.06, 144.80, 145.93, 146.96, 146.99, 164.46, 170.79, 206.11 ppm. Anal. calcd. (C_43_H_48_HfN_2_): C, 66.96; H, 6.27; N, 3.63%. Found: C, 67.12; H, 6.59; N, 3.42%.

Complex 22. It was prepared via the same procedure and conditions as those chosen for 19 using 16 (0.205 g, 0.361 mmol), n-BuLi (0.24 mL, a 1.6 M solution in hexane, 0.38 mmol), HfCl_4_ (0.116 g, 0.362 mmol), MeMgBr (0.41 mL, a 3.1 M solution in diethyl ether, 1.3 mmol), and toluene (2.0 g). A dark-yellow solid was obtained (0.167 g, 60%). ^1^H NMR (C_6_D_6_): δ 8.61 (d, *J* = 7.8 Hz, 1H), 8.28 (d, *J* = 8.4 Hz, 1H), 7.81 (d, *J* = 7.8 Hz, 1H), 7.72 (d, *J* = 8.4 Hz, 1H), 7.50 (m, 2H), 7.31 (m, 2H), 7.12 (m, 3H), 7.05 (m, 3H), 6.84 (t, *J* = 7.8 Hz, 1H), 6.62 (d, *J* = 8.4 Hz, 1H), 6.53 (s, 1H, NCH), 3.55 (m, 1H, CH), 2.92 (septet, *J* = 7.2 Hz, 1H, CH), 2.76 (m, 1H, CH), 1.94 (m, 1H), 1.77 (m, 5H), 1.48 (m, 1H), 1.18 (d, *J* = 6.6 Hz, 3H, CH_3_), 1.05 (t, *J* = 7.8 Hz, 3H, CH_3_), 1.02 (s, 3H, HfCH_3_), 0.98 (t, *J* = 7.2 Hz, 3H, CH_3_), 0.80 (m, 6H, HfCH_3_, CH_3_), 0.73 (t, *J* = 7.8 Hz, 3H, CH_3_), 0.56 (m, 4H) ppm. ^13^C NMR (C_6_D_6_): δ 11.31, 12.37, 13.39, 13.58, 23.35, 25.33, 27.89, 28.25, 28.77, 31.11, 39.90, 43.27, 63.34, 67.51, 77.52, 119.33, 120.26, 124.25, 124.95, 125.49, 125.53, 125.67, 125.79, 126.93, 127.02, 129.92, 129.99, 130.18, 130.78, 134.48, 135.74, 140.77, 141.35, 143.89, 144.80, 144.89, 146.21, 147.87, 164.29, 170.75, 205.95 ppm. Anal. calcd. (C_43_H_52_HfN_2_): C, 66.61; H, 6.76; N, 3.61%. Found: C, 66.54; H, 6.88; N, 3.80%.

Complex **23**. A Schlenk flask was charged with HfCl_4_ (0.189 g, 0.588 mmol) and toluene (5 mL). After cooling to −78 °C under N_2_ gas, MeMgBr (0.78 mL, a 3.1 M solution in diethyl ether, 2.4 mmol) was added dropwise. The mixture was stirred for 1 h at −40 to −35 °C to precipitate white solids. After cooling to −78 °C again, a solution of **17** (0.190 g, 0.392 mmol) in toluene (5 mL) was introduced dropwise. The resultant mixture was stirred at −40 to −35 °C for 2 h and then warmed slowly to room temperature. After stirring overnight, all volatiles were removed through the vacuum line. Toluene (10 mL) was added to extract the product. The extract was collected by filtration over Celite. After removal of the solvent via the vacuum line, the residue was triturated in hexane (2 mL). A yellow solid was obtained (0.170 g, 63%). ^1^H NMR (C_6_D_6_): δ 8.58 (d, *J* = 7.2 Hz, 1H), 8.35 (d, *J* = 9.0 Hz, 1H), 7.82 (d, *J* = 8.4 Hz, 1H), 7.73 (d, *J* = 7.8 Hz, 1H), 7.56 (d, *J* = 7.8 Hz, 1H), 7.36 (t, *J* = 7.2 Hz, 1H), 7.30 (t, *J* = 6.6 Hz, 1H), 7.22 (d, *J* = 7.8 Hz, 1H), 7.14 (d, *J* = 7.8 Hz, 1H), 7.09 (t, *J* = 7.8 Hz, 2H), 7.04 (m, 2H), 6.93 (t, *J* = 7.8 Hz, 1H), 6.84(t, *J* = 7.8 Hz, 1H), 6.60 (s, 1H, NCH), 6.47 (d, *J* = 7.8 Hz, 1H), 2.83 (m, 4H, CH_2_), 2.41 (m, 1H, CH), 1.30 (t, *J* = 7.8 Hz, 3H, CH_3_), 1.14 (d, *J* = 6.6 Hz, 3H, CH_3_), 0.82 (s, 3H, HfCH_3_), 0.68 (d, *J* = 6.6 Hz, 3H, CH_3_), 0.62 (t, *J* = 7.2 Hz, 3H, CH_3_), 0.56 (s, 3H, HfCH_3_) ppm. ^13^C NMR (C_6_D_6_): δ 14.88, 15.20, 22.85, 24.20, 24.34, 25.57, 28.61, 63.61, 64.75, 74.63, 120.14, 120.30, 124.20, 125.33, 125.54, 126.01, 126.42, 126.71, 126.85, 127.01, 129.91, 130.05, 130.57, 130.70, 134.30, 135.76, 140.67, 140.76, 142.33, 143.79, 143.83, 144.26, 147.16, 164.52, 171.23, 205.38 ppm. Anal. calcd. (C_37_H_40_HfN_2_): C, 64.29; H, 5.83; N, 4.05%. Found: C, 64.41; H, 6.05; N, 3.86%.

Complex **24**. It was prepared by means of the same procedure and conditions as those utilized for **19** from **18** (0.199 g, 0.343 mmol), n-BuLi (0.226 mL, a 1.6 M solution in hexane, 0.362 mmol), HfCl_4_ (0.110 g, 0.345 mmol), MeMgBr (0.39 mL, a 3.1 M solution in diethyl ether, 1.2 mmol), and toluene (2.0 g). A dark-yellow solid was obtained (0.178 g, 66%). ^1^H NMR (C_6_D_6_): δ 8.54 (d, *J* = 7.2 Hz, 1H), 8.33 (d, *J* = 9.6 Hz, 1H), 7.76 (d, *J* = 8.4 Hz, 1H), 7.71 (d, *J* = 8.4 Hz, 1H), 7.57 (d, *J* = 7.8 Hz, 2H), 7.51 (d, *J* = 7.8 Hz, 1H), 7.27 (m, 6H), 7.11 (m, 4H), 7.01 (t, *J* = 7.2 Hz, 1H), 6.96 (d, *J* = 4.2 Hz, 2H), 6.76 (m, 3H), 6.68 (m, 2H), 6.34 (s, 1H, NCH), 6.11 (d, *J* = 7.8 Hz, 1H), 3.15 (septet, *J* = 6.6 Hz, 1H), 1.22 (d, *J* = 6.6 Hz, 3H, CH_3_), 0.93 (m, 6H, HfCH_3,_ CH_3_), −0.08 (s, 3H, HfCH_3_) ppm. ^13^C NMR (C_6_D_6_): δ 23.31, 25.55, 29.13, 64.26, 65.18, 74.07, 118.94, 119.86, 123.91, 124.29, 125.49, 125.74, 126.78, 126.91, 126.94, 127.16, 128.52, 129.69, 130.00, 130.72, 130.75, 131.44, 131.58, 131.95, 134.36, 135.72, 138.33, 139.89, 140.88, 141.19, 141.51, 143.09, 143.65, 146.42, 147.03, 163.90, 170.58, 206.23 ppm. Anal. calcd. (C_45_H_40_HfN_2_): C, 68.65; H, 5.12; N, 3.56%. Found: C, 68.37; H, 5.49; N, 3.25%.

Compound **25**. A Schlenk flask was charged with 2,6-dibromopyridine (7 g, 29.5 mmol), 1-naphthylboronic acid (2.54 g, 14.8 mmol), Na_2_CO_3_ (3.91 g, 36.9 mmol), and toluene (23 mL) in an N_2_ atmosphere. After that, a degassed H_2_O–EtOH mixture (1:1 [*v/v*], 4.67 mL) and a solution of (Ph_3_P)_4_Pd (85.3 mg, 0.0739 mmol) in toluene (5 mL) were added. The biphasic solution was heated at 70 °C and vigorously stirred overnight. After cooling to room temperature, the organic phase was collected and washed with H_2_O (20 mL). The product was extracted with toluene (3 × 20 mL). The collected organic phases were dried over anhydrous MgSO_4_, and the solvent was removed in the rotary evaporator. The product was purified by column chromatography on silica gel using a mixture of hexane and toluene (1:2, *v/v*). A white solid was obtained (3.1 g, 74%). ^1^H NMR (C_6_D_6_): δ 8.18 (d, *J* = 8.4 Hz, 1H), 7.64 (d, *J* = 8.4 Hz, 1H), 7.62 (d, *J* = 8.4 Hz, 1H), 7.44 (d, *J* = 6.6 Hz, 1H), 7.23 (m, 3H), 6.97 (d, *J* = 8.4 Hz, 1H), 6.92 (d, *J* = 7.2 Hz, 1H), 6.68 (t, *J* = 7.8 Hz, 1H) ppm. ^13^C NMR (C_6_D_6_): δ 123.76, 125.41, 125.81, 126.21, 126.30, 126.94, 128.35, 128.70, 129.68, 131.41, 134.35, 137.31, 138.37, 142.22, 160.41 ppm. Anal. calcd. (C_15_H_10_BrN): C, 63.40; H, 3.55; N, 4.93%. Found: C, 63.39; H, 3.66; N, 4.62%.

Compound **26**. Imine compound 2,6-iPr_2_C_6_H_3_N=C(H)Ph was prepared via the same procedure and conditions as those employed for **1** using 2,6-diisopropylaniline (5.01 g, 28.3 mmol) and benzaldehyde (3.00 g, 28.3 mmol). A yellow solid was obtained (6.11 g, 81%). ^1^H NMR (C_6_D_6_): δ 7.95 (s, 1H, NCH), 7.75 (d, *J* = 7.8 Hz, 2H), 7.12 (d, *J* = 7.2 Hz, 2H), 7.07 (m, 4H), 3.08 (septet, *J* = 7.2 Hz, 2H, CH), 1.11 (d, *J* = 6.6 Hz, 12H, CH_3_) ppm. ^13^C NMR (C_6_D_6_): δ 23.61, 28.46, 123.45, 124.66, 128.86, 129.05, 131.51, 136.66, 137.73, 150.15, 162.12 ppm. HRMS(EI): *m/z* calcd ([M^+^] C_19_H_23_N) 265.1830. Found: 265.1830. Compound **25** (0.400 g, 1.41 mmol) was dissolved in THF (8 mL) and cooled to −78 °C. t*-*BuLi (1.7 mL, a 1.7 M solution in hexane, 2.8 mmol) was introduced, and the mixture was stirred for 2 h at −78 °C. A solution of 2,6-iPr_2_C_6_H_3_N=C(H)Ph (0.376 g, 1.41 mmol) in THF (8 mL) was added next. After stirring for 3 h at −78 °C, the resultant solution was slowly warmed to room temperature. After stirring overnight, water (10 mL) was added, and the product was extracted with ethyl acetate (3 × 10 mL). The organic phases were collected and dried over anhydrous MgSO_4_. The solvent was removed in the rotary evaporator. Purification by column chromatography on silica gel using a hexane–toluene mixture containing a small quantity of triethylamine (75:25:1, v/v/v) gave light yellow oil (0.412 g, 63%). ^1^H NMR (C_6_D_6_): δ 8.24 (m, 1H), 7.66 (m, 2H), 7.56 (d, *J* = 7.8 Hz, 3H), 7.28 (m, 3H), 7.05 (m, 8H), 6.77 (d, *J* = 6.6 Hz, 1H), 5.38 (d, *J* = 9.6 Hz, 1H, NCH), 5.34 (d, *J* = 9.6 Hz, 1H, NH), 3.38 (quintet, *J* = 7.2 Hz, 2H, CH), 1.06 (m, 12H, CH_3_) ppm. ^13^C NMR (C_6_D_6_): δ 24.25, 24.49, 28.14, 70.21, 120.82, 123.33, 124.00, 125.46, 126.15, 126.53, 126.62, 127.28, 127.96, 128.58, 128.64, 129.31, 131.85, 134.58, 136.89, 139.03, 142.70, 143.24, 144.06, 159.35, 162.28 ppm. HRMS(EI): *m/z* calcd ([M^+^] C_34_H_34_N_2_) 470.2722. Found: 470.2723. Compounds **27**–**32** were prepared by means of the same procedure and conditions (see SI).

Complex **33**. It was prepared via the same procedure and conditions as those described for **23** from HfCl_4_ (0.208 g, 0.650 mmol), MeMgBr (0.86 mL, a 3.0 M solution in diethyl ether, 2.7 mmol), and 26 (0.204 g, 0.443 mmol). A yellow solid was obtained (0.211 g, 72%). ^1^H NMR (C_6_D_6_): δ 8.57 (d, *J* = 7.2 Hz, 1H), 8.25 (d, *J* = 8.4 Hz, 1H), 7.81 (d, *J* = 7.8 Hz, 1H), 7.72 (d, *J* = 7.8 Hz, 1H), 7.49 (d, *J* = 7.8 Hz, 1H), 7.29 (m, 2H), 7.19 (m, 1H), 7.11 (m, 1H), 7.01 (m, 6H), 6.80 (t, *J* = 8.4 Hz, 1H), 6.39 (d, *J* = 7.8 Hz, 1H), 5.93 (s, 1H, NCH), 3.84 (septet, *J* = 7.2 Hz, 1H), 3.28 (septet, *J* = 6.6 Hz, 1H), 1.40 (m, 6H, CH_3_), 1.16 (d, *J* = 6.6 Hz, 3H, CH_3_), 0.95 (s, 3H, HfCH_3_), 0.67 (s, 3H, HfCH_3_), 0.39 (d, *J* = 7.8 Hz, 3H, CH_3_) ppm. ^13^C NMR (C_6_D_6_): δ 24.38, 24.88, 27.02, 28.09, 28.73, 62.99, 66.87, 83.64, 119.73, 120.55, 124.17, 124.51, 125.26, 125.55, 126.16, 127.00, 127.98, 128.97, 129.10, 129.93, 129.99, 130.71, 134.14, 135.72, 140.89, 143.85, 143.98, 144.88, 146.53, 147.48, 164.41, 169.77, 205.90 ppm. Anal. calcd. (C_36_H_38_HfN_2_): C, 63.85; H, 5.66; N, 4.14%. Found: C, 64.10; H, 5.78; N, 4.00%.

Complex **34**. It was prepared by means of the same procedure and conditions as those utilized for **19** using **27** (0.120 g, 0.207 mmol), n-BuLi (0.129 mL of a 1.6 M solution in hexane, 0.219 mmol), HfCl_4_ (66.7 mg, 0.208 mmol), and MeMgBr (0.24 mL, a 3.0 M solution in diethyl ether, 0.73 mmol). A yellow solid was obtained (0.106 mg, 65%). ^1^H NMR (C_6_D_6_): δ 8.58 (d, *J* = 7.2 Hz, 1H), 8.32 (d, *J* = 8.4 Hz, 1H), 7.78 (d, *J* = 7.2 Hz, 1H), 7.69 (d, *J* = 7.8 Hz, 1H), 7.57 (d, *J* = 7.2 Hz, 1H), 7.32 (m, 1H), 7.27 (m, 1H), 7.20 (m, 3H), 7.05 (m, 5H), 6.87 (t, *J* = 7.8 Hz, 1H), 6.47 (d, *J* = 7.2 Hz, 1H), 5.74 (s, 1H, NCH), 3.46 (m, 1H), 2.92 (m, 1H), 2.33 (m, 1H), 2.11 (m, 2H), 1.89 (m, 1H), 1.82 (m, 1H), 1.62 (m, 9H), 1.25 (m, 9H), 0.97 (s, 3H, HfCH_3_), 0.66 (s, 3H, HfCH_3_), 0.63 (m, 1H) ppm. ^13^C NMR (C_6_D_6_): δ 27.23, 27.44, 27.84, 28.12, 28.28, 28.90, 29.02, 37.49, 37.68, 39.85, 39.93, 40.29, 41.19, 62.50, 66.67, 84.17, 119.73, 120.33, 124.12, 125.33, 125.46, 125.52, 126.16, 127.00, 128.93, 129.16, 129.94, 130.03, 130.80, 134.27, 135.78, 140.78, 143.84, 143.92, 143.95, 148.04, 148.32, 164.51, 170.22, 206.25 ppm. Anal. calcd. (C_44_H_50_HfN_2_): C, 67.29; H, 6.42; N, 3.57%. Found: C, 67.18; H, 6.44; N, 3.31%.

Complex **35**. It was prepared via the same procedure and conditions as those described for **23** using HfCl_4_ (0.124 g, 0.387 mmol), MeMgBr (0.51 mL, a 3.0 M solution in diethyl ether, 1.6 mmol), and **28** (0.142 g, 0.258 mmol). A yellow solid was obtained (0.140 g, 72%). ^1^H NMR (C_6_D_6_): δ 8.56 (d, *J* = 8.4 Hz, 1H), 8.29 (d, *J* = 7.8 Hz, 1H), 7.78 (d, *J* = 7.8 Hz, 1H), 7.70 (d, *J* = 7.8 Hz, 1H), 7.54 (d, *J* = 7.2 Hz, 1H), 7.30 (m, 2H), 7.19 (m, 2H), 7.17 (m, 1H), 7.06 (m, 5H), 6.88 (t, *J* = 7.8 Hz, 1H), 6.43 (d, *J* = 7.2 Hz, 1H), 5.79 (s, 1H, NCH), 3.37 (m, 1H), 2.77 (m, 1H), 2.18 (d, *J* = 12.6 Hz, 1H), 2.07 (m, 2H), 1.84 (d, *J* = 12.0 Hz, 1H), 1.77 (d, *J* = 12.0 Hz, 1H), 1.54 (m, 8H), 1.14 (m, 3H), 0.96 (s, 3H, HfCH_3_), 0.89 (m, 3H), 0.65 (s, 3H, HfCH_3_), 0.34 (m, 1H) ppm. ^13^C NMR (C_6_D_6_): δ 26.65, 26.74, 27.77, 28.28, 35.17, 35.59, 36.23, 38.00, 38.21, 40.83, 62.51, 66.95, 84.22, 119.46, 120.38, 124.19, 125.52, 125.61, 125.79, 126.05, 126.97, 127.76, 128.92, 129.11, 129.92, 130.00, 130.81, 134.33, 135.78, 140.78, 143.87, 144.00, 145.33, 145.79, 146.65, 164.58, 170.16, 206.09 ppm. Anal. calcd. (C_42_H_46_HfN_2_): C, 66.61; H, 6.12; N, 3.70%. Found: C, 66.89; H, 6.45; N, 3.51%.

Complex **36**. It was prepared by means of the same procedure and conditions as those chosen for **23** using HfCl_4_ (0.184 g, 0.587 mmol), MeMgBr (0.76 mL, a 3.0 M solution in diethyl ether, 2.4 mmol), and **29** (0.200 g, 0.383 mmol). A yellow solid was obtained (0.226 g, 81%). ^1^H NMR (C_6_D_6_): δ 8.57 (d, *J* = 7.8 Hz, 1H), 8.24 (d, *J* = 8.4 Hz, 1H), 7.80 (d, *J* = 7.8 Hz, 1H), 7.71 (d, *J* = 7.2 Hz, 1H), 7.47 (d, *J* = 8.4 Hz, 1H), 7.27 (m, 3H), 7.19 (t, *J* = 7.8 Hz, 1H), 7.14 (m, 1H), 7.01 (m, 5H), 6.80 (t, *J* = 4.2 Hz, 1H), 6.40 (d, *J* = 7.8 Hz, 1H), 5.93 (s, 1H, NCH), 3.74 (m, 1H), 3.43 (quintet, *J* = 9.6 Hz, 1H), 2.40 (m, 1H), 2.20 (m, 1H), 2.10 (m, 1H), 1.69 (m, 8H), 1.33 (m, 3H), 1.06 (m, 1H), 0.91 (s, 3H, HfCH_3_), 0.66 (s, 3H, HfCH_3_), 0.34 (m, 1H) ppm. ^13^C NMR (C_6_D_6_): δ 26.18, 26.22, 26.86, 27.19, 36.74, 37.53, 39.17, 40.67, 41.04, 62.26, 66.85, 84.05, 119.56, 120.49, 124.17, 125.10, 125.51, 125.85, 126.26, 126.96, 128.91, 129.04, 129.91, 129.98, 130.74, 134.15, 135.73, 140.82, 143.94, 144.04, 145.01, 145.67, 146.68, 164.60, 170.03, 205.87 ppm. Anal. calcd. (C_40_H_42_HfN_2_): C, 65.88; H, 5.80; N, 3.84%. Found: C, 65.94; H, 5.72; N, 3.75%.

Complex **37**. It was prepared by means of the same procedure and conditions as those employed for **23** from HfCl_4_ (0.0709 g, 0.221 mmol), MeMgBr (0.29 mL, a 3.0 M solution in diethyl ether, 0.91 mmol), and **31** (0.0653 g, 0.148 mmol). A yellow solid was obtained (0.0628 g, 66%). ^1^H NMR (C_6_D_6_): δ 8.57 (d, *J* = 7.2 Hz, 1H), 8.35 (d, *J* = 8.4 Hz, 1H), 7.82 (d, *J* = 7.8 Hz, 1H), 7.73 (d, *J* = 8.4 Hz, 1H), 7.56 (d, *J* = 8.4 Hz, 1H), 7.36 (t, *J* = 7.2 Hz, 1H), 7.30 (t, *J* = 7.8 Hz, 1H), 7.23 (d, *J* = 7.2 Hz, 1H), 7.18 (m, 1H), 7.12 (d, *J* = 7.8 Hz, 1H), 6.96 (m, 3H), 6.83 (m, 3H), 6.36 (d, *J* = 7.8 Hz, 1H), 5.93 (s, 1H, NCH), 2.84 (sextet, *J* = 7.2 Hz, 1H, CH_2_), 2.78 (sextet, *J* = 7.8 Hz, 1H, CH_2_), 2.69 (sextet, *J* = 6.6 Hz, 1H, CH_2_), 2.38 (sextet, *J* = 6.6 Hz, 1H, CH_2_), 1.32 (t, *J* = 7.8 Hz, 3H, CH_3_), 0.80 (s, 3H, HfCH_3_), 0.61 (t, *J* = 7.2 Hz, 3H, CH_3_), 0.55 (s, 3H, HfCH_3_) ppm. ^13^C NMR (C_6_D_6_): δ 15.28, 15.68, 24.37, 63.72, 64.58, 81.45, 120.29, 120.49, 124.13, 125.56, 126.09, 126.69, 126.74, 127.05, 127.82, 128.76, 129.38, 129.90, 130.06, 130.65, 134.32, 135.74, 140.68, 142.53, 143.15, 143.73, 144.00, 144.24, 164.59, 170.07, 205.27 ppm. Anal. calcd. (C_34_H_34_HfN_2_): C, 62.91; H, 5.28; N, 4.32%. Found: C, 63.13; H, 5.50; N, 4.41%.

A typical CCTP. A bomb reactor (125 mL) was evacuated at 60 °C for 1 h. After charging with ethylene gas at atmospheric pressure, a solution of Me_3_Al (28.8 mg, 200 µmol-Al) in methylcyclohexane (15.5 g) was added to the reactor. The mixture was stirred for 1 h at 100 °C using a mantle, and the solution was subsequently removed using a cannula. The reactor was evacuated once more to remove any residual solvent and was re-charged with ethylene gas at atmospheric pressure. This procedure was performed to clean up any catalyst poisons. The reactor was charged with methylcyclohexane (15.5 g), which contains MMAO (AkzoNobel, 6.7 wt%-Al in heptane, 20 mg, 50 µmol-Al) and the temperature was set to 80 °C. A solution of (1-hexyl)_2_Zn (150, 300, or 450 µmol) in methylcyclohexane (10.0 g) was charged; subsequently, the methylcyclohexane solution (0.30 g) containing a Hf complex (1.0 µmol-Hf) that was activated with [(C_18_H_37_)_2_N(H)Me]^+^[B(C_6_F_5_)_4_]^−^ (1.0 eq) in benzene, was injected. Ethylene/propylene mixed gas (15 bar/10 bar, total 25 bar) was charged from a tank into the reactor at 23 bar and the polymerization was performed for 70 min. Temperature rose spontaneously to ~90 °C within 1 min due to exotherm and then gradually decreased reaching ~60 °C in 70 min. Heat was not given externally during the polymerization time. After performing polymerization for 70 min, the remaining ethylene/propylene mixed gas was vented off. The generated polymer was collected and dried in a vacuum oven at 160 °C overnight. Polymer sample was dissolved in a mixture of C_6_D_6_ and 1,2,4-trichlorobenzene (*v/v*, 1:3) and ^1^H NMR spectrum was recorded at 70 °C. Methyl (CH_3_) signal was observed at 0.88–0.95 ppm separated from those of methylene (CH_2_) and methine (CH) at 1.10–1.50 ppm. The propylene mole fraction (*F*_C3_) in the poly(ethylene-*co*-propylene) was calculated by the equation: *F*_C3_ = (*I*_CH3_/3)/[(*I*_CH2+CH_—*I*_CH3_)/4 + (*I*_CH3_/3)] where *I*_CH3_ and *I*_CH2+CH_ are integration values at 0.88–0.95 and 1.10–1.50 regions, respectively.

Ethylene polymerization with flowmeter. To a bomb reactor cleaned by the aforementioned procedure methylcyclohexane (15.5 g) containing MMAO (AkzoNobel, 6.7 wt%-Al in heptane, 20 mg, 50 µmol-Al), a solution of (1-hexyl)_2_Zn (500 µmol) in methylcyclohexane (10.0 g), and the methylcyclohexane solution (0.30 g) containing a Hf complex (1.0 µmol-Hf) that was activated with [(C_18_H_37_)_2_N(H)Me]^+^[B(C_6_F_5_)_4_]^−^ (1.0 eq) in benzene were successively injected. After the injection of catalyst, ethylene gas was immediately charged directly through a bypass line of mass flow controller (MFC) at a constant pressure (10 bar). After 5 min, bypass line was blocked, and ethylene gas was charged through a line attached with MFC to monitor the ethylene consumption. Temperature rose spontaneously to 91 °C within several minutes due to exotherm and then gradually decreased reaching 80 °C, from which the temperature was controlled at 80 °C with a controller.

High-temperature GPC studies. Sample solutions (200 μL) with concentrations of 3000 ppm were eluted in 1,2,4-trichlorobenzene at a flow rate of 1.0 mL/min at 160 °C. The mobile phase was stabilized with 2,6-di-*tert*-butyl-4-methylphenol (0.04%). The PS-based molecular weight distributions were calculated from a calibration curve on the basis of narrow PS standards. For calculation of PE-based molecular weight distributions, the PS standard molecular weights (*M*_PS_) were converted to PE equivalents (*M*_PE_) using the reported Mark–Houwink–Sakurada parameters for PS (K = 0.000121; a = 0.707) and PE (K = 0.000406; a = 0.725) using the equation *M*_PE_ = [(0.000121/0.000406) × *M*_PS_^(1+0.707)^]^(1/(0.725+1))^ = 0.495 × *M*_PS_. In the case of the poly(ethylene-*co*-propylene) samples, the converted *M*_PE_ values were further converted to PO equivalents using the equation: *M*_PO_ = *M*_PE_/(1−S) where S is the mass fraction of the CH_3_-side chains (i.e., S = (15 × *F*_C3_)/[(1—*F*_C3_) × 28 + (*F*_C3_ × 42)]) [20,50].

X-ray crystallography. Reflection data on 21 (1981978), 23 (1981979), 34 (1981980), and 36 (1981981) were collected on a Bruker APEX II CCD area diffractometer using graphite-monochromated Mo K–α radiation (λ = 0.7107 Å). Specimens of suitable quality and size were selected, mounted, and centered in the X-ray beam with the help of a video camera. The hemisphere of the reflection data was collected as φ and ω scan frames at 0.5°/frame and an exposure time of 10 s/frame. The cell parameters were determined and refined in the SMART software. Data reduction was performed using the SAINT software. The data were corrected for Lorentz and polarization effects. An empirical absorption correction was applied in the SADABS software. The structures of the compounds were solved by direct methods and refined by full-matrix least-squares methods using the SHELXTL software suite with anisotropic thermal parameters for all nonhydrogen atoms. The crystallographic data were deposited with the Cambridge Crystallographic Data Centre.

Crystallographic data on compound 21. C_43_H_48_HfN_2_, M = 771, monoclinic, *a* = 42.213 (2), *b* = 9.3617 (4), *c* = 18.1494 (9) Å, β = 99.578 (5)°, *V* = 7072.4 (6) Å^3^, *T* = 100 (2) K, space group *C*2*/c*, *Z* = 8, 6797 unique [R(int) = 0.1092], which were used in all calculations. Final *wR*_2_ was 0.1565 [*I* > 2σ(*I*)].

Data on 23. C_37_H_40_HfN_2_, *M* = 691.20, monoclinic, *a* = 8.65300 (10), *b* = 9.1676 (2), *c* = 19.3308(3) Å, α = 95.7443 (8), β = 95.6613 (8),γ = 97.7036 (8)°, *V* = 1502.19 (4) Å^3^, *T* = 100 (2) K, space group *P*-1, *Z* = 2, 7203 unique [R(int) = 0.0195], which were used in all calculations. Final *wR*_2_ was 0.0420 [*I* > 2σ(*I*)].

Data on 34. C_47_H_53_HfN_2_, *M* = 824.40, monoclinic, *a* = 9.7456 (4), b = 18.0067(8), *c* = 21.7878(9) Å, β = 92.225(3)°, *V* = 3820.6(3) Å^3^, *T* = 100 (2) K, space group *P*2*_1_/n*, *Z* = 4, 7267 unique [R(int) = 0.0961], which were used in all calculations. Final *wR*_2_ was 0.1000 [*I* >2σ(*I*)].

Data on 36. C_40_H_42_HfN_2_, *M* = 729.28, monoclinic, *a* = 9.5311 (2), *b* = 21.5930 (6), *c* = 16.0250 (4) Å, β = 95.1668 (15)°, *V* = 3284.63 (14) Å^3^, *T* = 100 (2) K, space group *P2_1_/n*, *Z* = 4, 6056 unique [R(int) = 0.0428], which were used in all calculations. Final *wR*_2_ was 0.0602 [*I* > 2σ(*I*)].

## 3. Results and Discussion

### 3.1. Preparation of Hf Complexes

**Scheme 2:**
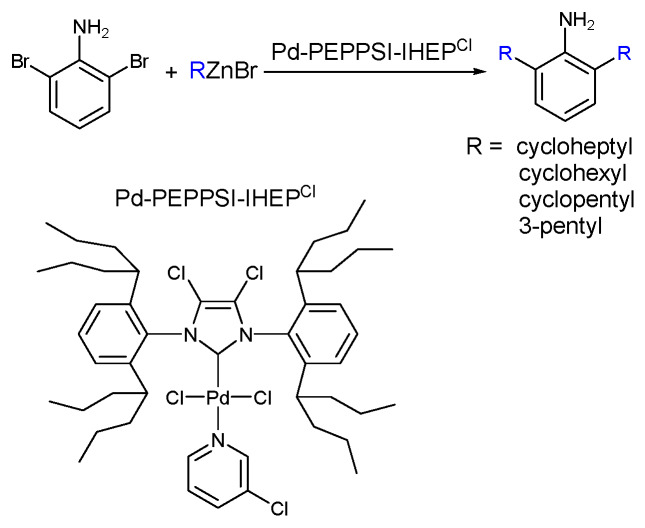
The synthesis of aniline compounds.

**Scheme 3:**
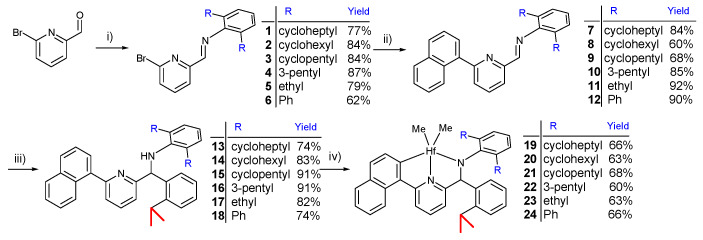
Synthesis of Hf complexes: (**i**) 2,6-R_2_-aniline; (**ii**) 2-naphthylboronic acid, (Ph_3_P)_4_Pd; (**iii**) 2-iPrC_6_H_4_Li; and (**iv**) n-BuLi, HfCl_4_, and MeMgBr (3.5 eq) for **19**–**22** and **24**; HfMe_4_ for **23**.

2,6-R_2_-Anilines were prepared from 2,6-dibromoaniline by the Negishi coupling reaction with various RZnBr compounds (R = cycloheptyl, cyclohexyl, cyclopentyl, or 3-pentyl) using a PdCl_2_ complex coordinated by NHC ligands bearing 2,6-di(3-heptyl)phenyl-N moieties as a catalyst (Scheme 2; see Figures S1–S4 for ^1^H and ^13^C NMR spectra) [31]. 2,6-Diphenylaniline was prepared by Suzuki coupling with PhB(OH)_2_ by means of the Pd(OAc)_2_ catalyst. Using 2,6-R_2_-anilines (R = cycloheptyl, cyclohexyl, cyclopentyl, 3-pentyl, ethyl, or Ph), a series of derivatives of **I** was prepared according to the synthetic scheme disclosed in a patent filed by Dow (Scheme 3). Thus, the starting material 6-bromo-2-pyridinecarboxaldehyde was converted to imine compounds **1**–**6** through condensation with aniline derivatives, and then the Suzuki coupling reaction was carried out with naphthylboronic acid (see Figures S5–S10 for ^1^H and ^13^C NMR spectra). The resulting 2-naphthylpyridyl imine compounds **7**–**12** (see Figures S11–S16 for ^1^H and ^13^C NMR spectra) were reacted with 2-isopropylphenyllithium to obtain target ligands **13**–**18** (see Figures S17–S22 for ^1^H and ^13^C NMR spectra). 2-Isopropylphenyllithium was generated from 1-bromo-2-isopropylbenzene by treatment with n-butyllithium (n-BuLi) in diethyl ether, which had to be isolated before use via thorough removal of the solvent and byproduct 1-bromobutane in vacuum.

**Figure 1:**
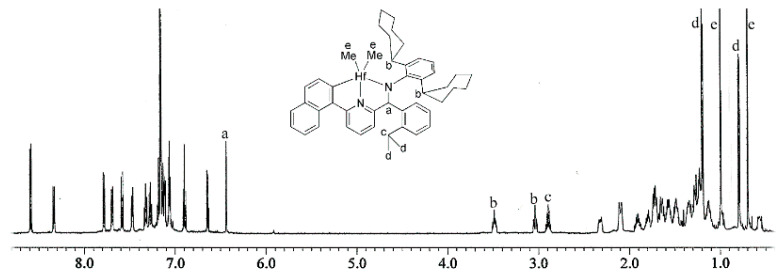
The 1H NMR spectrum of **19.**

Reactions of metalation of the ligands containing a cycloheptyl, cyclohexyl, cyclopentyl, 3-pentyl, or phenyl substituent (**13**–**16** and **18**) were successfully carried out by sequential treatment with n-BuLi in toluene at room temperature, with HfCl_4_ at 100 °C for 2 h, and finally with 3.5 eq of MeMgBr at room temperature. The yields were satisfactorily high (60%–68%). The same treatment of the ligand carrying an ethyl substituent (**17**) did not afford the desired complexes. However, the desired complex (**23**) was obtained in high yield (65%) when **17** was treated with HfMe_4_ generated in situ in the reaction of HfCl_4_ with 4 eq of MeMgBr at −35 °C [51]. The product was isolated as a light-yellow solid through trituration in hexane. In a ^1^H NMR spectrum of **I**, two sets of signals were noted, which were attributed to the presence of a rotamer at ~7 mol% owing to restricted rotation around the NC−C_6_H_4_(2-iPr) bond [8]. In the case of **23** (bearing a small ethyl substituent), such rotamer signals were observed, but in the other complexes, only one set of signals was noted (Figure 1 and Figures S23–S28). Two singlet signals assigned to Hf-CH_3_ were observed in regions 0.0–1.0 and 62–67 ppm in the ^1^H and ^13^C NMR spectra, respectively.

**Scheme 4:**
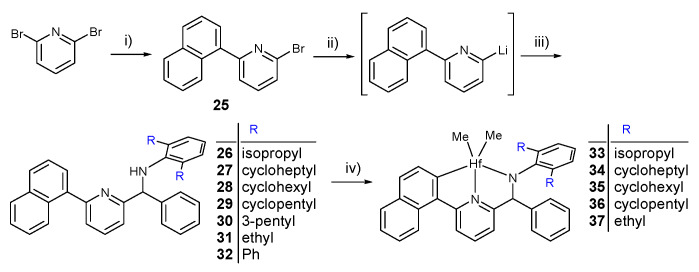
The synthesis of Hf complexes: (**i**) 2-naphthylboronic acid, (Ph_3_P)_4_Pd; (**ii**) t-BuLi (2 eq); (**iii**) 2,6-R_2_C_6_H_3_N=C(H)Ph; and (**iv**) HfMe_4_ for **33** and **35**–**37**; n-BuLi, HfCl_4_, and MeMgBr (3.5 eq) for **34**.

6-Bromo-2-pyridinecarboxaldehyde and 1-bromo-2-isopropylbenzene used in the syntheses of **I** and **19**–**24** are expensive chemicals; accordingly, a route based on inexpensive chemicals was designed for the synthesis of derivatives of **I** that (in contrast) contain the simple PhC(H)- moiety instead of the 2-iPrC_6_H_4_C(H)-part (Scheme 4). Thus, 2,6-dibromopyridine was reacted with 2-naphthylboronic acid to prepare 2-bromo-6-naphthylpyridine (**25**; see Figure S29 for ^1^H and ^13^C NMR spectra), which was treated with 2 eq of t-BuLi to generate 2-lithio-6-naphthylpyridine. The resultant lithio compound was in situ reacted with various imine compounds [2,6-R_2_C_6_H_3_N=C(H)Ph, R = isopropyl, cycloheptyl, cyclohexyl, cyclopentyl, 3-pentyl, ethyl, or Ph] to obtain desired ligands **26**–**32** (see Figures S30–S36 for ^1^H and ^13^C NMR spectra). Imine compounds were prepared simply by reacting benzaldehyde with various 2,6-R_2_C_6_H_3_NH_2_. An attempt to prepare an Hf complex with **26** according to the method employed for **I** (i.e., sequential treatment with n-BuLi, HfCl_4_, and MeMgBr) was unsuccessful. Nevertheless, another method (the one described for the synthesis of **23**, i.e., treatment with in situ–generated HfMe_4_) afforded desired complex **33** in a 72% yield (see Figure S37 for ^1^H and ^13^C NMR spectra). Complexes **35**–**37** bearing a cyclohexyl, cyclopentyl, or ethyl substituent were also synthesized in a high yield (66%–72%) by the same treatment with in situ–generated HfMe_4_, but **34** containing the bulkiest cycloheptyl substituents was not cleanly obtained by the same method (see Figures S38–S41 for ^1^H and ^13^C NMR spectra). On the other hand, sequential treatment of **27** with n-BuLi, HfCl_4_, and MeMgBr afforded desired complex **34**. For **30** and **32**, neither the sequential treatment with n-BuLi, HfCl_4_, and MeMgBr nor the treatment with in situ–generated HfMe_4_ gave the desired complexes. In ^1^H and ^13^C NMR spectra of all these complexes, **33**–**37**, a single set of signals that is assignable to each structure was observed (Figures S37–S41).

### 3.2. X-ray Crystallographic Analyses

**Figure 2:**
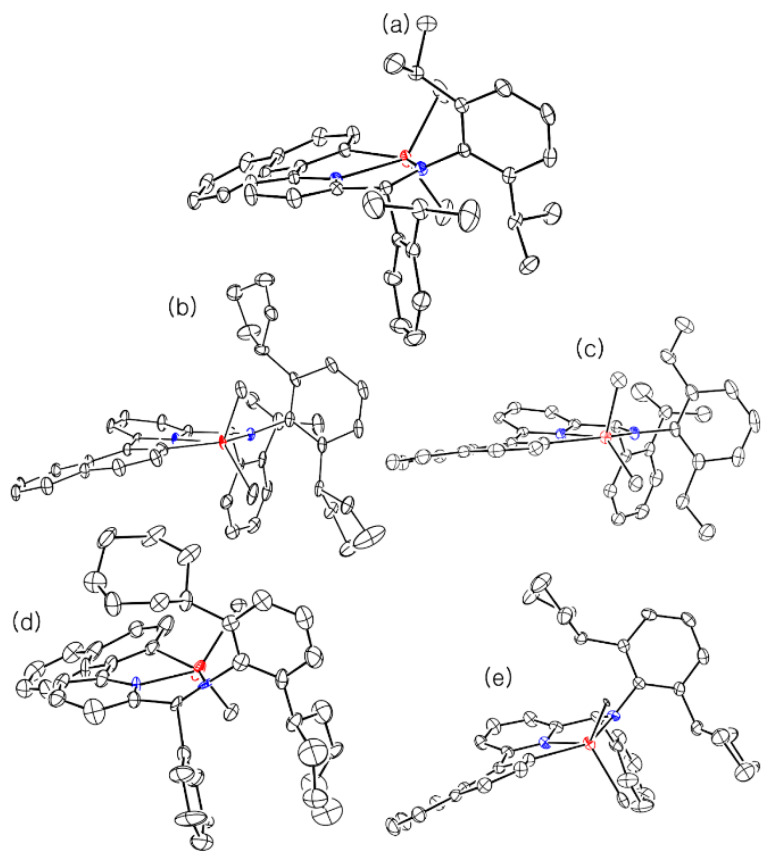
Thermal ellipsoid plots (a 30% probability level) of **I** (**a**), **21** (**b**), **23** (**c**), **34** (**d**), and **36** (**e**). Hydrogen atoms are omitted for clarity.

Single crystals of **21**, **23**, **34**, and **36** were grown in methylcyclohexane solution. Structures determined by X-ray crystallography are presented in Figure 2 as compared with the structure of **I** [8]. Geometrical parameters are compared and summarized in Table 1. All complexes had a distorted trigonal bipyramidal structure; nitrogen in pyridine (N^pyridine^), two coordinating methyl carbons (CH_3_), and Hf formed a plane (the sum of bond angles around Hf is 359°), while amido nitrogen (N^amido^) and a coordinating carbon in naphthyl (C^naphthyl^) occupied the axial sites with distortion (the bond angle of N^amido^-Hf-C^naphthyl^ is 140°). Hf-C^naphthyl^, Hf-N^amido^, and Hf-N^pyridine^ distances were almost unaffected by the substituents, being 2.25–2.27, 2.07–2.08, and 2.30–2.31 Å, respectively.

Some geometrical differences were observed between the complexes bearing the 2-iPrC_6_H_4_C(H)-moiety (**I**, **21**, and **23**) and the ones containing the simple PhC(H)-moiety (**34** and **36**). The amido nitrogen atoms in **I**, **21**, and **23** underwent sp^2^ hybridization (trigonal geometry) for π-donation from nitrogen to Hf, as inferred from the measurement of bond angles around N^amido^; the sum of bond angles around N^amido^ was 359–361°. In the cases of **34** and **36**, the sum of bond angles around N^amido^ was 356–357°, somewhat deviating from the ideal value (360°) expected for trigonal geometry. The Hf-CH_3_ bond distances in **I**, **21**, and **23** were almost invariably identical (2.21–2.23 Å), whereas those distances in **34** and **36** were relatively long and varied with the substituent and even within each structure (2.22 and 2.25 Å in **34**; 2.27 and 2.33 Å in **36**). Chelating frameworks deviated less from a plane for the complexes bearing the 2-iPrC_6_H_4_C(H)-moiety, and the distortion was the lowest for **I**. N^pyridine^−C^pyridine^−C^naphthyl^−C^naphthyl^(Hf) dihedral angles are 13–16° for **I**, **21**, and **23** (the smallest value of 13° for **I**), whereas those angles observed for **34** and **36** were relatively large: 18–20°. The N^pyridine^−C^pyridine^−CH−N^amido^ dihedral angles for **I**, **21**, and **23** were also smaller (9–11°) than those in **34** and **36** (15−18°). Overall, Hf atoms slightly deviated from coplanarity with the pyridine plane (pyridine plane−Hf distances: 0.25–0.69 Å). The deviation was the lowest for **I** (0.25 Å). Naphthyl planes also deviated from coplanarity with the pyridine ring, and the deviations were less severe for the complexes carrying the 2-iPrC_6_H_4_C(H)- moiety; angles between the pyridine and naphthyl planes were 19−23° for **I**, **21**, and **23** (the smallest for **I**, 19°), whereas those angles observed in **34** and **36** weere 26°. The isopropyl group in the 2-iPrC_6_H_4_C(H)-moiety was situated in a plane formed by the chelating ligands (i.e., Hf−N^amido^−CH---CH(Me)_2_ dihedral angles were 172–180°) exerting steric repulsion on a substituent in the 2,6-R_2_C_6_H_3_-moiety and consequently pushing the N−C(2,6-R_2_C_6_H_3_) vector nearly parallel to the chelation plane. In other words, the N^pyridine^−Hf−N^amido^−C(2,6-R_2_C_6_H_3_) dihedral angle was very acute (9−11°), and the N−C(2,6-R_2_C_6_H_3_) bond was rather staggered with Hf−CH_3_ bonds in **I**, **21**, and **23**. In the absence of the isopropyl substituent (i.e., in **34** and **36**), the N−C(2,6-R_2_C_6_H_3_) vector was tilted from the chelation plane; i.e., the N^pyridine^−Hf−N^amido^−C(2,6-R_2_C_6_H_3_) dihedral angles were somewhat greater (15−18°), and the N−C(2,6-R_2_C_6_H_3_) bond was eclipsed with a Hf−CH_3_ bond (the H_3_C−Hf−N^amido^−C^aryl^ dihedral angle: 8−12°).

### 3.3. Polymerization Experiments

**Figure 3:**
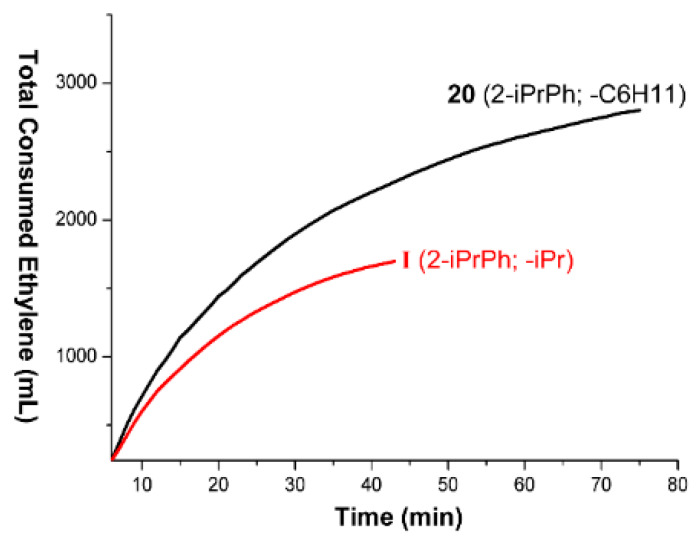
Ethylene consumption monitored with a mass flow controller.

The prepared complexes along with comparison compound **I** were screened using 1.0 µmol of the Hf complex as a catalyst and 150 µmol of (hexyl)_2_Zn as a chain transfer agent under identical conditions after Hf complexes were activated with anhydrous [(C_18_H_37_)_2_N(H)Me]^+^[B(C_6_F_5_)_4_]^−^ [5,7,28]. Catalysts derived from **19** and **20** bearing the 2-iPrC_6_H_4_C(H)-moiety and a cycloheptyl or cyclohexyl substituent survived for longer periods than did the catalyst derived from **I**, as was expected, and, consequently, those complexes (**19** and **20**) manifested somewhat higher activity than **I** did (10 vs. 8.5 g). The rate of monomer consumption decreased with time in all cases, but the time that it took for the rate to become negligible was ~40 min for **I** and ~70 min for **19** and **20**. In a separate experiment, in which ethylene instead of the ethylene–propylene gas mixture was fed under constant pressure (10 bar) to monitor the ethylene consumption rate with a MFC, it was clearly demonstrated that the catalyst derived from **20** had longer lifetime (~70 min) with higher activity in comparison with **I** (~40 min; Figure 3).

Compound **21** bearing the cyclopentyl substituent showed activity similar to that of **I**, whereas **22** and **23** containing the 3-pentyl or ethyl substituent featured lower activities than **I** did (5.6 and 5.8 vs. 8.5 g, respectively). Complex **24** bearing the 2,6-Ph_2_C_6_H_3_N-substituent was inactive. All the complexes bearing the PhC(H)-moiety instead of 2-iPrC_6_H_4_C(H)-had lower activity than **I** did. The highest activity was seen in **34** bearing the bulkiest 2,6-(cycloheptyl)_2_C_6_H_3_N-substituent (7.0 g) among the complexes containing the PhC(H)-moiety, whereas the lowest activity was noted in **37** carrying the smallest 2,6-Et_2_C_6_H_3_N-substituent (3.9 g). Conclusively, bulky substituent is crucially required to attain high activity. Bulky substituent may make the interactions between the cationic Hf center and anionic borate as well as between the Hf center and coordinated carbons loose, consequently leading to high activity [52,53]. The complexes containing the 2-iPrC_6_H_4_C(H)-moiety (**I** and **19**−**23**) incorporated a larger amount of propylene than did complexes **33**−**37** bearing the PhC(H)-moiety (*F*_C3_, 0.18−0.20 vs. 0.14−0.16, respectively).

(Hexyl)_2_Zn worked well as a chain transfer agent in all cases. Polymer chains grew uniformly from all the fed (hexyl)_2_Zn, as inferred from the finding that the *M*_n_ values calculated via the yield (g)/(2 × Zn (mol)) formula were in good agreement in all cases with the *M*_n_ values measured by gel permeation chromatography (GPC) with PS standards and data converted to PO equivalents through universal calibration: *M*_PO_ = 0.495 × *M*_PS_^0.990^/(1−S), where S is the mass proportion of the CH_3_ side chains, i.e., S = (15 × [C_3_H_6_])/[(1−[C_3_H_6_]) × 28 + ([C_3_H_6_] × 42)] [14]. Molecular-weight distributions were fairly narrow too (*M*_w_/*M*_n_ of 1.4−1.9). In addition, *M*_n_ values were quantitatively lowered by the increase in the fed amount of (hexyl)_2_Zn, and *M*_n_ values calculated by means of the yield (g)/(2 × Zn (mol)) formula were also in agreement with the measured *M*_n_ values (14 and 11 vs. 12 and 9.0 kDa, respectively; entries 13 and 14 in Table 2).

## 4. Conclusions

With an aim to block a possible deactivation process in prototype pyridylamido Hf complex **I** discovered at Dow for CCTP, its derivatives were prepared according to a reported synthetic route by replacing the 2,6-diisopropylphenylamido part with various 2,6-R_2_C_6_H_3_N-moieties (R = cycloheptyl, cyclohexyl, cyclopentyl, 3-pentyl, ethyl, or Ph). Another series of derivatives, in which both 2-iPrC_6_H_4_C(H)-and 2,6-diisopropylphenylamido parts in **I** were replaced with PhC(H)-and various 2,6-R_2_C_6_H_3_N-moieties (R = cycloheptyl, cyclohexyl, cyclopentyl, or ethyl), respectively, was prepared too after we devised a new synthetic route obviating expensive chemicals. X-ray crystallographic analyses revealed that the isopropyl substituent in the 2-iPrC_6_H_4_C(H)-part strongly influences the geometry of the structure. The two Hf-CH_3_ distances are similar in each complex bearing the 2-iPrC_6_H_4_C(H)-moiety, whereas those distances are different for complexes containing the PhC(H)-moiety. Chelating frameworks deviate less from a plane, and the N−C(2,6-R_2_C_6_H_3_) bond was found to be staggered with Hf−CH_3_ bonds in the complexes carrying the 2-iPrC_6_H_4_C(H)-moiety but is eclipsed in complexes bearing the PhC(H)-moiety. The isopropyl substituent in the 2-iPrC_6_H_4_C(H)-moiety also influences the catalytic performance in CCTP. The activity was reduced via replacement of the iPrC_6_H_4_C(H)-part with the PhC(H)-moiety in all cases, and the ability to incorporate α-olefin was also inferior for complexes containing the PhC(H)-moiety. After replacement of the 2,6-diisopropylphenylamido part in **I** with the 2,6-di(cycloheptyl)phenylamido or 2,6-di(cyclohexyl)phenylamido moiety, the activity somewhat increased, and the lifetime of the activated catalyst was longer. Polyolefin chains grew uniformly in all cases from (hexyl)_2_Zn fed as a chain transfer agent, as inferred from the agreement between measured *M*_n_ and expected *M*_n_ calculated by means of the yield (g)/(2 × Zn (mol)) formula.

## Figures and Tables

**Table 1:** Bond distances (Å) and angles (°) determined by X-ray crystallography.

	I	21	23	34	36
Hf−CH_3_	2.210 (3)	2.215 (14)	2.224 (2)	2.219 (5)	2.273 (3)
	2.223 (3)	2.212 (11)	2.232 (2)	2.250 (5)	2.326 (3)
Hf−C^naphthyl^	2.256 (2)	2.251 (9)	2.264 (2)	2.276 (5)	2.265 (4)
Hf−N^amido^	2.081 (2)	2.073 (8)	2.071 (2)	2.070 (4)	2.067 (3)
Hf−N^pyridine^	2.295 (2)	2.310 (8)	2.306 (2)	2.302 (4)	2.300 (3)
pyridine plane−Hf	0.2491 (1)	0.5380 (4)	0.6926 (2)	0.3915 (4)	0.4810 (1)
H_3_C−Hf−CH_3_	105.7 (2)	104.8 (5)	104.16 (9)	108.3 (2)	104.9 (1)
H_3_C−Hf−N^pyridine^	134.2 (1)	130.9 (5)	116.13 (8)	112.4 (2)	136.8 (1)
	119.2 (1)	123.5 (4)	138.58 (8)	138.0 (2)	117.6 (1)
N^amido^−Hf−C^naphthyl^	140.63 (8)	141.7 (3)	140.60 (7)	139.8 (2)	139.5 (1)
C^aryl^−N^amido^−Hf	124.7 (1)	125.5 (6)	119.2 (1)	118.3 (3)	120.3 (2)
C^aryl^−N^amido^−CH	110.8 (2)	110.0 (7)	113.8 (2)	114.4 (4)	112.2 (3)
Hf−N^amido^−CH	123.7 (1)	125.5 (6)	125.9 (1)	124.6 (3)	123.9 (2)
N^pyridine^−C^pyridine^−C^naphthyl^−C^naphthyl^(Hf)	12.8 (3)	15.9 (1)	14.4 (2)	19.9 (6)	17.7 (4)
N^pyridine^−C^pyridine^−CH−N^amido^	9.5 (2)	11.3 (1)	9.2 (2)	15.4 (6)	18.3 (4)
N^pyridine^−Hf−N^amido^−C^aryl^	161.0 (2)	160.2 (7)	174.4 (2)	148.2 (4)	143.6 (3)
H_3_C−Hf−N^amido^−C^aryl^	28.5 (2)	39.5 (8)	61.4 (2)	12.1 (4)	8.2 (2)
	83.3 (2)	71.5 (8)	49.4 (2)	103.5 (3)	102.5 (2)
Hf−N^amido^−CH---CH(Me)_2_	175.2 (1)	179.9 (4)	171.6 (1)	−	−
pyridine plane−naphthyl plane	19.31 (7)	22.9 (3)	22.90 (6)	25.6 (1)	25.51 (9)

**Table 2:** Polymerization results ^a^

Entry	Catalyst	(hexyl)_2_Zn (µmol)	Temperature *^b^* (°C)	Yield (g)	*F*_C3_ *^c^*	Expected *M*_n_ *^d^* (kDa)	Measured *M*_n_ *^e^* (kDa)	*M*_w_/*M*_n_
1	**I** (2-iPrPh; -iPr)	150	80−95−61	8.5	0.20	28	27	1.8
2	**19** (2-iPrPh; -C_7_H_13_)	150	80−90−65	10.2	0.20	33	28	1.9
3	**20** (2-iPrPh; -C_6_H_11_)	150	80−90−65	10.0	0.20	33	33	1.7
4	**21** (2-iPrPh; -C_5_H_9_)	150	80−93−63	8.1	0.18	27	25	1.4
5	**22** (2-iPrPh; -(3-pentyl))	150	80−88−59	5.6	0.18	19	18	1.8
6	**23** (2-iPrPh; -Et)	150	80−91−59	5.8	0.18	19	19	1.4
7	**24** (2-iPrPh; -Ph)	150		~0	−	−	−	−
8	**33** (Ph; -iPr)	150	80−92−57	5.3	0.16	18	19	1.4
9	**34** (Ph; -C_7_H_13_)	150	80−90−59	7.0	0.14	23	22	1.6
10	**35** (Ph; -C_6_H_11_)	150	80−89−57	5.5	0.16	18	19	1.5
11	**36** (Ph; -C_5_H_9_)	150	80−92−59	6.2	0.16	21	19	1.4
12	**37** (Ph; -Et)	150	80−89−57	3.9	0.14	13	14	1.5
13	**20** (2-iPrPh; -C_6_H_11_)	300	80−91−63	8.5	0.19	14	12	1.9
14	**20** (2-iPrPh; -C_6_H_11_)	450	80−90−60	9.8	0.19	11	9.0	1.8

*^a^* Polymerization conditions: An Hf complex (1.0 µmol) activated with [(C_18_H_37_)_2_N(H)Me]^+^[B(C_6_F_5_)_4_]^−^ (1.0 µmol), modified methylaluminoxane (50 µmol) as a scavenger, methylcyclohexane (26 g), a mixture of gases ethylene and propylene (a 1.0:1.5 molar ratio, 25 bar), 70 min. *^b^* Intitial, maximum reached spontaneously within 1 min by exotherm, and final values (heat not given externally). *^c^* The propylene mole fraction in the copolymer measured by means of ^1^H-NMR spectrum. *^d^* Calculated as yield (g)/(2 × Zn (mol)). *^e^* Measured by GPC at 160 °C using trichlorobenzene with PS standards, whose data values via universal calibration.

## Supplementary Materials

The following are available online at https://www.mdpi.com/2073-4360/12/5/1100/s1. Experimental details, characterization data, and Figures S1–S41 (^1^H and ^13^C NMR spectra of **1**–**37**, 2,6-dicycloheptylaniline, 2,6-dicyclohexylaniline, 2,6-dicyclopentylaniline and 2,6-di(3-pentyl)aniline).
