# Supplementary material for: Preparation of Pyridylamido Hafnium Complexes for Coordinative Chain Transfer Polymerization
Source: Polymers (Basel). 2020 May 11;12(5):1100. doi: 10.3390/polym12051100 (PMC7285347; doi:10.3390/polym12051100)
Supplement: Supplementary file 1 [file polymers-12-01100-s001.pdf]

Supporting Information: Preparation of pyridylamido hafnium complexes for coordinative chain transfer polymerization

Kyung Lee Park, [1] Jun Won Baek, [1] Seung Hyun Moon, [1] Sung Moon Bae, [1] Jong Chul Lee, [1] Junseong Lee,[2] Myong Sun Jeong, [3] and Bun Yeoul Lee[1]*

[1]Department of Molecular Science and Technology, Ajou University, Suwon 443-749, Korea.

[2]Department of Chemistry, Chonnam National University, Gwangju 500-757, South Korea.

[3]Intellectual Property Education Center, Anyang University, Anyang, 708-113, Korea.

* Correspondence: bunyeoul@ajou.ac.kr; Tel: 82-31-219-1844

2,6-Dicyclohexylaniline. It was prepared by the same procedure and conditions as that employed for 2,6-dicyclohexylaniline using 2,6-dibromoaniline (1.76 g, 7.00 mmol) and Pd-PEPPSI-Ihep^{Cl} catalyst (32.5 mg, 0.0350 mmol), and (cyclohexyl)ZnBr·1.4(THF) (5.66 g, 16.8 mmol) that was prepared with Zn dust and bromocyclohexane. Clear oil was obtained (1.13 g, 63%). ^{1}H NMR (C_6D_6): δ 7.08 (d, *J* = 7.2 Hz, 2H), 6.95 (t, *J* = 7.2 Hz, 1H), 3.29 (s, 2H, NH_2), 2.40 (m, 2H, CH), 1.90 (d, *J* = 13.2 Hz, 4H), 1.75 (m, 4H), 1.66 (d, *J* = 13.2 Hz, 2H), 1.31 (m, 8H), 1.18 (m, 2H) ppm. ^{13}C NMR (C_6D_6): δ 26.78, 27.60, 33.34, 39.30, 119.07, 123.76, 131.67, 140.78 ppm. HRMS(EI): *m/z* calcd ([M$^+$] $C_{18}H_{27}N$) 257.2143. Found: 257.2146.

2,6-Dicyclopentylaniline. It was prepared by the same procedure and conditions as that employed for 2,6-dicyclopentylaniline using 2,6-dibromoaniline (3.98 g, 15.8 mmol) and Pd-PEPPSI-Ihep^{Cl} catalyst (73.5 mg, 0.0792 mmol), and (cyclopentyl)ZnBr·2.3(THF) (14.3 g, 38.0 mol) that was prepared with Zn dust and bromocyclopentane. Clear oil was obtained (2.34 g, 64%). ^{1}H NMR (C_6D_6): δ 7.08 (d, *J* = 7.8 Hz, 2H), 6.95 (t, *J* = 7.8 Hz, 1H), 3.33 (s, 2H, NH_2), 2.82 (quintet, 2H, CH), 1.90 (m, 4H), 1.68 (m, 4H), 1.56 (m, 8H) ppm. ^{13}C NMR (C_6D_6): δ 25.47, 32.53, 40.63, 118.49, 123.77, 129.91, 142.50 ppm.

2,6-Di(3-pentyl)aniline. It was prepared by the same procedure and conditions as that employed for 2,6-di(pentan-3-yl)aniline using 2,6-dibromoaniline (5.24 g, 20.9 mmol) and Pd-PEPPSI-Ihep^{Cl}

catalyst (96.8 mg, 0.104 mmol), and (3-pentyl)ZnBr·1.0(THF) (14.5 g, 50.1 mol) that was prepared with Zn dust and 3-bromopentane. Clear oil was obtained (3.62 g, 74%). ^{1}H NMR (C_6D_6): δ 6.91 (m, 3H), 3.26 (s, 2H, NH_2), 2.33 (quintet, 2H, CH), 1.58 (m, 8H, CH_2), 0.81 (t, J = 7.2 Hz, 12H) ppm. ^{13}C NMR (C_6D_6): δ 12.19, 28.38, 42.53, 118.90, 124.34, 129.63, 143.35 ppm.

Compound 2. It was prepared by the same procedure and conditions as that employed for **1** using 2,6-dicyclohexylaniline (0.772 g, 3.00 mmol), 6-bromo-2-pyridinecarboxaldehyde (0.558 g, 3.00 mmol), and toluene (5 mL). Yellow solid was obtained (1.07 g, 84%). ^{1}H NMR (C_6D_6): δ 8.41 (s, 1H, NCH), 8.09 (d, J = 7.8 Hz, 1H), 7.53 (m, 3H), 6.85 (d, J = 7.8 Hz, 1H), 6.63 (t, J = 7.8 Hz, 1H), 2.74 (m, 2H), 1.87 (d, J = 12 Hz, 4H), 1.64 (d, J = 12.6 Hz, 4H), 1.54 (d, J = 10.8 Hz, 2H), 1.39 (quartet, J = 10.2 Hz, 4H), 1.11 (m, 6H) ppm. ^{13}C NMR (C_6D_6): δ 26.55, 27.33, 34.25, 39.30, 119.42, 124.32, 125.21, 129.83, 136.68, 138.82, 142.54, 148.94, 155.95, 162.06 ppm. HRMS(EI): *m/z* calcd ([M^+] $C_{24}H_{29}BrN_2$) 424.1514. Found: 424.1516.

Compound 3. It was prepared by the same procedure and conditions as that employed for **1** using 2,6-dicyclopentylaniline (1.69 g, 7.36 mmol), 6-bromo-2-pyridinecarboxaldehyde (1.37 g, 7.36 mmol), and toluene (12 mL). Yellow solid was obtained (1.07 g, 84%). An analytically pure compound was obtained through recrystallization in hexane and toluene at –30 °C. ^{1}H NMR (C_6D_6): δ 8.36 (s, 1H, NCH), 8.03 (d, J = 7.8 Hz, 1H), 7.13 (m, 3H), 6.84 (d, J = 7.8 Hz, 1H), 6.60 (t, J = 7.2 Hz, 1H), 3.13 (m, 2H), 1.91 (m, 4H), 1.59 (m, 8H), 1.40 (m, 4H) ppm. ^{13}C NMR (C_6D_6): δ 26.05, 34.72, 40.82, 119.55, 124.11, 125.21, 129.78, 135.28, 138.79, 142.45, 150.38, 155.90, 162.32 ppm. HRMS(EI): *m/z* calcd ([M^+] $C_{22}H_{25}BrN_2$) 396.1201. Found: 396.1203.

Compound 4. It was prepared by the same procedure and conditions as that employed for **1** using 2,6-di(3-pentyl)aniline (2.2 g, 9.42 mmol), 6-bromo-2-pyridinecarboxaldehyde (1.75 g, 9.42 mmol), and toluene (15 mL). Yellow solid was obtained (3.30 g, 87%). ^{1}H NMR (C_6D_6): δ 8.42 (s, 1H, NCH), 8.03 (d, J = 7.2 Hz, 1H), 7.11 (m, 1H), 7.03 (d, J = 7.8 Hz, 2H), 6.89 (d, J = 8.4 Hz, 1H), 6.66 (t, J = 8.4 Hz, 1H), 2.66 (quintet, J = 7.8 Hz, 2H), 1.54 (quintet, J = 7.2 Hz, 8H, CH_2), 0.80 (t, J = 7.2 Hz, 12H, CH_3) ppm. ^{13}C NMR (C_6D_6): δ 12.37, 29.61, 42.74, 119.42, 124.35, 125.20, 129.79, 134.12,

138.81, 142.50, 151.86, 155.93, 162.63 ppm. HRMS(EI): *m/z* calcd ([M^+] $C_{22}H_{29}BrN_2$) 400.1514. Found: 400.1512.

Compound 5. It was prepared by the same procedure and conditions as that employed for **1** using 2,6-diethylaniline (1.50 g, 10.1 mmol), 6-bromo-2-pyridinecarboxaldehyde (1.87 g, 10.1 mmol), and toluene (15 mL). Light Yellow solid was obtained (2.51 g, 79 %). ^{1}H NMR (C_6D_6): δ 8.23 (s, 1H, NCH), 7.96 (d, *J* = 7.2 Hz, 1H), 7.01 (s, 3H), 6.87 (d, *J* = 7.8 Hz, 1H), 6.62 (t, *J* = 8.4 Hz, 1H), 2.44 (quartet, *J* = 7.2 Hz, 4H, CH_2), 1.06 (t, *J* = 7.8 Hz, 6H, CH_3), ppm. ^{13}C NMR (C_6D_6): δ 14.88, 25.13, 119.46, 125.02, 126.84, 129.70, 132.90, 138.73, 142.29, 149.89, 155.89, 162.16 ppm. HRMS(EI): *m/z* calcd ([M^+] $C_{16}H_{17}BrN_2$) 316.0575. Found: 316.0574.

Compound 6. It was prepared by the same procedure and conditions as that employed for **1** using 2,6-diphenylaniline (2.00 g, 8.15 mmol), 6-bromo-2-pyridinecarboxaldehyde (1.52 g, 8.15 mmol), and toluene (10 mL). ^{1}H NMR (C_6D_6): δ 8.09 (s, 1H, NCH), 7.63 (d, *J* = 7.8 Hz, 1H), 7.38 (d, *J* = 7.2 Hz, 4H), 7.29 (d, *J* = 7.8 Hz, 2H), 7.07 (m, 5H), 6.97 (t, *J* = 7.8 Hz, 2H), 6.64 (d, *J* = 7.2 Hz, 1H), 6.42 (t, *J* = 7.2 Hz, 1H) ppm. ^{13}C NMR (C_6D_6): δ 119.44, 125.53, 127.06, 128.34, 129.47, 130.42, 130.46, 133.86, 138.36, 140.34, 142.02, 148.13, 155.59, 164.42 ppm. HRMS(EI): *m/z* calcd ([M^+] $C_{24}H_{17}BrN_2$) 412.0575. Found: 412.0572.

Compound 8. It was prepared by the same procedure and conditions as that employed for **7** using **2** (1.07 g, 2.51 mmol), 1-naphthylboronic acid (0.453 g, 2.64 mmol), Na_2CO_3 (0.700 g, 6.60 mmol), toluene (5 mL), degassed H_2O/EtOH (1 mL, *v/v*, 1:1), and a solution of $(Ph_3P)_4Pd$ (7.83 mg, 0.00678 mmol) in toluene (1 mL). Column chromatography on silica gel using hexane and ethyl acetate containing small quantity of triethylamine (*v/v*, 90:3:1) gave light yellow oil (0.712 g, 60%). ^{1}H NMR (C_6D_6): δ 8.70 (s, 1H, NCH), 8.41 (d, *J* = 7.8 Hz, 1H), 8.31 (d, *J* = 7.8 Hz, 1H), 7.68 (d, *J* = 7.2 Hz, 1H), 7.65 (d, *J* = 7.8 Hz, 1H), 7.54 (d, *J* = 7.2 Hz, 1H), 7.27 (m, 4H), 7.20 (m, 4H), 2.93 (m, 2H), 1.90 (d, *J* = 12 Hz, 4H), 1.61 (d, *J* = 13.2 Hz, 4H), 1.50 (d, *J* = 12.6 Hz, 2H), 1.38 (m, 4H), 1.11 (m, 6H), ppm. ^{13}C NMR (C_6D_6): δ 26.63, 27.38, 34.35, 39.36, 119.21, 124.32, 124.98, 125.50, 126.15, 126.21, 126.64, 126.75, 128.15, 128.73, 129.38, 131.81, 134.52, 136.94, 137.14, 138.52, 149.48, 155.13, 159.79, 164.05 ppm. HRMS(EI): *m/z* calcd ([M^+] $C_{34}H_{36}N_2$) 472.2878. Found: 472.2878.

Compound 9. It was prepared by the same procedure and conditions as that employed for **7** using **3** (1.63 g, 4.09 mmol), 1-naphthylboronic acid (0.739 g, 4.30 mmol), Na_2CO_3 (1.14 g, 10.8 mmol), toluene (8 mL), degassed H_2O/EtOH (3.5 mL, *v/v*, 1:1) and, a solution of $(Ph_3P)_4Pd$ (12.8 mg, 0.0111 mmol) in toluene (1 mL). An analytically pure compound was obtained through recrystallization in hexane and toluene at –30 °C. Yellow solid was obtained (1.24 g, 68%). ^1H NMR (C_6D_6): δ 8.66 (s, 1H, NCH), 8.39 (d, J = 7.2 Hz, 1H), 8.28 (m, 1H), 7.68 (m, 1H), 7.65 (d, J = 8.4 Hz, 1H), 7.56 (d, J = 7.2 Hz, 1H), 7.24 (m, 6H), 7.17 (s, 1H), 7.14 (d, J = 8.4 Hz, 1H), 3.30 (quintet, d, J = 7.2 Hz, 2H), 2.00 (m, 4H), 1.64 (m, 8H), 1.44 (m, 4H) ppm. ^{13}C NMR (C_6D_6): δ 26.07, 34.78, 40.94, 119.31, 124.12, 124.98, 125.53, 126.16, 126.18, 126.65, 126.77, 128.74, 129.38, 131.79, 134.51, 135.47, 137.07, 138.57, 150.98, 155.14, 159.73, 164.32 ppm. HRMS(EI): *m/z* calcd ([M^+] $C_{32}H_{32}N_2$) 444.2565. Found: 444.2561.

Compound 10. It was prepared by the same procedure and conditions as that employed for **7** using **4** (1.06 g, 2.63 mmol), 1-naphthylboronic acid (0.475 g, 2.76 mmol), Na_2CO_3 (0.733 g, 6.92 mmol), toluene (5 mL), degassed H_2O/EtOH (2.3 mL, *v/v*, 1:1), and a solution of $(Ph_3P)_4Pd$ (8.21 mg, 0.00711 mmol) in toluene (1 mL). Yellow solid was obtained (1.00 g, 85%). ^1H NMR (C_6D_6): δ 8.71 (s, 1H, NCH), 8.37 (d, J = 7.2 Hz, 1H), 8.31 (m, 1H), 7.67 (t, J = 4.2 Hz, 1H), 7.65 (d, J = 8.4 Hz, 1H), 7.58 (d, J = 7.2 Hz, 1H), 7.28 (m, 4H), 7.21 (d, J = 7.8 Hz, 1H), 7.14 (d, J = 7.2 Hz, 1H) ,7.07 (d, J = 7.8 Hz, 2H), 2.84 (quintet, J = 6.6 Hz, 2H, CH), 1.60 (quintet, J = 7.8 Hz, 8H, CH_2), 0.867 (t, J = 7.2 Hz, 12H, CH_3) ppm. ^{13}C NMR (C_6D_6): δ 12.44, 29.69, 42.80, 119.18, 124.33, 124.96, 125.53, 126.17, 126.66, 126.75, 128.21, 128.73, 129.37, 131.79, 134.34, 134.51, 137.05, 138.58, 152.45, 155.10, 159.75, 164.66 ppm. HRMS(EI): *m/z* calcd ([M^+] $C_{32}H_{36}N_2$) 448.2878. Found: 448.2881.

Compound 11. It was prepared by the same procedure and conditions as that employed for **7** using **5** (2.34 g, 7.02 mmol), 1-naphthylboronic acid (1.27 g, 7.37 mmol), Na_2CO_3 (1.96 g, 18.5 mmol), toluene (10 mL), degassed H_2O/EtOH (6.5 mL, *v/v*, 1:1) and a solution of $(Ph_3P)_4Pd$ (21.9 mg, 0.0190 mmol) in toluene (2 mL). An analytically pure compound was obtained through recrystallization in hexane and toluene at –30 °C. Yellow solid was obtained (2.35 g, 92%). ^1H NMR (C_6D_6): δ 8.56 (s, 1H, NCH), 8.33 (d, J = 7.2 Hz, 1H), 8.29 (m, 1H), 7.69 (t, J = 2.4 Hz, 1H), 7.67 (d, J = 8.4 Hz, 1H), 7.58

(d, J = 6.6 Hz, 1H), 7.26 (m, 5H), 7.04 (m, 3H), 2.56 (quartet, J = 7.2 Hz, 4H, CH_2), 1.14 (t, J = 7.2 Hz, 6H, CH_3) ppm. ^{13}C NMR (C_6D_6): δ 14.93, 25.26, 119.24, 124.76, 125.54, 126.17, 126.19, 126.69, 126.83, 128.17, 128.76, 129.37, 131.80, 133.06, 134.53, 136.97, 138.69, 150.49, 155.13, 159.66, 164.12 ppm. HRMS(EI): *m/z* calcd ([M$^+$] $C_{26}H_{24}N_2$) 364.1939. Found: 364.1938.

Compound 12. It was prepared by the same procedure and conditions as that employed for **7** using **6** (2.08 g, 5.04 mmol), 1-naphthylboronic acid (0.910 g, 5.29 mmol), Na_2CO_3 (1.40 g, 13.3 mmol), toluene (8 mL), degassed H_2O/EtOH (4.6 mL, *v/v*, 1:1), and a solution of $(Ph_3P)_4Pd$ (15.7 mg, 0.0136 mmol) in toluene (2 mL). Yellow solid was obtained (2.08 g, 90%). ^{1}H NMR (C_6D_6): δ 8.35 (s, 1H, NCH), 8.04 (m, 1H), 7.98 (d, J = 7.8 Hz, 1H), 7.65 (m, 1H), 7.60 (d, J = 7.8 Hz, 1H), 7.49 (d, J = 7.8 Hz, 4H), 7.34 (d, J = 7.8 Hz, 2H), 7.32 (d, J = 7.2 Hz, 1H), 7.28 (m, 2H), 7.20 (t, J = 7.8 Hz, 1H), 7.08 (m, 9H) ppm. ^{13}C NMR (C_6D_6): δ 119.22, 125.37, 126.01, 126.38, 126.56, 126.94, 128.33, 128.58, 129.21, 130.50, 130.60, 131.71, 134.10, 134.44, 136.75, 138.41, 140.65, 148.55, 154.69, 159.29, 166.55 ppm. HRMS(EI): *m/z* calcd ([M$^+$] $C_{34}H_{24}N_2$) 460.1939. Found: 460.1938.

Compound 14. It was prepared by the same procedure and conditions as that employed for **13** using **8** (0.247, 0.523 mmol) and 2-isopropylphenyllithium (0.114 g, 0.904 mmol). Yellow solid was obtained (0.257 g, 83%). ^{1}H NMR (C_6D_6): δ 8.24 (m, 1H), 7.90 (m, 1H), 7.64 (m, 1H), 7.62 (d, J = 7.8 Hz, 1H), 7.56 (d, J = 7.2 Hz, 1H), 7.26 (m, 3H), 7.22 (m, 4H), 7.11 (m, 5H), 5.62 (d, J = 5.4 Hz, 1H, NCH), 4.59 (d, J = 5.4 Hz, 1H, NH), 3.31 (septet, J = 7.2 Hz, 1H,CH), 2.74 (m, 2H), 1.79 (d, J = 7.8 Hz, 2H), 1.64 (m, 4H), 1.54 (m, 4H), 1.32 (m, 4H), 1.08 (m, 2H), 1.03 (d, J = 6.6 Hz, 3H, CH_3), 1.00 (m, 1H), 0.980 (d, J = 6.6 Hz, 3H, CH_3), 0.921 (m, 3H) ppm. ^{13}C NMR (C_6D_6): δ 23.78, 24.45, 26.63, 27.42, 27.54, 28.96, 34.77, 35.08, 39.01, 67.64, 119.99, 122.89, 124.13, 124.80, 125.36, 125.77, 126.08, 126.46, 126.56, 126.71, 127.58, 128.55, 129.35, 131.84, 134.64, 136.94, 138.77, 141.88, 142.24, 144.97, 146.32, 159.28, 163.74 ppm. HRMS(EI): *m/z* calcd ([M$^+$] $C_{43}H_{48}N_2$) 592.3817. Found: 592.3819.

Compound 15. It was prepared by the same procedure and conditions as that employed for **13** using **9** (1.20 g, 2.70 mmol) and 2-isopropylphenyllithium (0.589 g, 4.66 mmol). Light yellow solid was obtained (1.38 g, 91%). ^{1}H NMR (C_6D_6): δ 8.22 (m, 1H), 7.77 (d, J = 8.4 Hz, 1H), 7.65 (m, 1H), 7.61

(d, J = 8.4 Hz, 1H), 7.53 (d, J = 7.2 Hz, 1H), 7.27 (m, 3H), 7.19 (m, 1H), 7.13 (m, 6H), 7.07 (d, J = 7.8 Hz, 1H), 7.03 (d, J = 7.2 Hz, 1H), 5.74 (s, 1H, NCH), 4.95 (s, 1H, NH), 3.34 (septet, J = 7.2 Hz, 1H, CH), 3.26 (m, 2H), 1.87 (m, 2H), 1.71 (m, 2H), 1.61 (m, 4H), 1.48 (m, 4H), 1.32 (m, 4H), 1.03 (d, J = 6.6 Hz, 3H, CH_3), 0.981 (d, J = 6.6 Hz, 3H, CH_3) ppm. ^{13}C N-MR (C_6D_6): δ 23.83, 24.39, 26.06 26.08, 28.94, 35.54, 35.62, 40.58, 67.19, 120.19, 122.87, 124.25, 124.69, 125.39, 125.89, 126.10, 126.30, 126.58, 126.60, 127.65, 128.59, 128.63, 129.31, 131.86, 134.60, 136.86, 138.87, 140.76, 141.40, 145.93, 146.72, 159.08, 163.23 ppm. HRMS(EI): *m/z* calcd ([M$^+$] $C_{41}H_{44}N_2$) 564.3504. Found: 564.3507.

Compound 16. It was prepared by the same procedure and conditions as that employed for **13** using **10** (0.763 g, 1.70 mmol) and 2-isopropylphenyllithium (0.371 g, 2.94 mmol). Light yellow solid was obtained (0.878 g, 91%). ^{1}H NMR (C_6D_6): δ 8.28 (m, 1H), 7.70 (d, J = 7.8 Hz, 1H), 7.64 (m, 1H), 7.61 (d, J = 8.4 Hz, 1H), 7.52 (d, J = 7.8 Hz, 1H), 7.27 (m, 3H), 7.18 (m, 4H), 7.11 (m, 3H), 7.01 (d, J = 7.8 Hz, 2H), 5.72 (d, J = 7.8 Hz, 1H, NCH), 4.83 (d, J = 7.8 Hz, 1H, NH), 3.37 (septet, J = 6.6 Hz, 1H, CH), 2.81 (m, 2H), 1.60 (d, J = 7.2 Hz, 2H, CH_2), 1.54 (d, J = 7.2 Hz, 2H, CH_2), 1.41 (d, J = 7.2 Hz, 2H, CH_2), 1.32 (d, J = 7.2 Hz, 2H, CH_2), 1.01 (d, J = 6.6 Hz, 3H, CH_3), 0.940 (d, J = 6.6 Hz, 3H, CH_3), 0.793 (t, J = 6.6 Hz, 6H, CH_3), 0.700 (t, J = 6.6 Hz, 6H, CH_3) ppm. ^{13}C NMR (C_6D_6): δ 12.22, 12.41, 23.80, 24.42, 28.98, 29.41, 29.72, 42.01, 67.19, 120.14, 122.83, 124.31, 125.32, 126.04, 126.42, 126.59, 126.72, 127.67, 128.55, 128.74, 129.36, 131.89, 134.63, 136.96, 138.74, 140.66, 141.51, 146.78, 146.85, 159.04, 163.40 ppm. HRMS(EI): *m/z* calcd ([M$^+$] $C_{41}H_{48}N_2$) 568.3817. Found: 568.3820.

Compound 17. It was prepared by the same procedure and conditions as that employed for **13** using **11** (0.400 g, 1.10 mmol) and 2-isopropylphenyllithium (0.239 g, 1.90 mmol). Light yellow solid was obtained (0.432 g, 82%). ^{1}H NMR (C_6D_6): δ 8.18 (m, 1H), 7.79 (m, 1H), 7.66 (m, 1H), 7.64 (d, J = 7.8 Hz, 1H), 7.50 (d, J = 7.8 Hz, 1H), 7.27 (m, 3H), 7.18 (m, 2H), 7.14 (m, 2H), 7.01 (m, 5H), 5.83 (s, 1H, NCH), 4.78 (s, 1H, NH), 3.33 (septet, J = 6.6 Hz, 1H, CH), 2.50 (m, 4H, CH_2), 1.02 (m, 12H, CH_3) ppm. ^{13}C NMR (C_6D_6): δ 14.95, 23.99, 24.84, 25.05, 28.93, 65.42, 120.00, 122.97, 123.18, 125.40, 125.86, 126.07, 126.37, 126.60, 126.62, 127.09, 127.69, 127.84, 128.58, 129.28, 131.88,

134.57, 136.90, 137.01, 138.95, 141.42, 145.44, 146.52, 159.21, 163.41 ppm. HRMS(EI): *m/z* calcd ([M^+] $C_{35}H_{36}N_2$) 484.2878. Found: 484.2876.

Compound 18. It was prepared by the same procedure and conditions as that employed for **13** using **12** (0.459 g, 0.996 mmol) and 2-isopropylphenyllithium (0.217 g, 1.72 mmol). White solid was obtained (0.428 g, 74%). ^{1}H NMR (C_6D_6): δ 8.05 (d, *J* = 8.4 Hz, 1H), 7.68 (d, *J* = 8.4 Hz, 1H), 7.64 (d, *J* = 8.4 Hz, 1H), 7.42 (d, *J* = 8.4 Hz, 4H), 7.27 (m, 5H), 7.14 (d, *J* = 7.2 Hz, 2H), 7.01 (m, 10H), 6.89 (m, 2H), 6.71 (d, *J* = 7.8 Hz, 1H), 5.82 (d, *J* = 10.8 Hz, 1H, NCH), 5.22 (d, *J* = 10.8 Hz, 1H, NH), 2.97 (septet, *J* = 6.0 Hz, 1H, CH), 1.01 (d, *J* = 6.0 Hz, 3H, CH_3), 0.872 (d, *J* = 6.0 Hz, 3H, CH_3) ppm. ^{13}C NMR (C_6D_6): δ 23.96, 24.65, 28.57, 61.56, 119.97, 120.91, 122.67, 125.36, 125.52, 125.94, 126.19, 126.48, 162.87, 127.11, 127.34, 127.71, 128.44, 128.67, 129.03, 129.71, 130.99, 131.84, 133.29, 134.51, 136.40, 138.97, 140.04, 141.43, 143.23, 146.44, 158.85, 162.62 ppm. HRMS(EI): *m/z* calcd ([M^+] $C_{43}H_{36}N_2$) 580.2878. Found: 580.2881.

Compound 27. It was prepared by the same procedure and conditions as that employed for **26** using **25** (0.609 g, 2.14 mmol), t-BuLi (2.52 mL, 1.7 M solution in hexane, 4.3 mmol), 2,6-(cycloheptyl)$_2C_6H_3N$=C(H)Ph (0.800 g, 2.14 mmol), and THF (16 mL). Purification by column chromatography on silica gel using hexane and toluene containing small quantity of triethylamine (*v/v*, 75:25:1) gave a light yellow solid (0.412 g, 63 %). ^{1}H NMR (C_6D_6): δ 8.27 (m, 1H), 7.67 (m, 2H), 7.60 (d, *J* = 7.2 Hz, 3H), 7.28 (m, 3H), 7.18 (m, 2H), 7.10 (m, 6H), 6.89 (m, 1H), 5.32 (d, *J* = 7.2 Hz, 1H, NCH), 5.18 (d, *J* = 7.8 Hz, 1H, NH), 3.04 (m, 2H), 1.81 (m, 4H), 1.55 (m, 8H), 1.37 (m, 8H), 1.18 (m, 4H) ppm. ^{13}C NMR (C_6D_6): δ 27.65, 27.84, 27.99, 37.20, 37.54, 40.48, 70.57, 120.30, 123.23, 124.06, 124.56, 125.34, 126.15, 126.59, 126.75, 127.17, 128.58, 128.63, 129.40, 131.86, 134.65, 137.10, 138.82, 142.71, 144.07, 144.87, 159.43, 162.82 ppm. HRMS(EI): *m/z* calcd ([M^+] $C_{42}H_{46}N_2$) 578.3661. Found: 578.3660.

Compound 28. It was prepared by the same procedure and conditions as that employed for **26** using **25** (0.156 g, 0.548 mmol), t-BuLi (0.65 mL, 1.7 M solution in hexane, 1.1 mmol), 2,6-(cyclohexyl)$_2C_6H_3N$=C(H)Ph (0.189 g, 0.548 mmol), and THF (7 mL). Purification by column chromatography on silica gel using hexane and toluene containing small quantity of triethylamine (*v/v*,

75:25:1) gave a light yellow solid (0.208 g, 69 %). ^{1}H NMR (C_6D_6): δ 8.30 (m, 1H), 7.67 (m, 2H), 7.61 (d, J = 7.8 Hz, 3H), 7.28 (m, 3H), 7.19 (m, 2H), 7.10 (m, 6H), 6.83 (quintet, J = 4.2 Hz, 1H), 5.31 (s, 1H, NCH), 5.24 (s, 1H, NH), 2.89 (m, 2H), 1.75 (d, J = 12.6 Hz, 2H), 1.70 (d, J = 12.6 Hz, 2H), 1.59 (d, J = 13.2 Hz, 2H), 1.55 (d, J = 12.6 Hz, 2H), 1.50 (d, J = 12.6 Hz, 2H), 1.32 (m, 4H), 1.09 (m, 2H), 0.922 (m, 4H) ppm. ^{13}C NMR (C_6D_6): δ 26.63, 27.18, 27.49, 34.75, 35.12, 38.98, 70.71, 120.32, 123.19, 123.81, 124.75, 125.38, 126.18, 126.59, 126.71, 127.19, 127.78, 128.60, 129.40, 131.85, 134.64, 137.11, 138.88, 141.90, 144.69, 144.94, 159.38, 162.73 ppm. HRMS(EI): *m/z* calcd ([M$^+$] $C_{40}H_{42}N_2$) 550.3348. Found: 550.3350.

Compound 29. It was prepared by the same procedure and conditions as that employed for **26** using **25** (0.493 g, 1.74 mmol), t-BuLi (2.0 mL, 1.7 M solution in hexane, 3.5 mmol), 2,6-(cyclopentyl)$_2C_6H_3$N=C(H)Ph (0.551 g, 1.74 mmol), and THF (20 mL). Purification by column chromatography on silica gel using hexane and toluene containing small quantity of triethylamine (*v/v*, 75:25:1) gave a light yellow solid (0.637 g, 70 %). ^{1}H NMR (C_6D_6): δ 8.24 (m, 1H), 7.67 (m, 2H), 7.63 (d, J = 7.2 Hz, 2H), 7.56 (d, J = 6.6 Hz, 1H), 7.31 (d, J = 8.4 Hz, 1H), 7.27 (m, 2H), 7.18 (m, 2H), 7.12 (d, J = 6.6 Hz, 2H), 7.05 (m, 4H), 6.75 (m, 1H), 5.60 (d, J = 7.2 Hz, 1H, NCH), 5.36 (d, J = 8.4 Hz, 1H, NH), 3.37 (m, 2H), 1.87 (m, 2H), 1.78 (m, 2H), 1.58 (m, 4H), 1.49 (m, 4H), 1.26 (m, 4H) ppm. ^{13}C NMR (C_6D_6): δ 25.93, 25.98, 35.46, 35.53, 40.64, 70.33, 120.66, 123.17, 123.59, 124.68, 125.42, 126.18, 126.53, 126.61, 127.21, 127.68, 127.83, 128.59, 128.63, 129.35, 131.79, 134.59, 136.94, 138.95, 139.70, 144.67, 146.13, 159.25, 162.51 ppm. HRMS(EI): *m/z* calcd ([M$^+$] $C_{38}H_{38}N_2$) 522.3035. Found: 522.3033.

Compound 30. It was prepared by the same procedure and conditions as that employed for **26** using **25** (0.906 g, 3.19 mmol), t-BuLi (3.8 mL, 1.7 M solution in hexane, 6.4 mmol), (2,6-(3-pentyl)$_2C_6H_3$N=C(H)Ph (1.02 g, 3.19 mmol), and THF (20 mL). Purification by column chromatography on silica gel using hexane and toluene containing small quantity of triethylamine (*v/v*, 75:25:1) gave a light yellow solid (1.08 g, 65 %). ^{1}H NMR (C_6D_6): δ 8.27 (d, J = 6.6 Hz, 1H), 7.66 (m, 2H), 7.57 (m, 3H), 7.29 (m, 3H), 7.01 (m, 8H), 6.89 (m, 1H), 5.37 (m, 1H, NCH), 5.33 (m, 1H, NH), 2.90 (m, 2H, CH), 1.55 (m, 4H, CH_2), 1.40 (m, 4H, CH_2), 0.73 (m, 12H, CH_3) ppm. ^{13}C NMR

(C_6D_6): δ 12.22, 12.30, 29.53, 29.72, 41.95, 70.29, 120.52, 123.23, 123.82, 124.27, 125.36, 126.12, 126.60, 126.63, 127.14, 127.83, 128.62, 128.65, 129.36, 131.87, 134.60, 136.97, 138.94, 139.88, 144.65, 147.10, 159.39, 162.70 ppm. HRMS(EI): *m/z* calcd ([M^+] $C_{38}H_{42}N_2$) 526.3348. Found: 526.3352.

Compound 31. It was prepared by the same procedure and conditions as that employed for **26** using **25** (0.220 g, 0.774 mmol), t-BuLi (0.91 mL, 1.7 M solution in hexane, 1.6 mmol), (2,6-$Et_2C_6H_3N$=C(H)Ph (0.184 g, 0.774 mmol), and THF (10 mL). Purification by column chromatography on silica gel using hexane and toluene containing small quantity of triethylamine (*v/v*, 75:25:1) gave a light yellow solid (0.210 g, 61 %). ^{1}H NMR (C_6D_6): δ 8.21 (m, 1H), 7.68 (m, 2H), 7.54 (d, *J* = 7.2 Hz, 1H), 7.51 (d, *J* = 8.4 Hz, 2H), 7.30 (t, *J* = 7.2 Hz, 1H), 7.26 (m, 2H), 7.11 (t, *J* = 7.8 Hz, 2H), 7.05 (m, 2H), 7.00 (m, 3H), 6.93 (m, 1H), 6.80 (m, 1H), 5.59 (d, *J* = 8.4 Hz, 1H, NCH), 5.52 (d, *J* = 8.4 Hz, 1H, NH), 2.62 (m, 4H, CH_2), 1.06 (t, *J* = 6.6 Hz, 6H, CH_3) ppm. ^{13}C NMR (C_6D_6): δ 14.82, 25.47, 68.09, 120.75, 122.56, 123.35, 125.48, 126.16, 126.50, 126.63, 127.09, 127.24, 127.73, 127.81, 128.60, 128.65, 129.28, 131.84, 134.54, 136.08, 136.94, 139.08, 144.24, 144.89, 159.17, 162.33 ppm. HRMS(EI): *m/z* calcd ([M^+] $C_{32}H_{30}N_2$) 442.2409. Found: 442.2408.

Compound 32. It was prepared by the same procedure and conditions as that employed for **26** using **25** (0.693 g, 2.44 mmol), t-BuLi (2.9 mL, 1.7 M solution in hexane, 4.9 mmol), 2,6-$Ph_2C_6H_3N$=C(H)Ph (0.815 g, 2.44 mmol), and THF (15 mL). Purification by column chromatography on silica gel using hexane and toluene containing small quantity of triethylamine (*v/v*, 75:25:1) gave a white solid (0.881 g, 67 %). ^{1}H NMR (C_6D_6): δ 7.95 (d, *J* = 8.4 Hz, 1H), 7.74 (d, *J* = 8.4 Hz, 1H), 7.72 (d, *J* = 8.4 Hz, 1H), 7.57 (d, *J* = 7.2 Hz, 4H), 7.31 (t, *J* = 8.4 Hz, 2H), 7.26 (d, *J* = 6.0 Hz, 1H), 7.21 (t, *J* = 6.6 Hz, 1H), 7.10 (d, *J* = 7.8 Hz, 2H), 7.04 (m, 6H), 6.97 (m, 4H), 6.89 (d, *J* = 7.8 Hz, 1H), 6.86 (d, *J* = 3.6 Hz, 2H), 6.78 (t, *J* = 7.2 Hz, 1H), 6.67 (d, *J* = 10.2 Hz, 1H), 6.49 (m, 1H), 5.25 (d, *J* = 10.2 Hz, 1H) ppm. ^{13}C NMR (C_6D_6): δ 64.91, 120.43, 120.77, 123.12, 125.44, 126.03, 126.41, 126.72, 127.02, 127.19, 128.43, 128.64, 128.86, 129.89, 130.80, 131.91, 133.42, 134.44, 136.68, 139.04, 141.84, 142.59, 143.27, 158.60, 160.96 ppm. HRMS(EI): *m/z* calcd ([M^+] $C_{40}H_{30}N_2$) 538.2409. Found: 538.2411.

Figure S1. ^{1}H and ^{13}C NMR spectra of 2,6-dicycloheptylaniline.

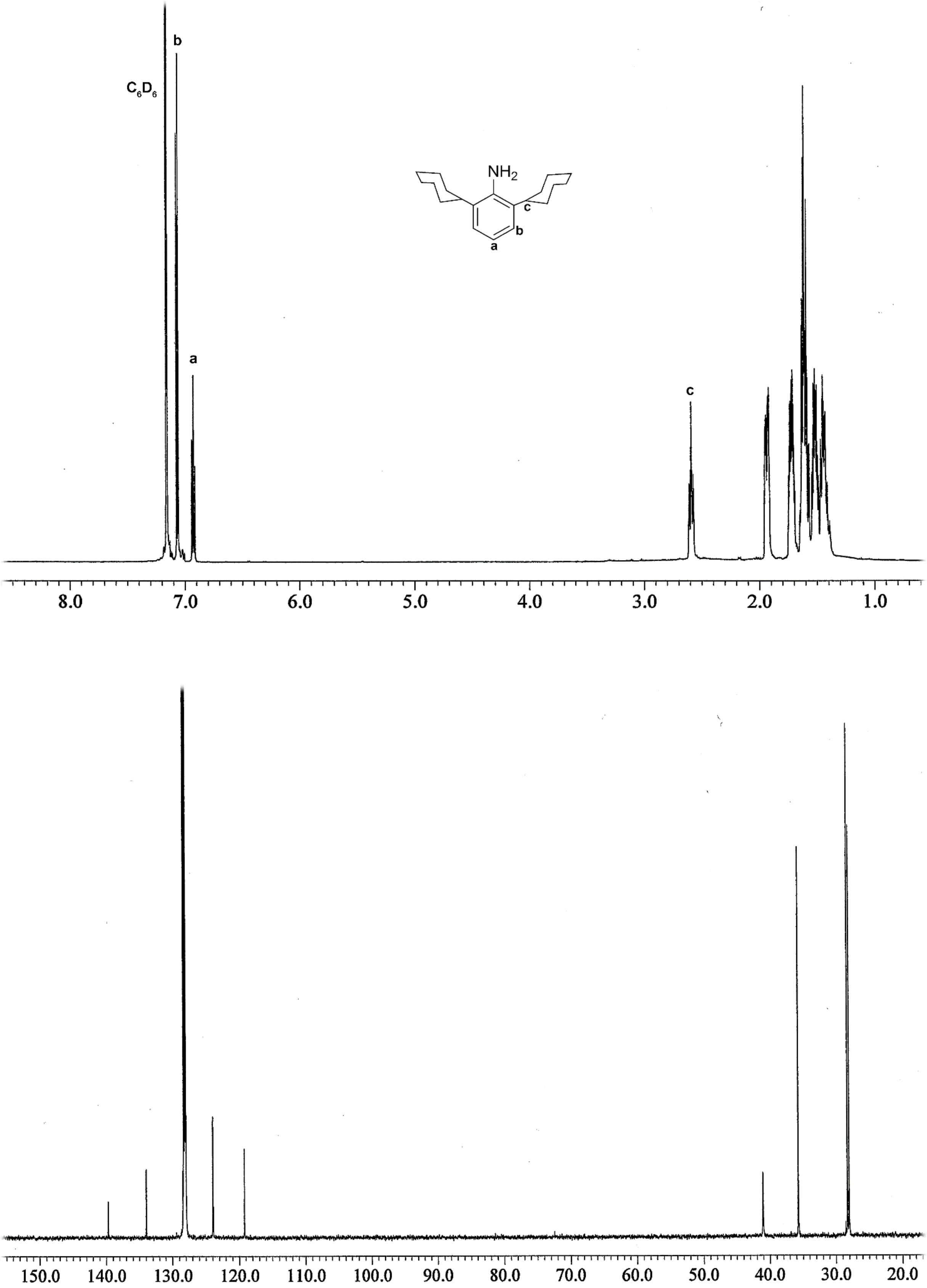

Figure S2. ^{1}H and ^{13}C NMR spectra of 2,6-dicyclohexylaniline.

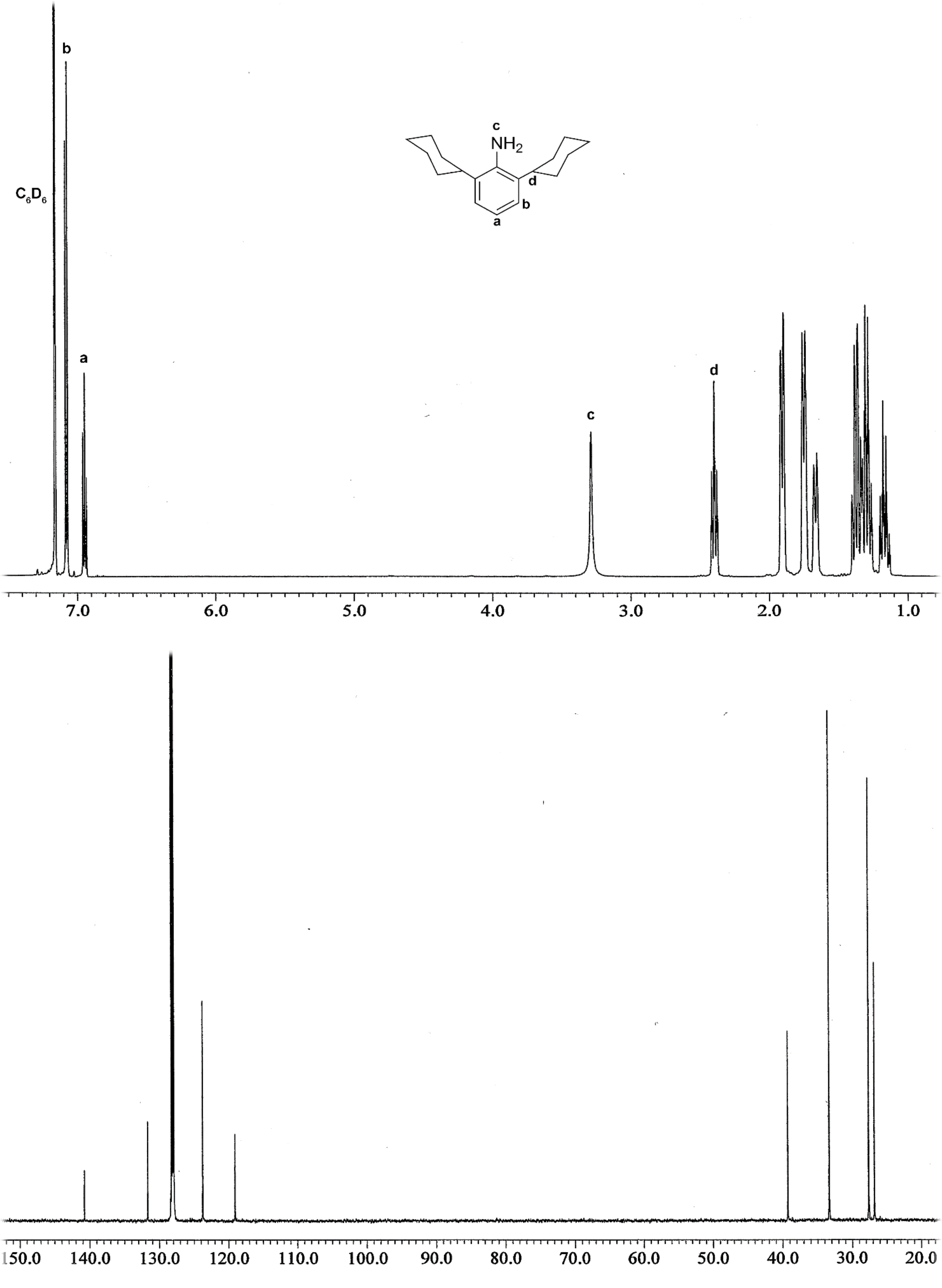

Figure S3. ^{1}H and ^{13}C NMR spectra of 2,6-dicyclopentylaniline.

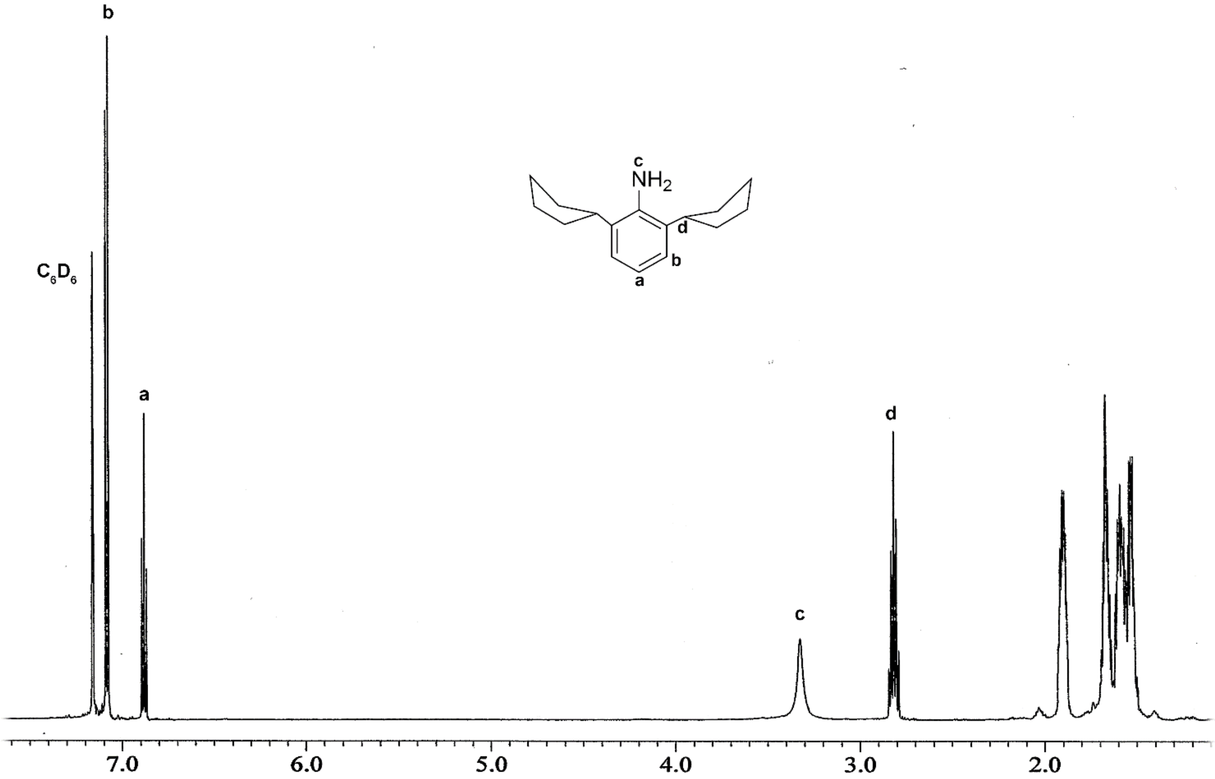

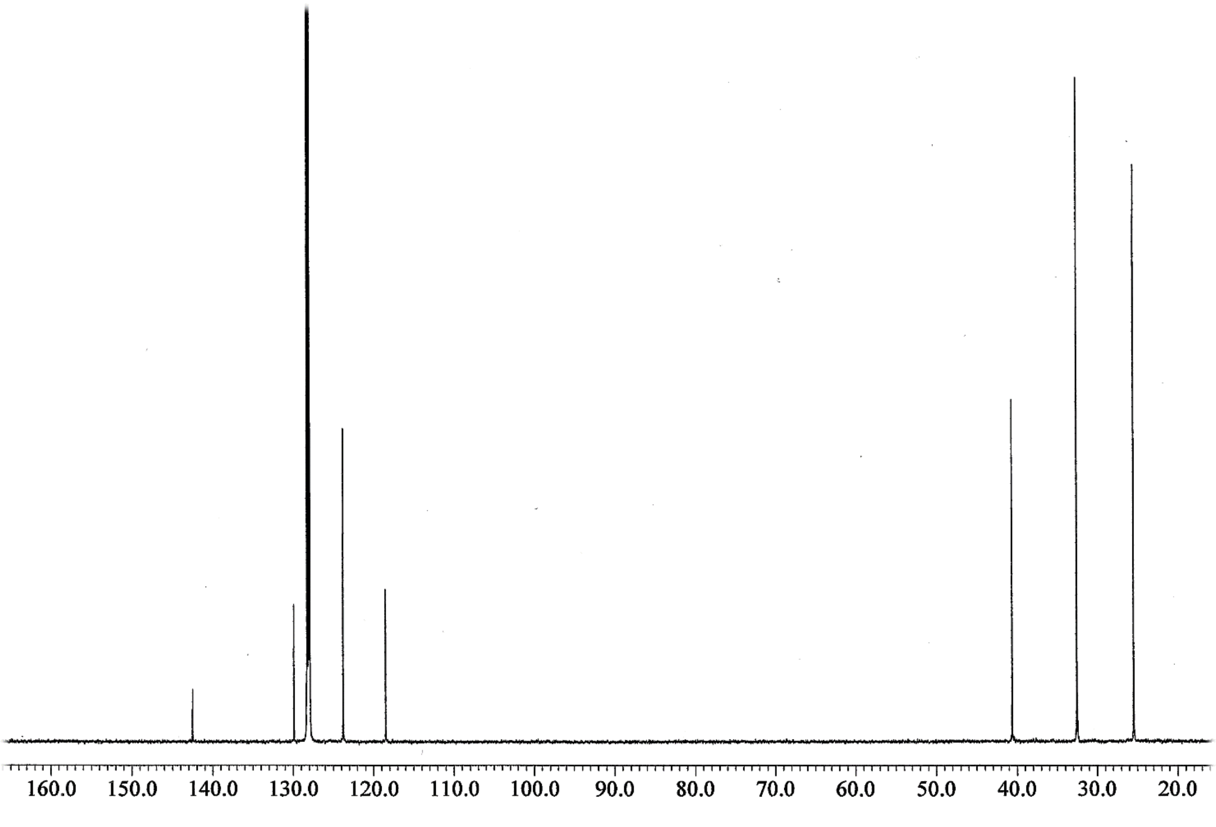

Figure S4. ^{1}H and ^{13}C NMR spectra of 2,6-di(3-pentyl)aniline.

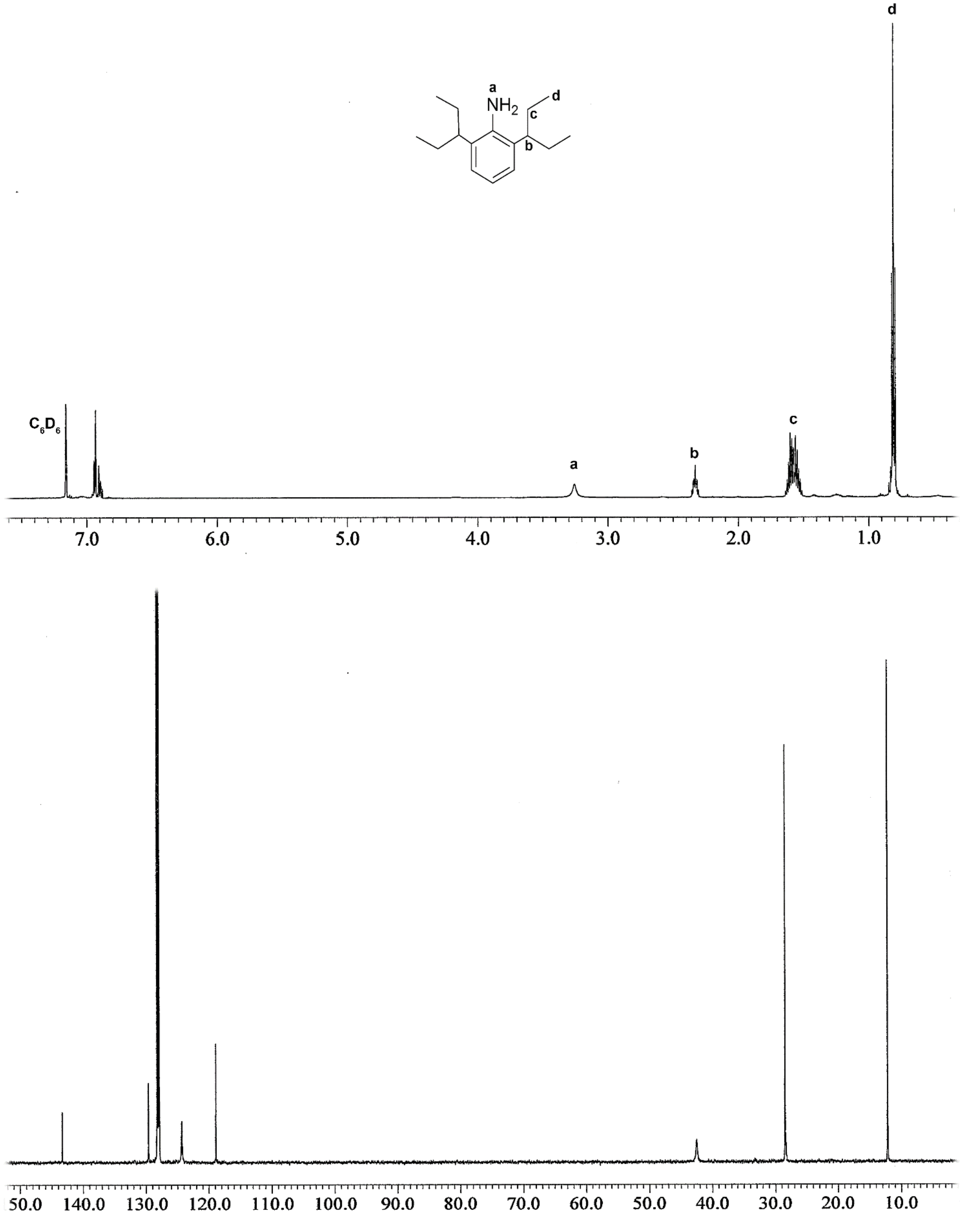

Figure S5. ^{1}H and ^{13}C NMR spectra of **1**.

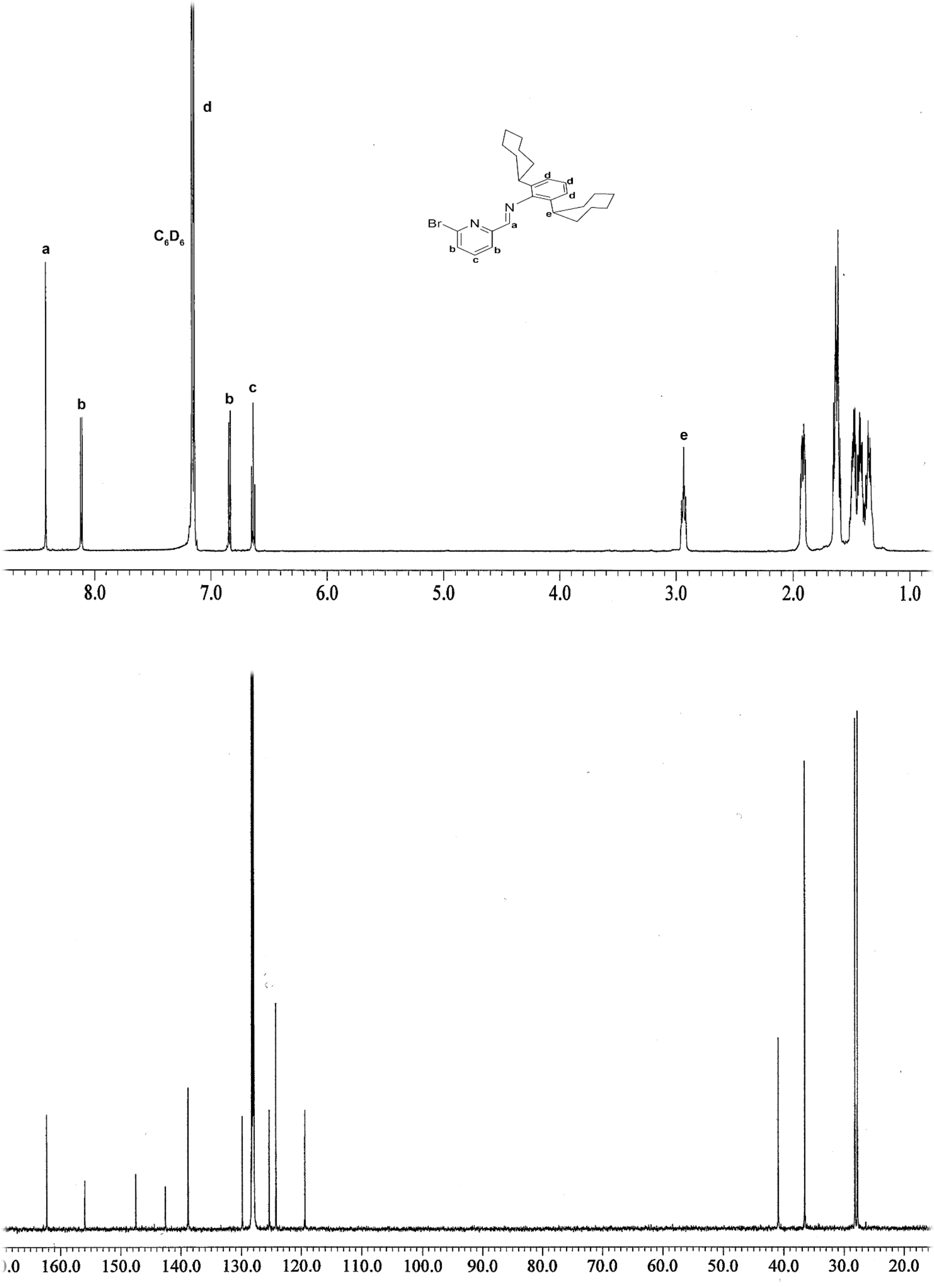

Figure S6. ^{1}H and ^{13}C NMR spectra of **2**.

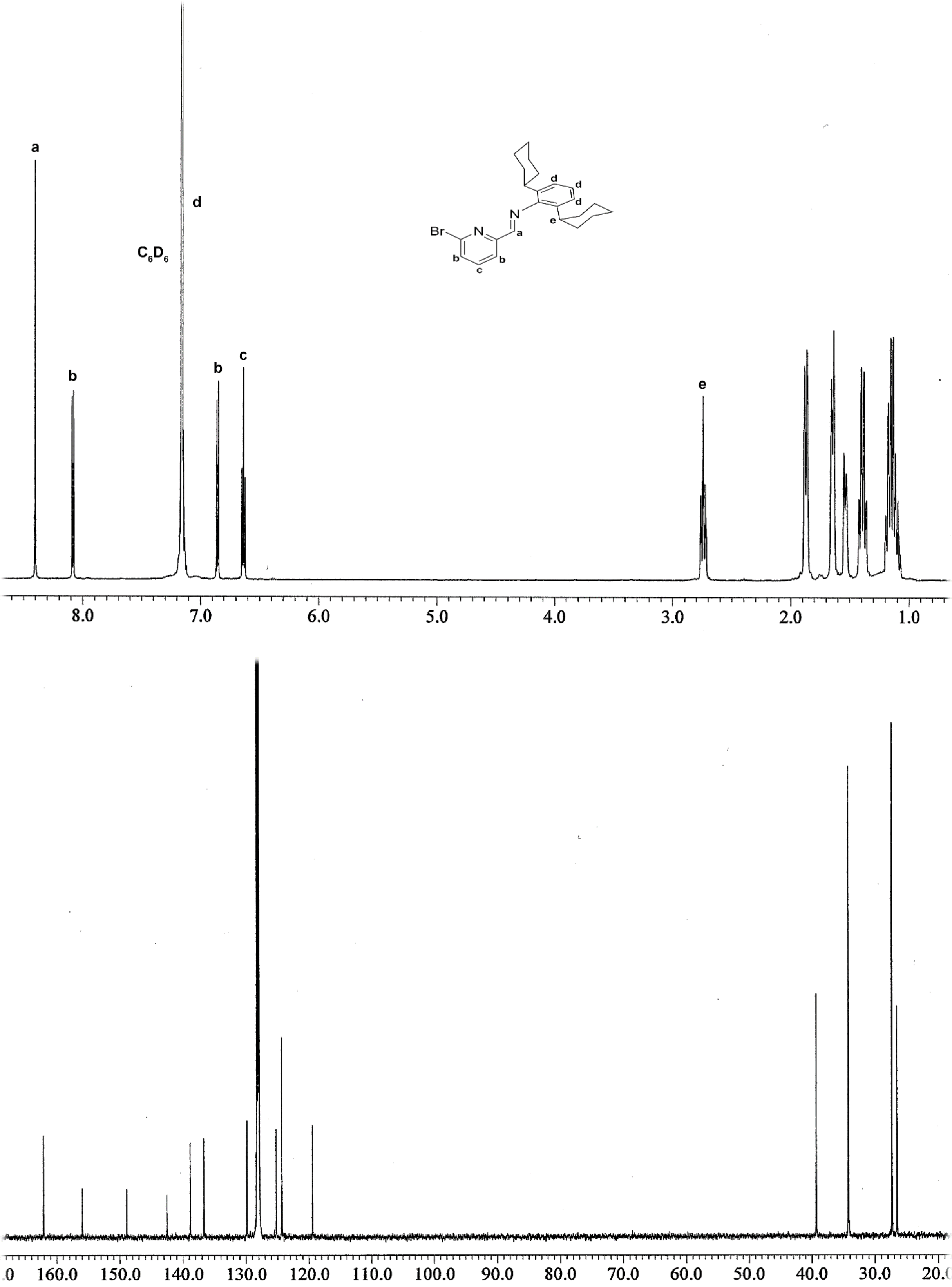

Figure S7. ^{1}H and ^{13}C NMR spectra of **3.**

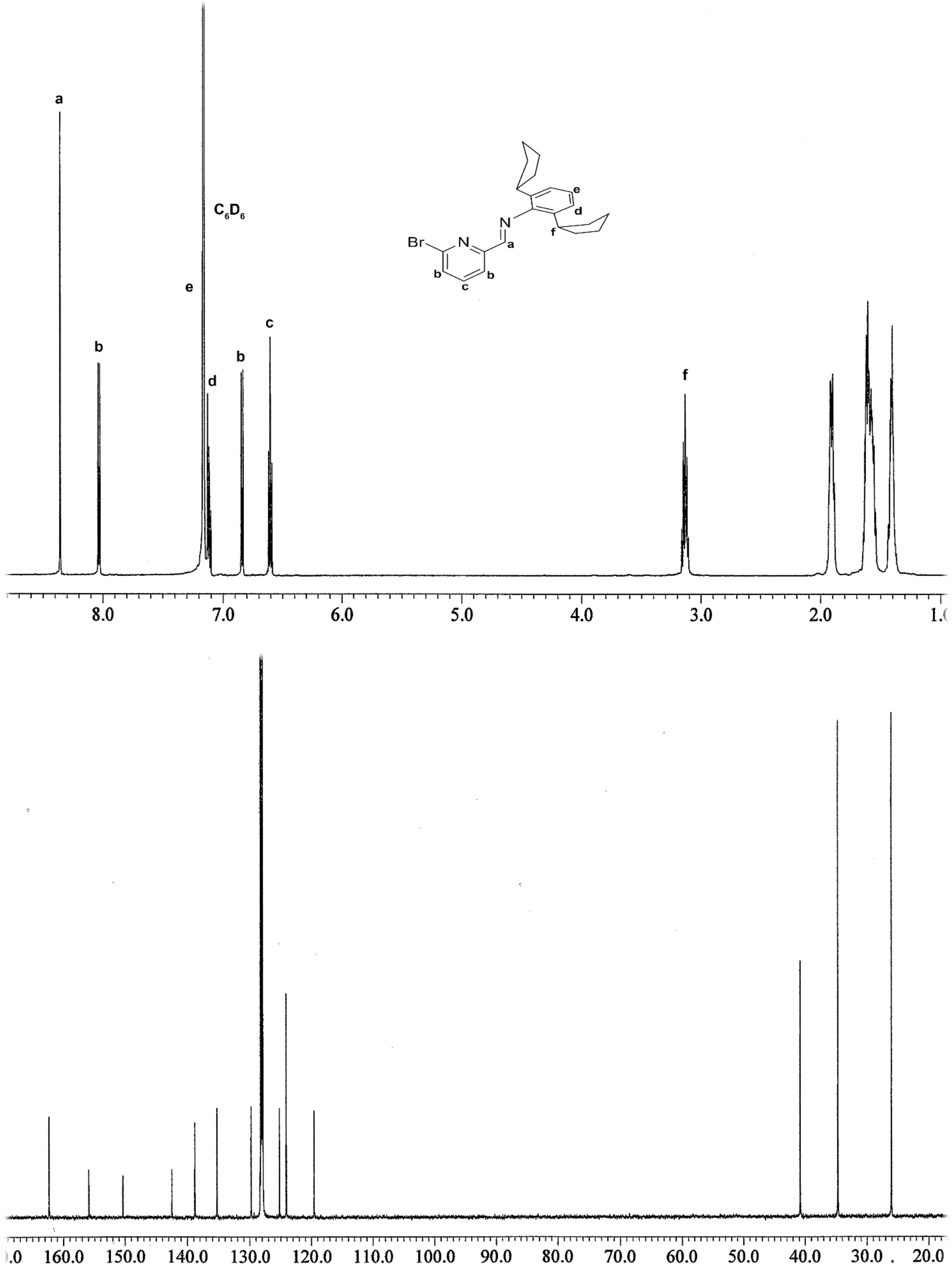

Figure S8. ^{1}H and ^{13}C NMR spectra of **4.**

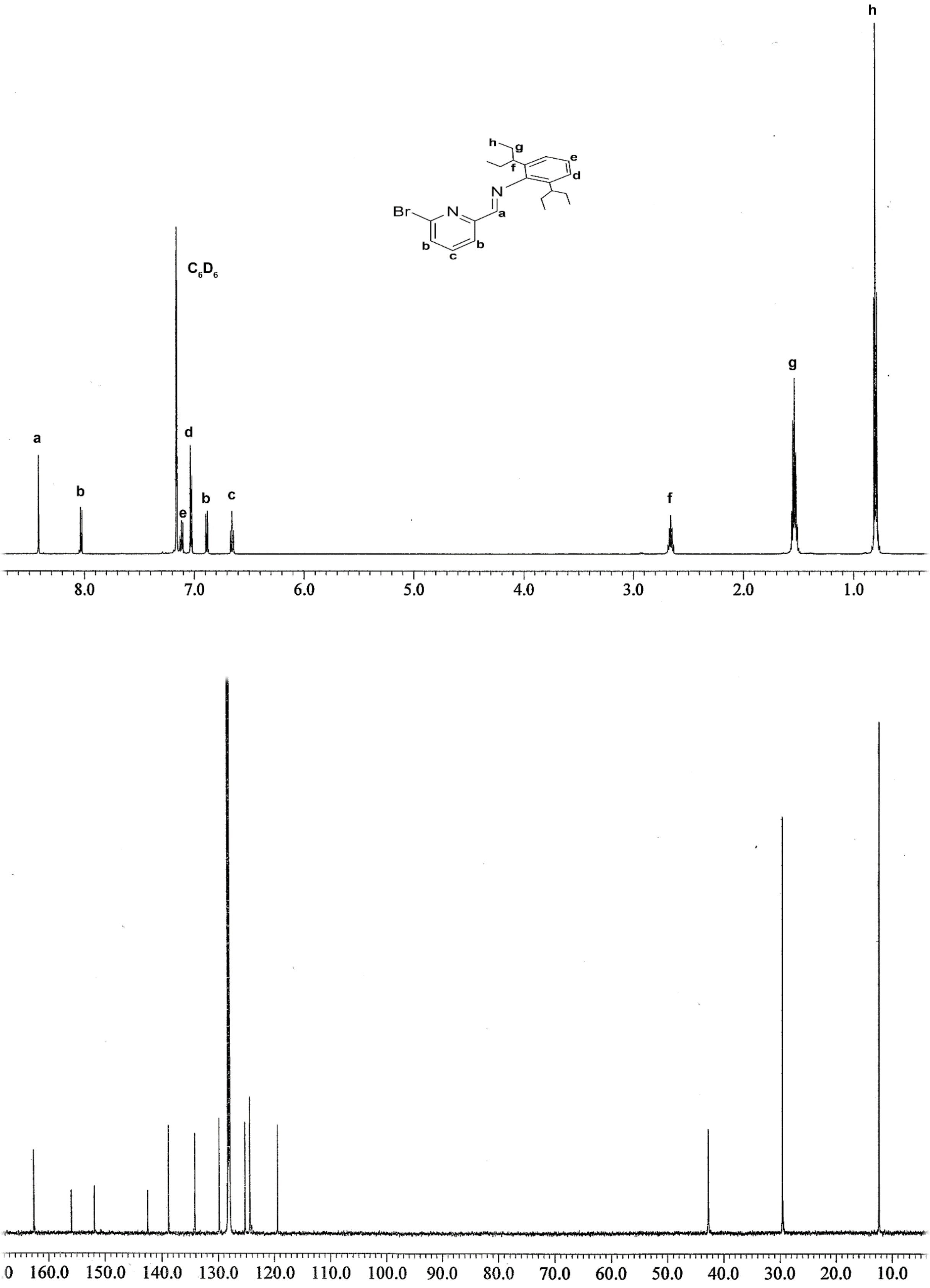

Figure S9. ^{1}H and ^{13}C NMR spectra of **5.**

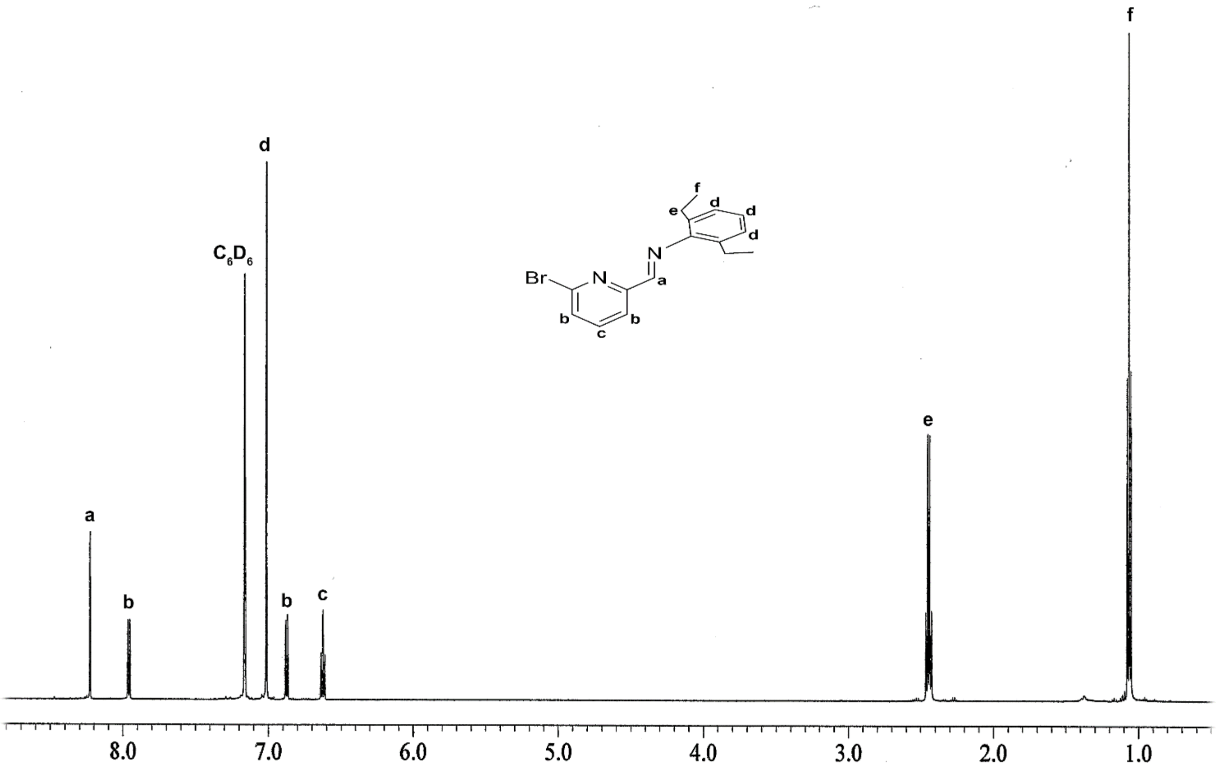

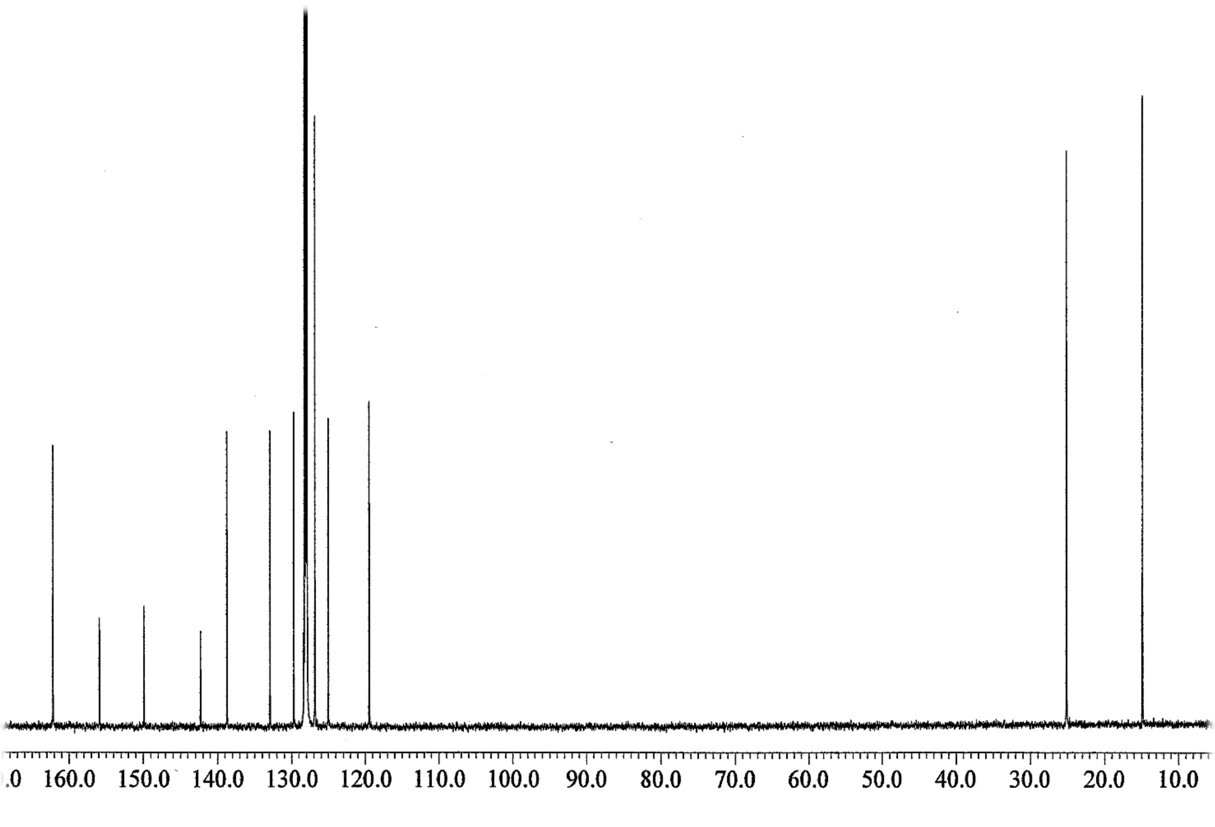

Figure S10. ^{1}H and ^{13}C NMR spectra of **6.**

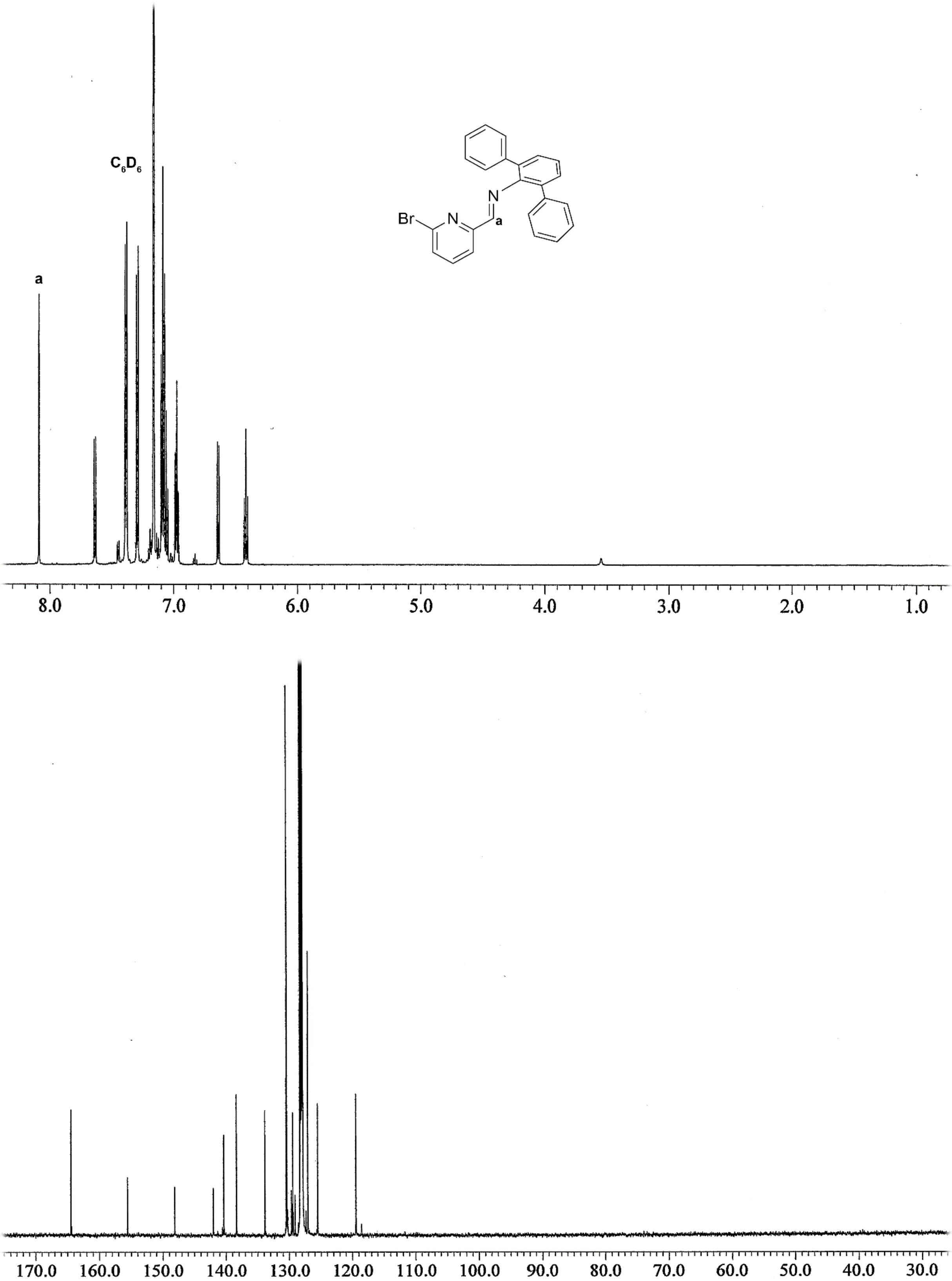

Figure S11. ^{1}H and ^{13}C NMR spectra of **7.**

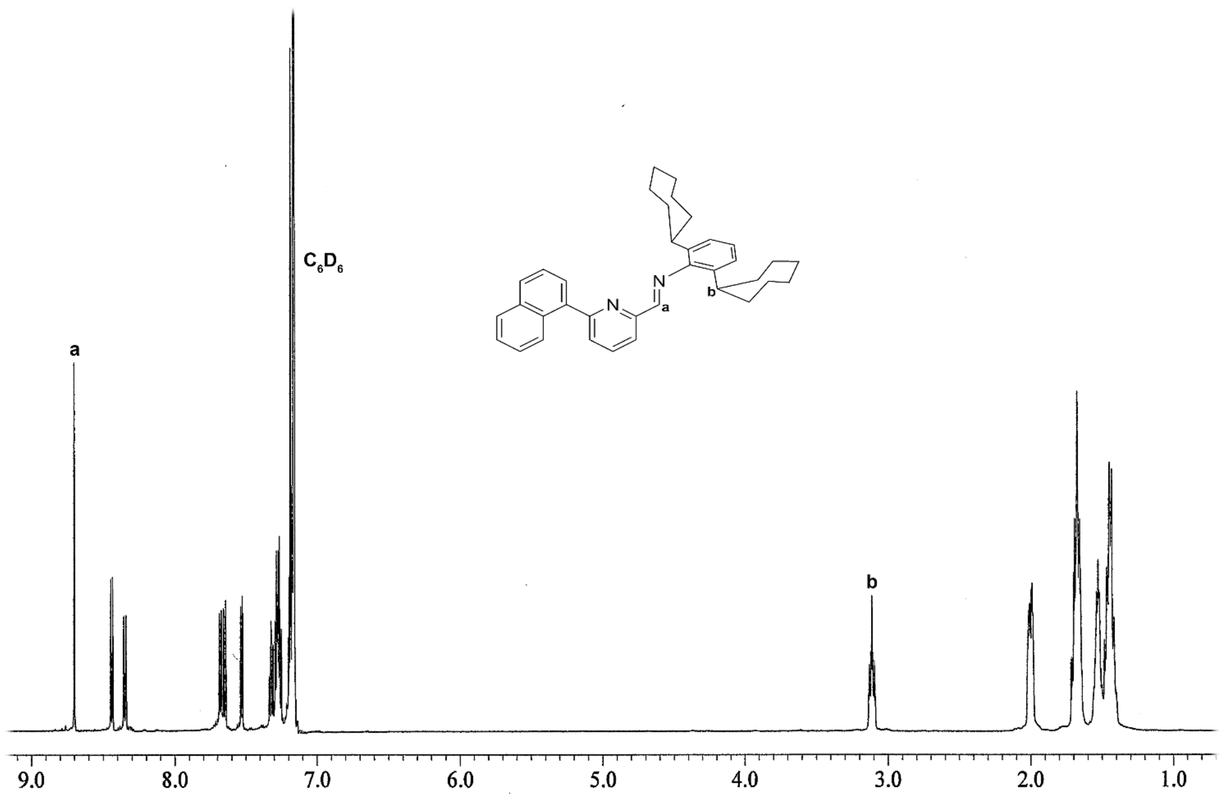

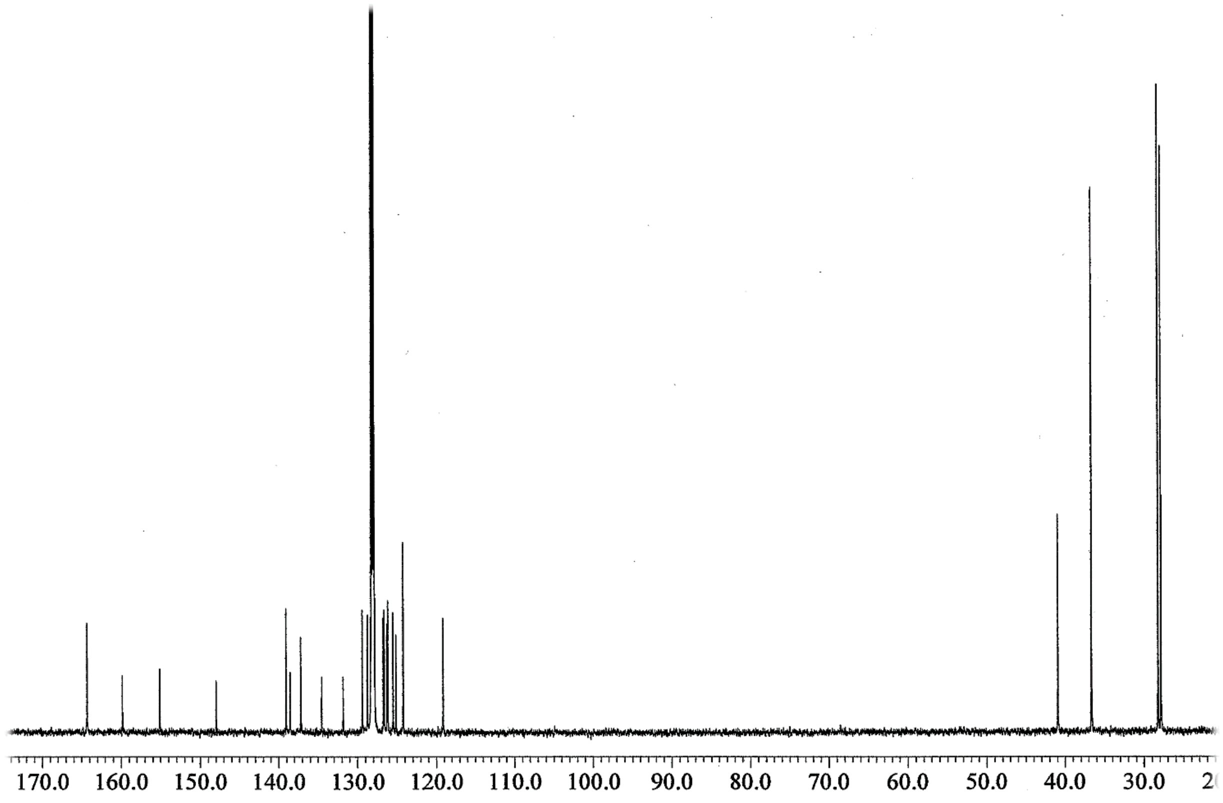

Figure S12. ^{1}H and ^{13}C NMR spectra of **8.**

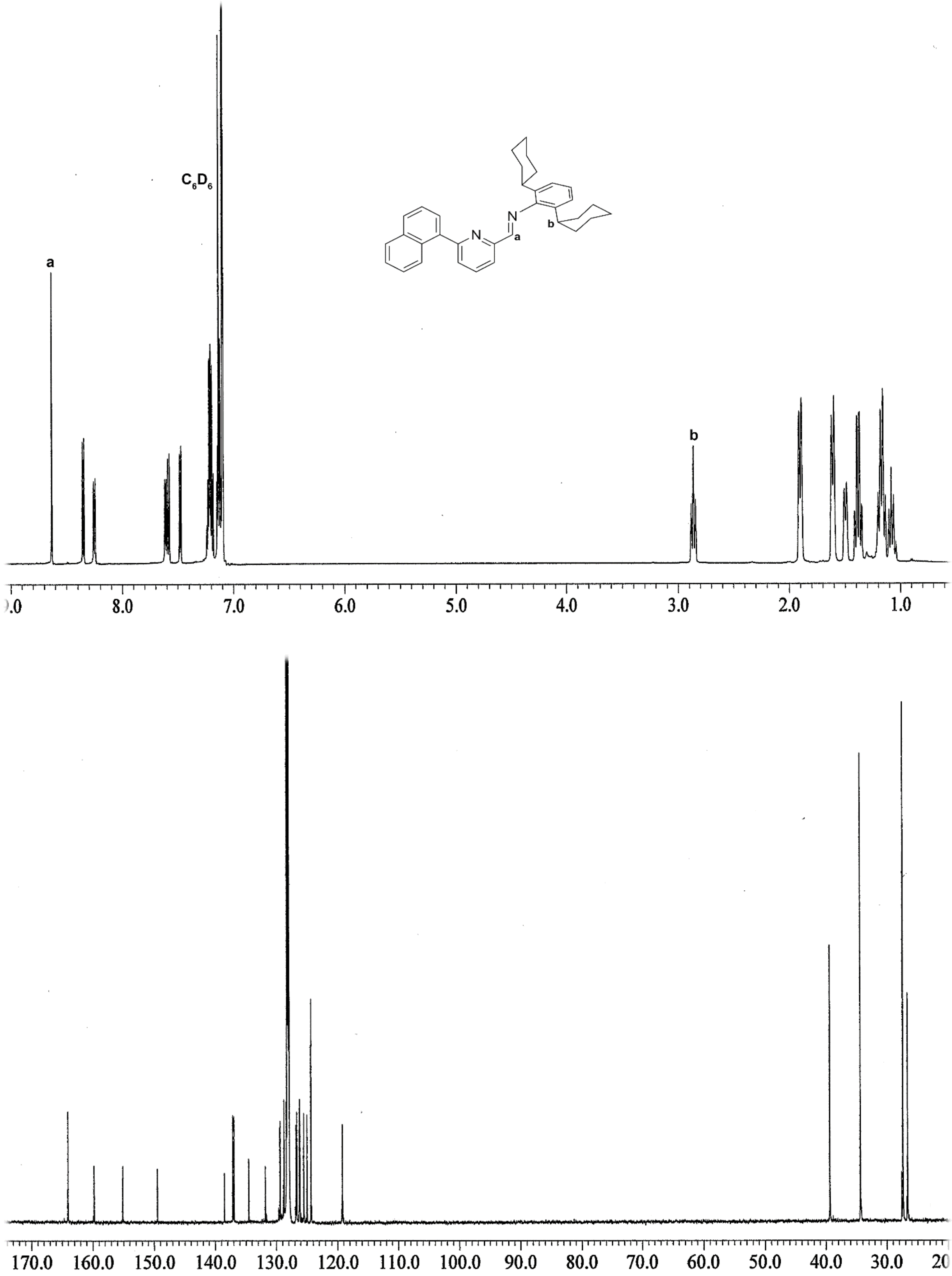

Figure S13. ^{1}H and ^{13}C NMR spectra of **9.**

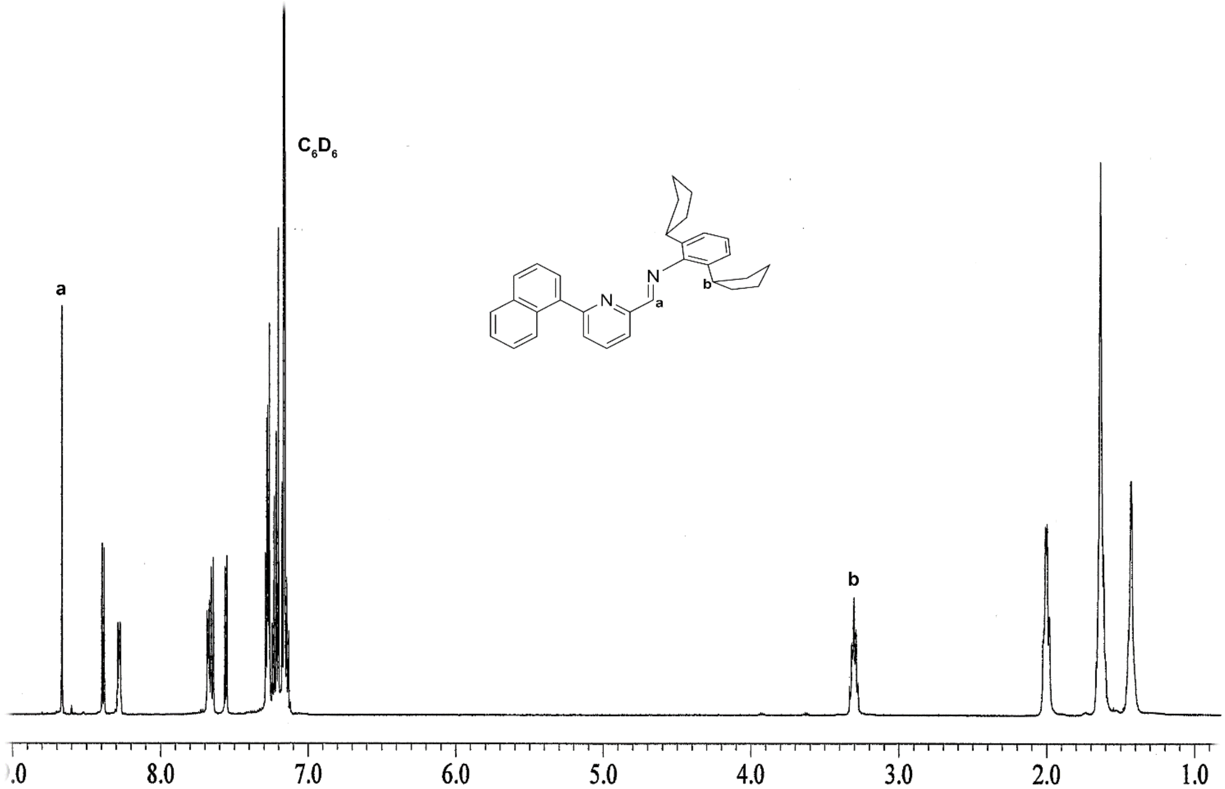

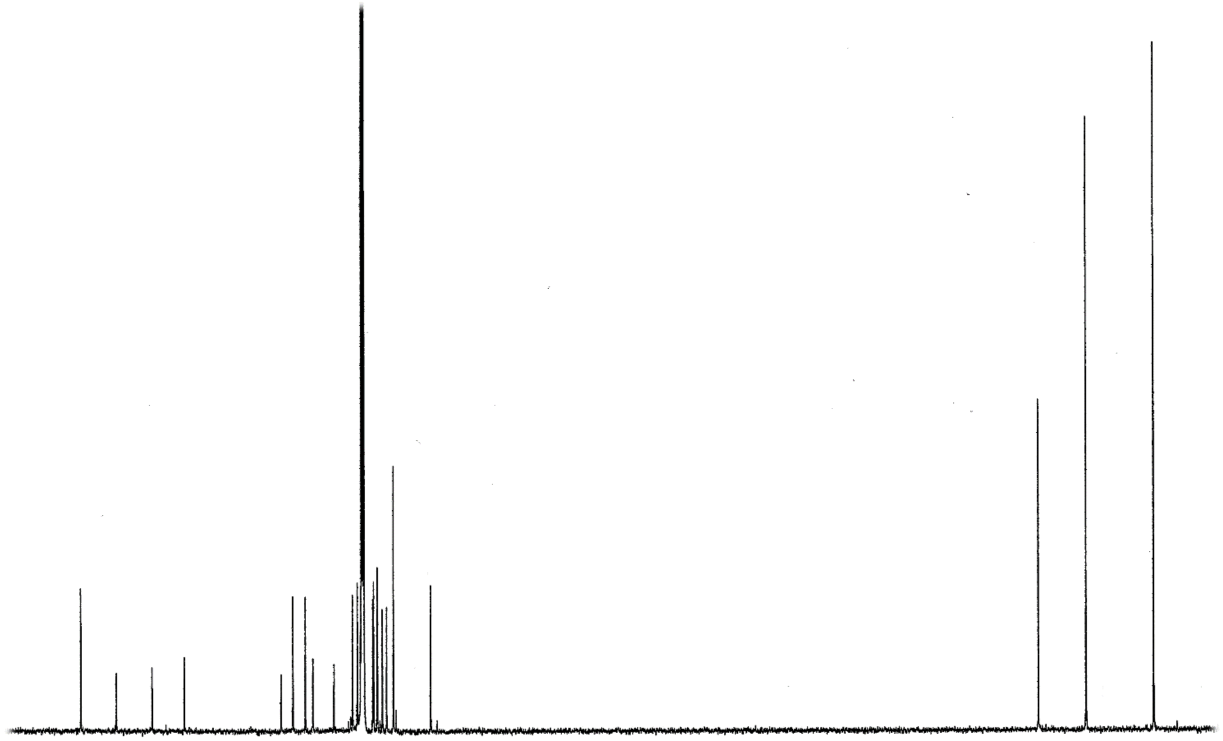

Figure S14. ^{1}H and ^{13}C NMR spectra of **10.**

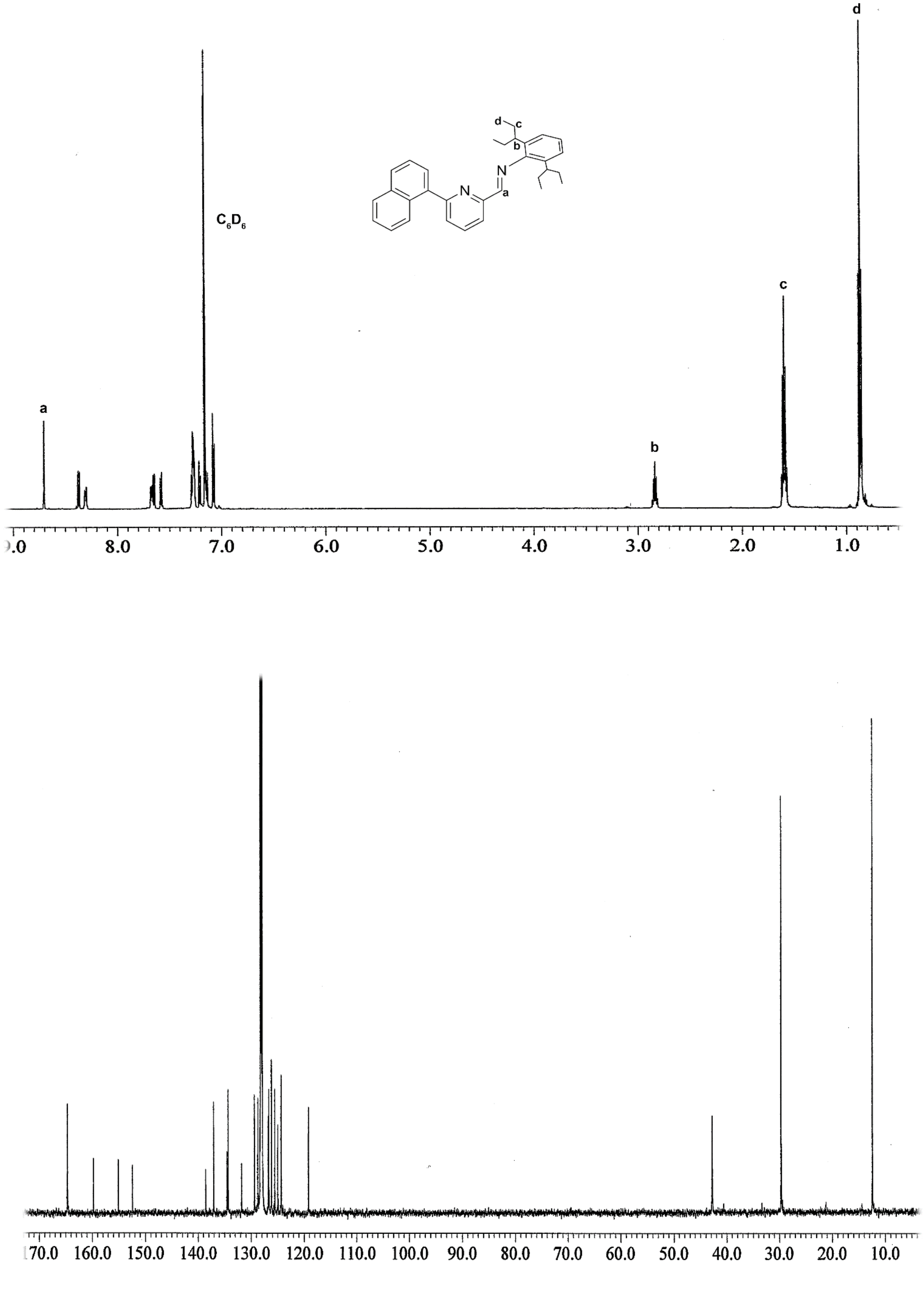

Figure S15. ^{1}H and ^{13}C NMR spectra of **11.**

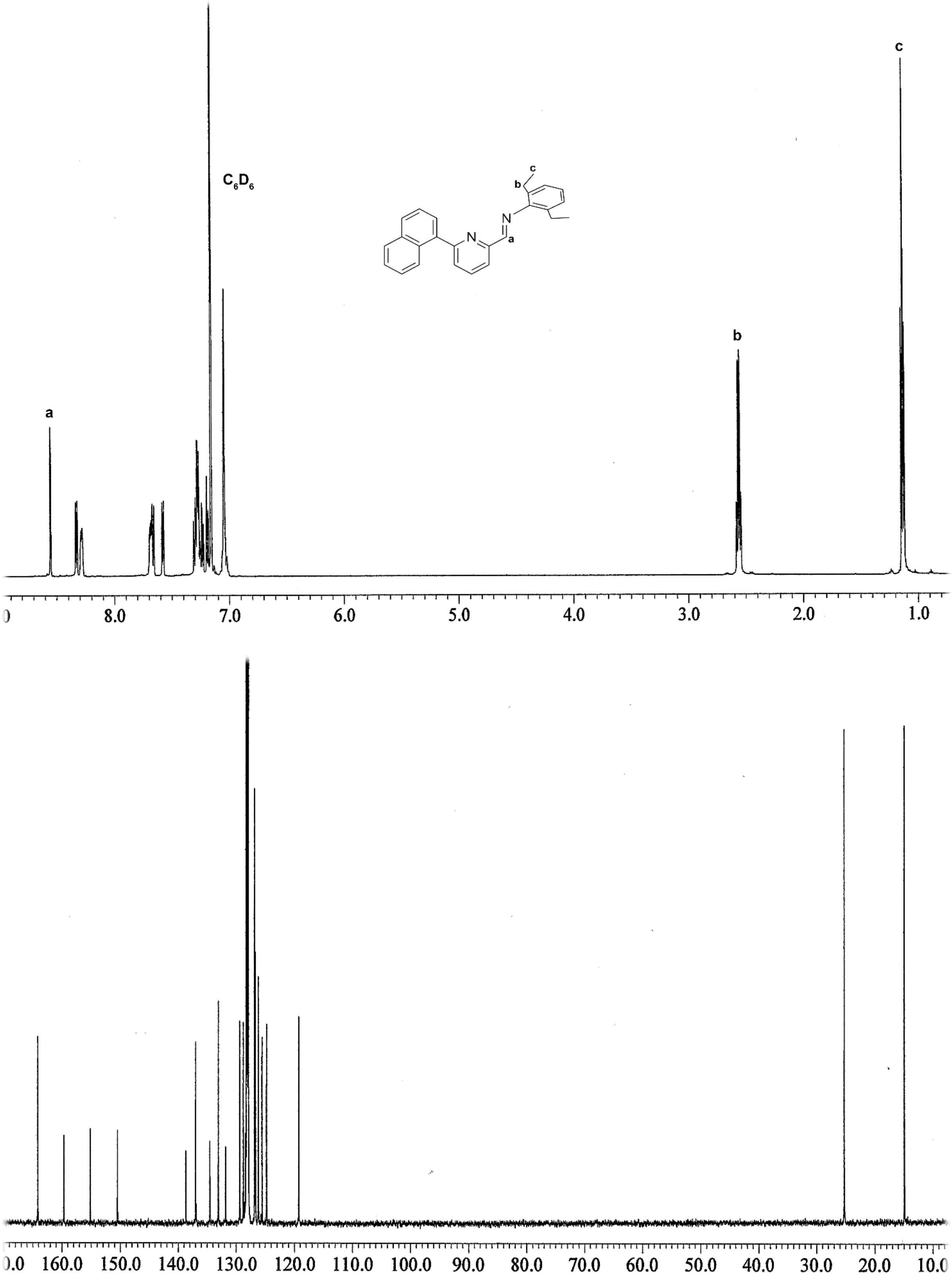

Figure S16. ^{1}H and ^{13}C NMR spectra of **12.**

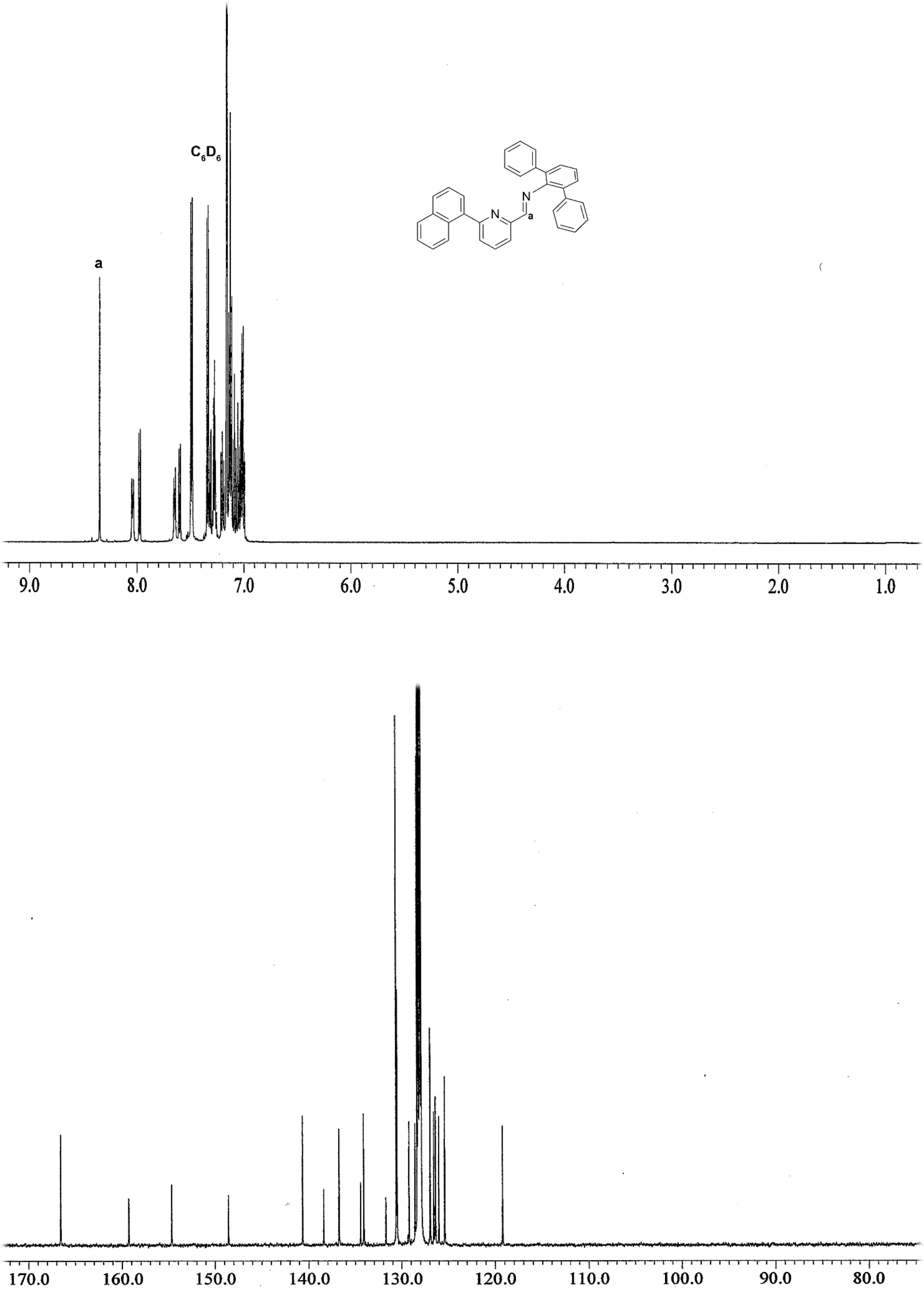

Figure S17. ^{1}H and ^{13}C NMR spectra of **13.**

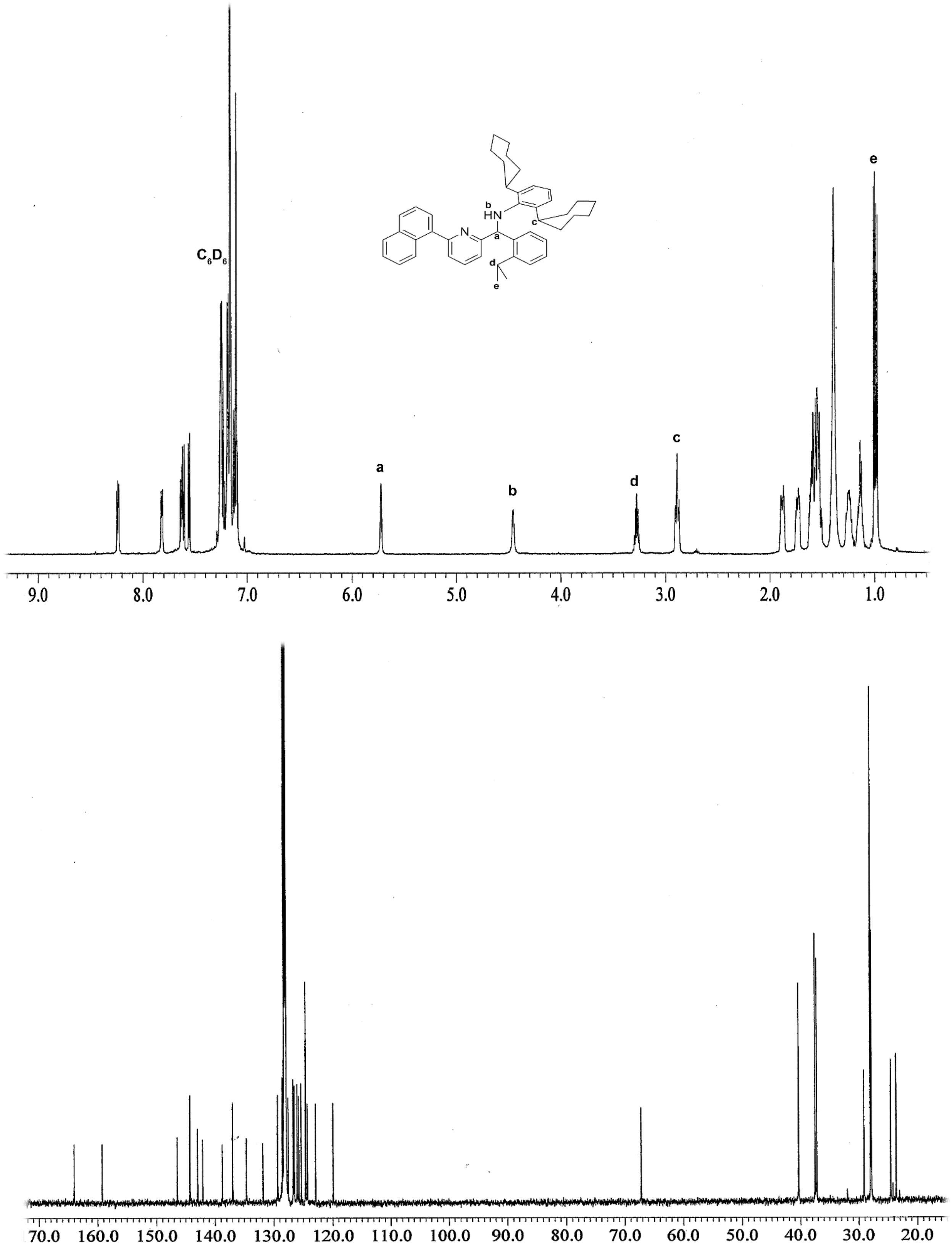

Figure S18. ^{1}H and ^{13}C NMR spectra of **14.**

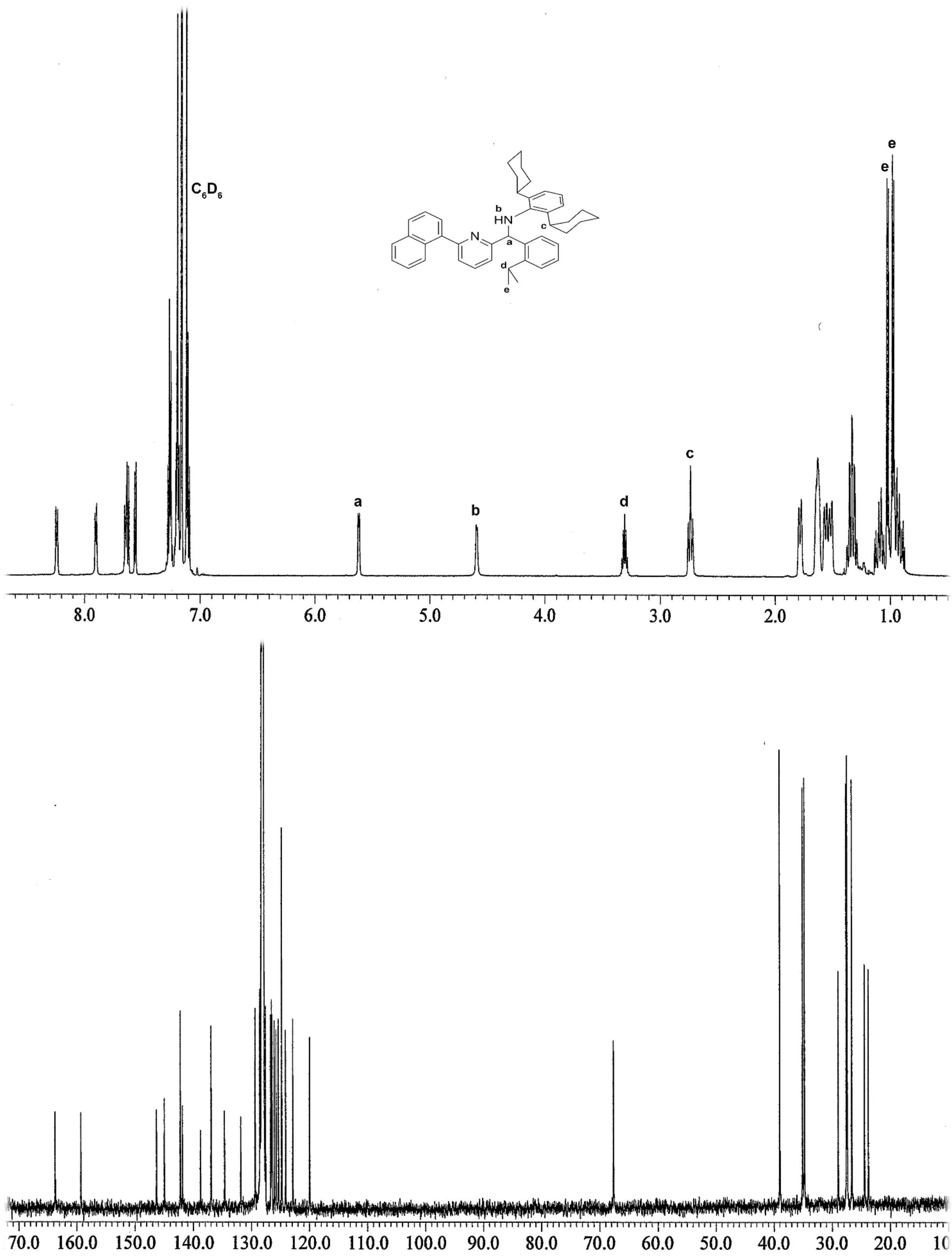

Figure S19. ^{1}H and ^{13}C NMR spectra of **15.**

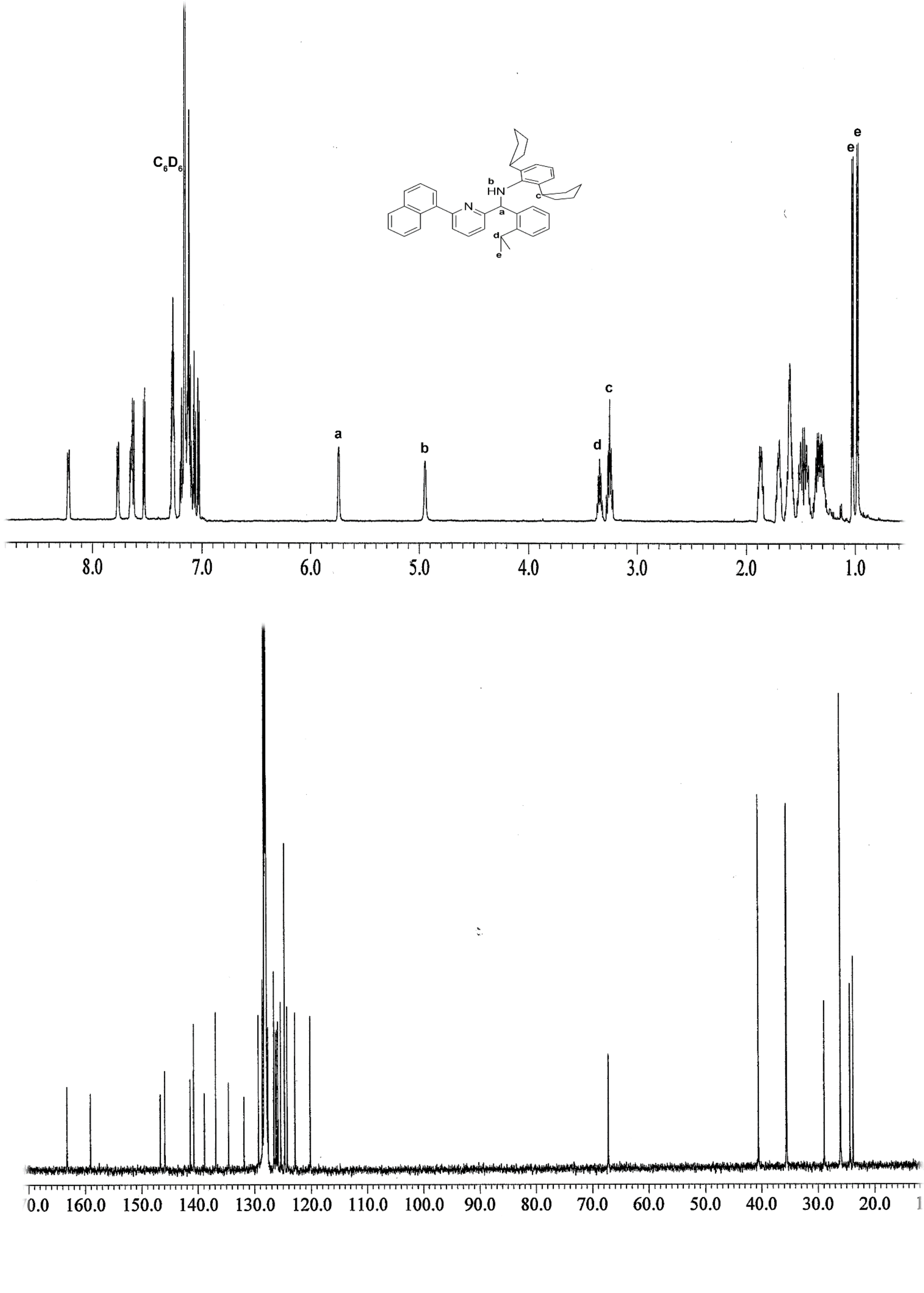

Figure S20. ^{1}H and ^{13}C NMR spectra of **16.**

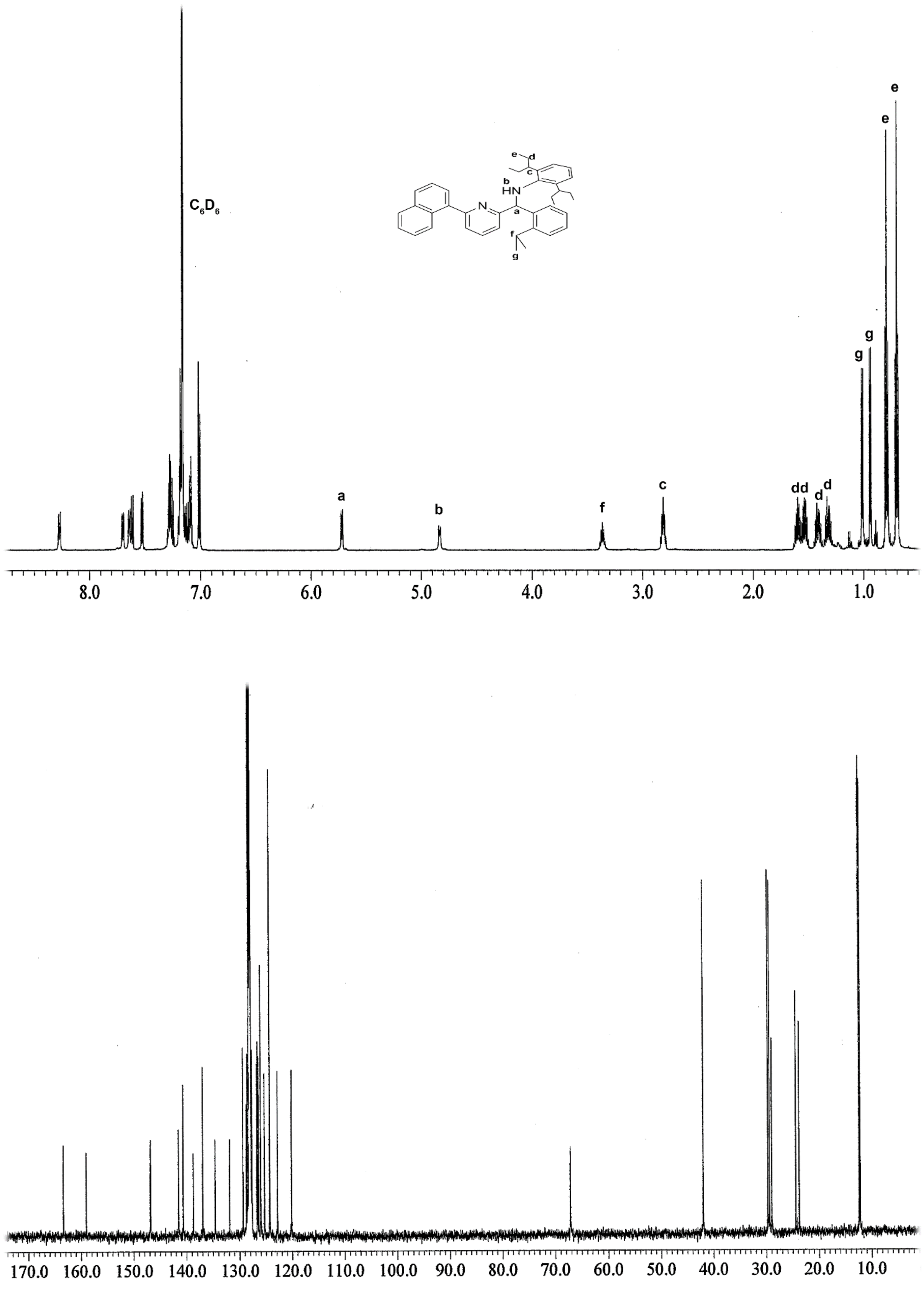

Figure S21. ^{1}H and ^{13}C NMR spectra of **17.**

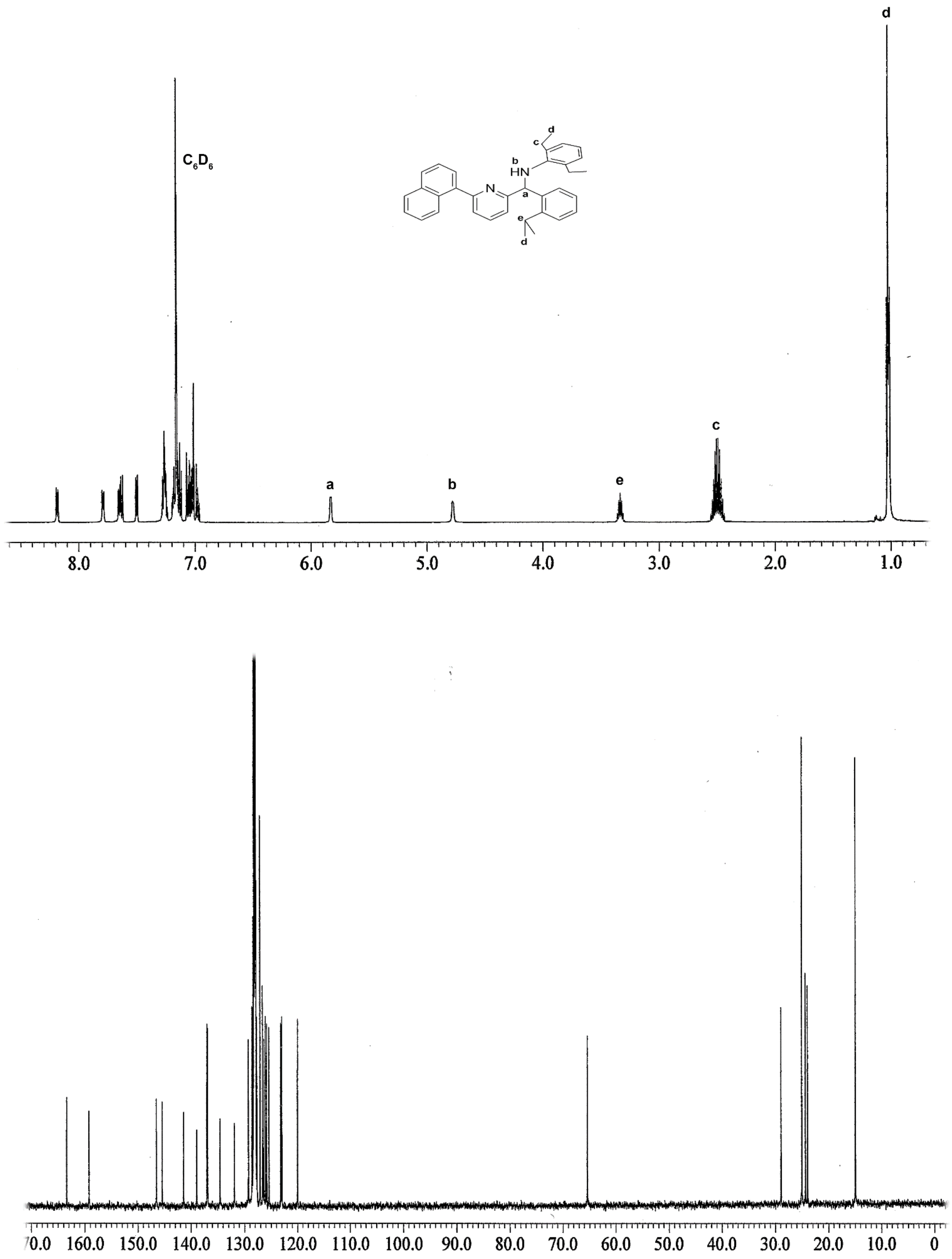

Figure S22. ^{1}H and ^{13}C NMR spectra of **18.**

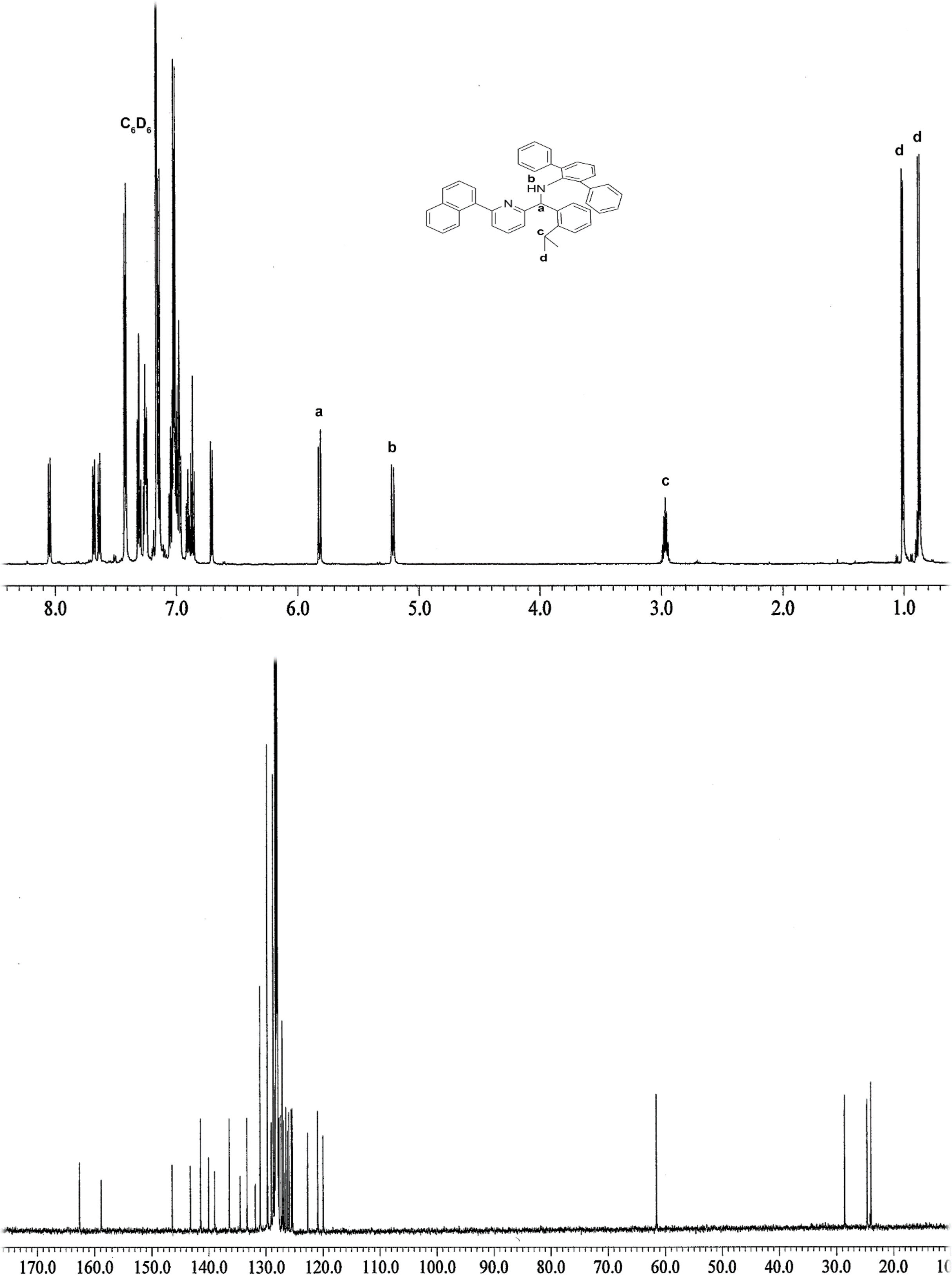

Figure S23. ^{1}H and ^{13}C NMR spectra of **19.**

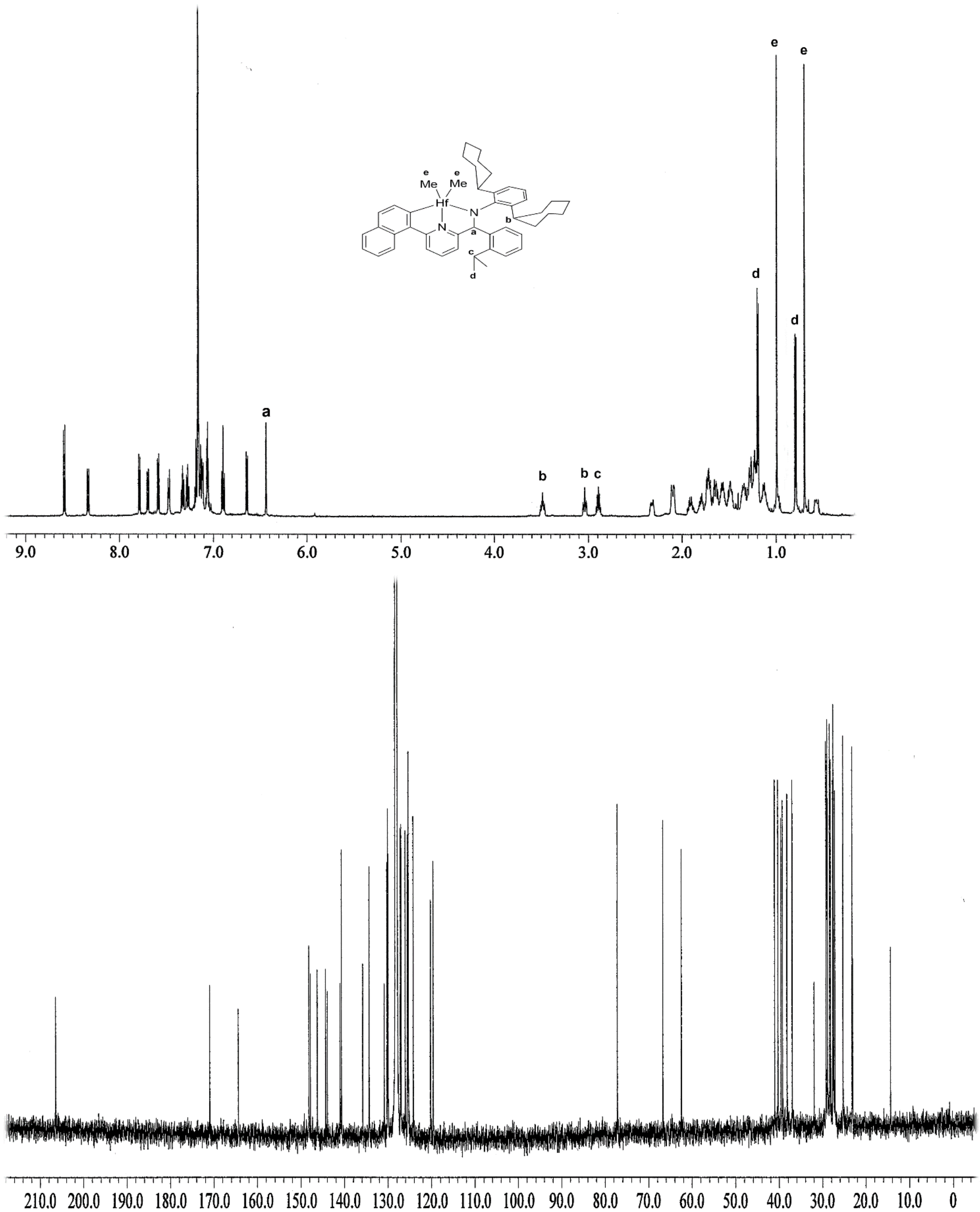

Figure S24. ^{1}H and ^{13}C NMR spectra of **20.**

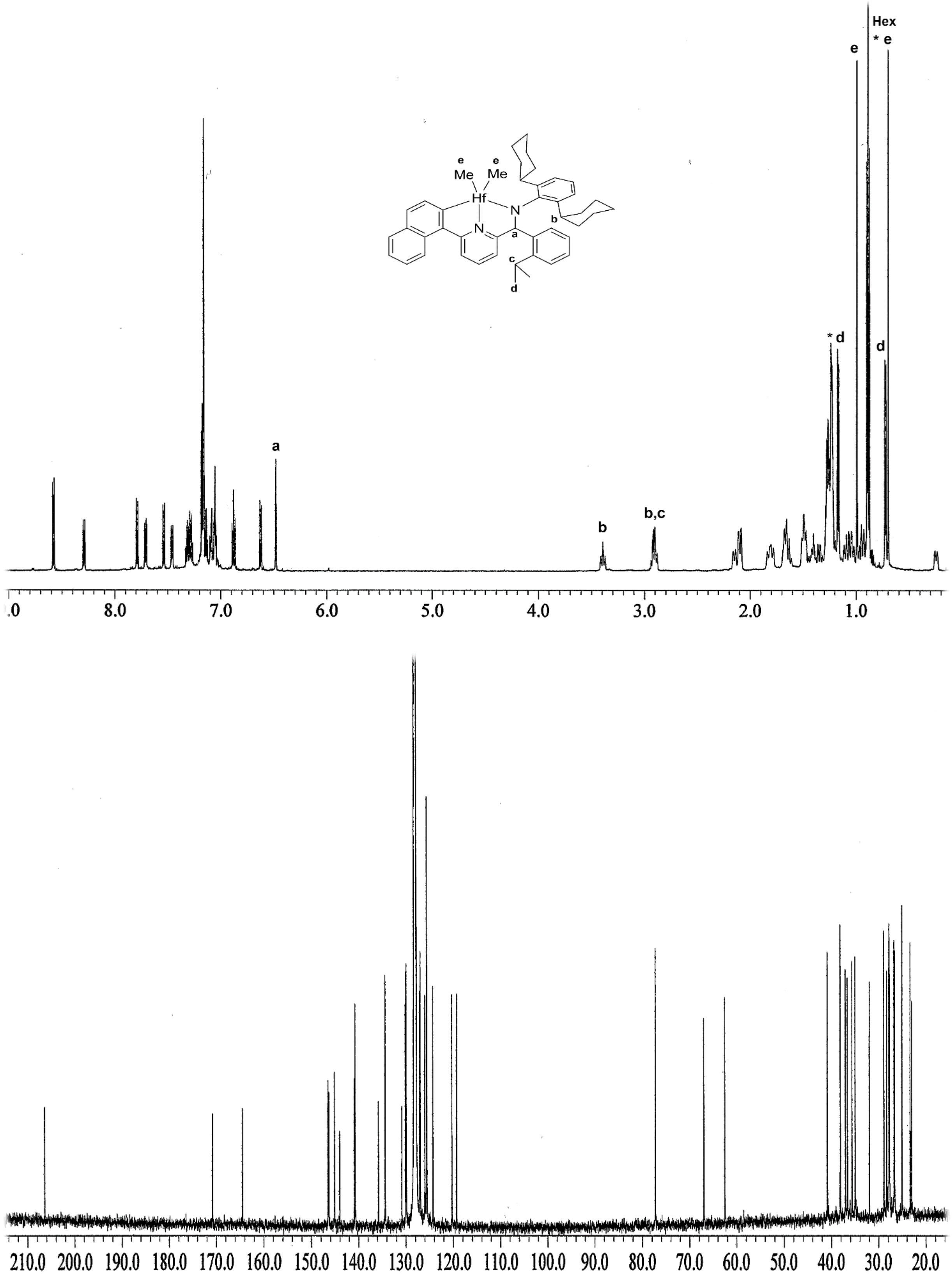

Figure S25. ^{1}H and ^{13}C NMR spectra of **21.**

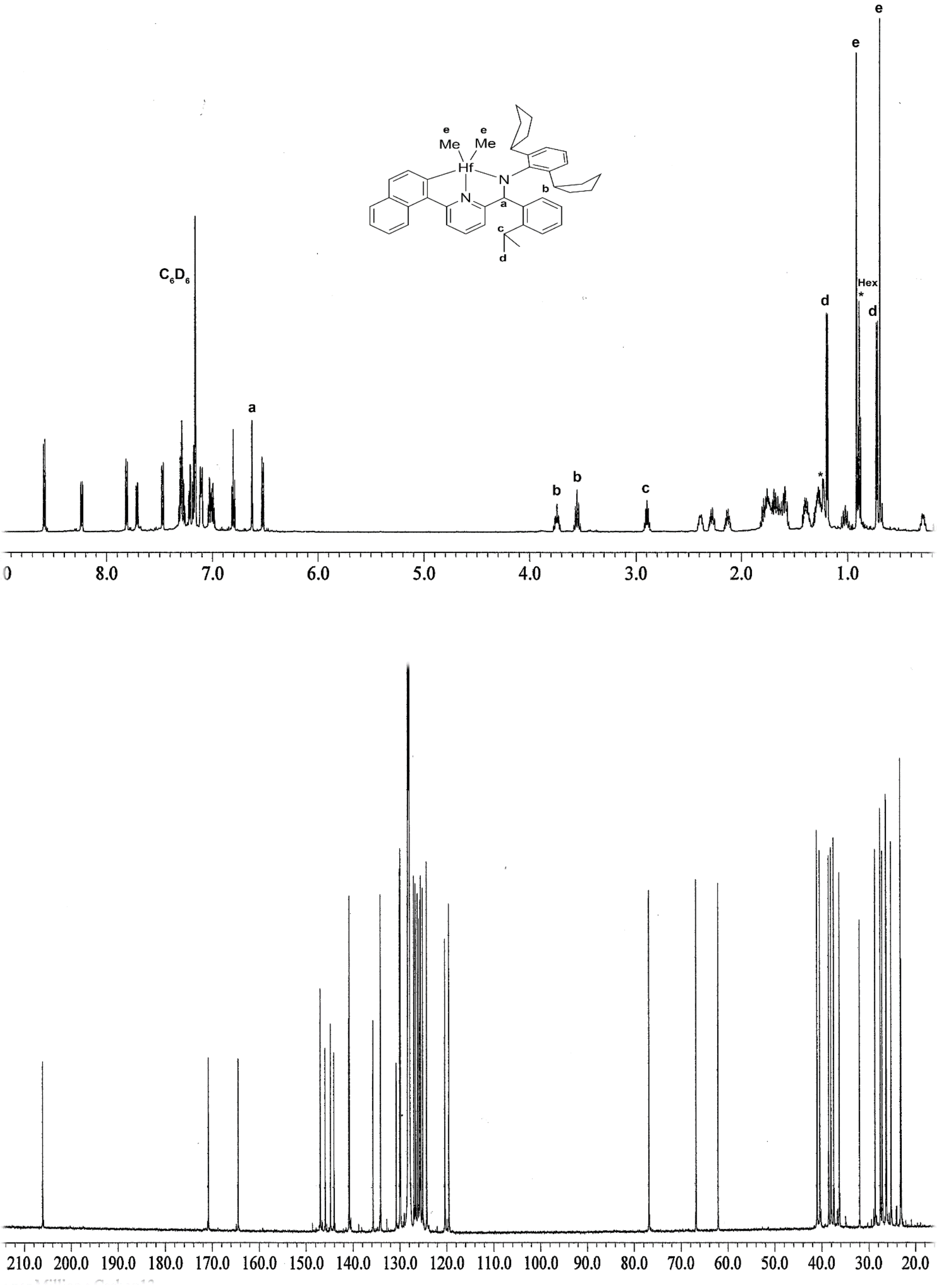

Figure S26. ^{1}H and ^{13}C NMR spectra of **22.**

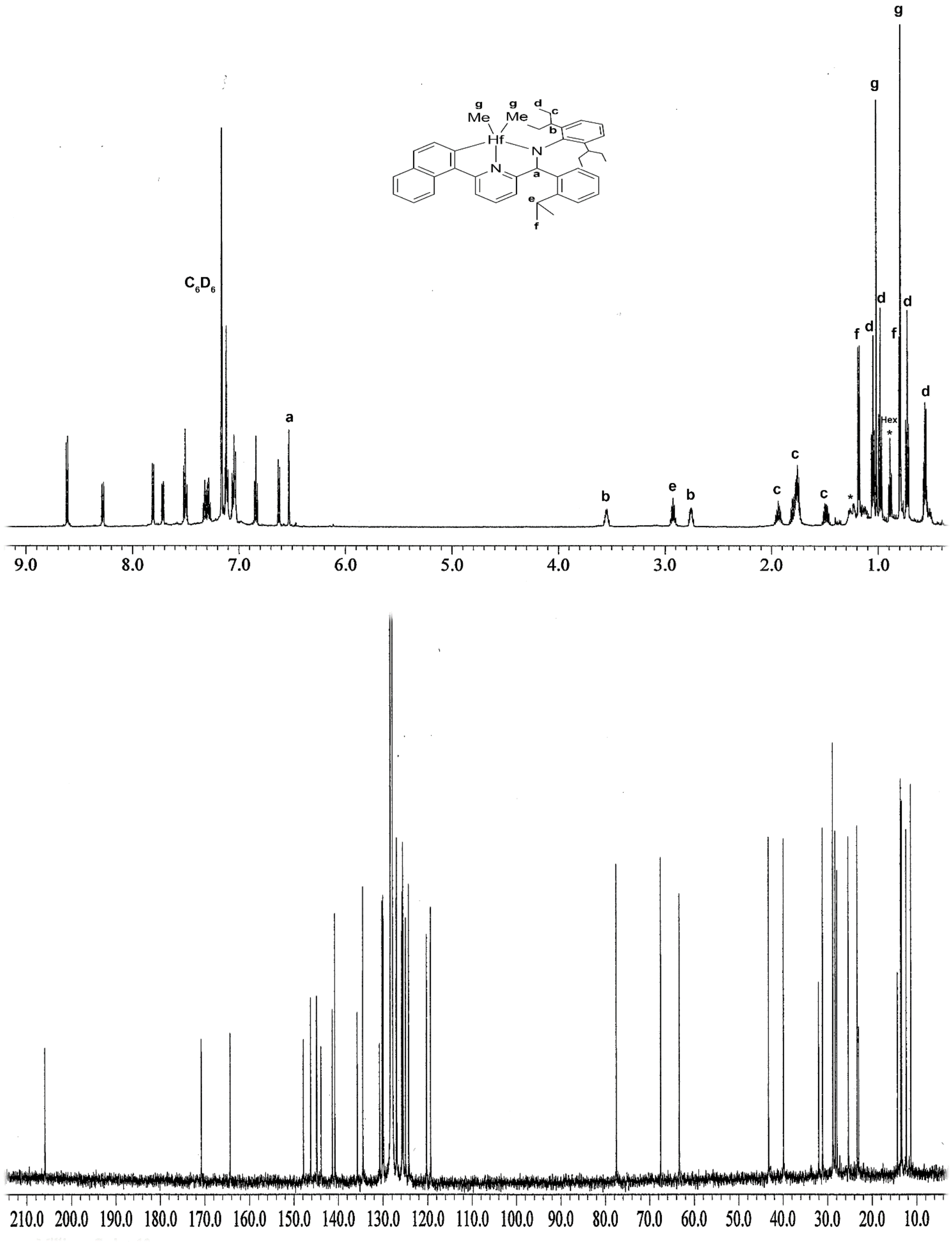

Figure S27. ^{1}H and ^{13}C NMR spectra of **23.**

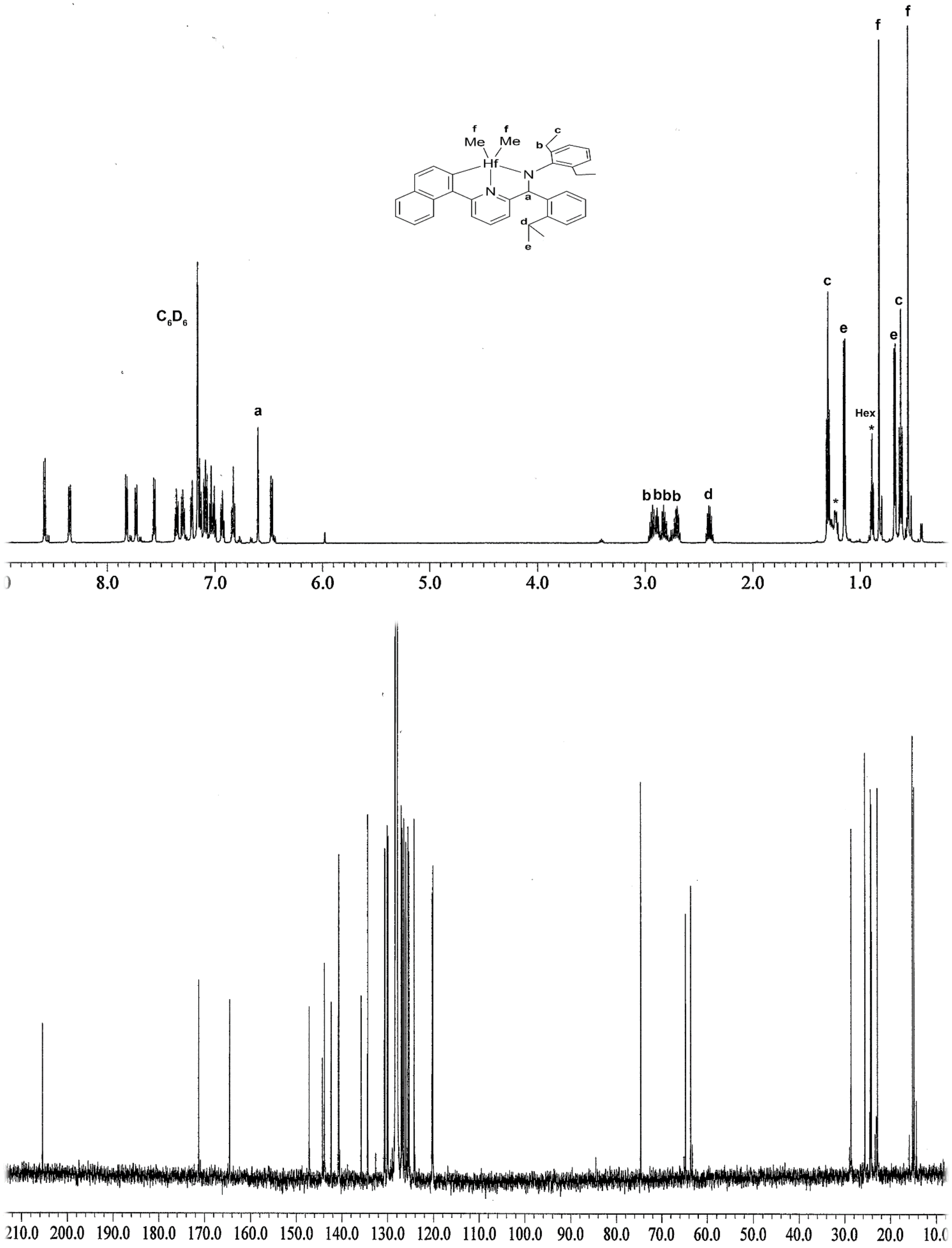

Figure S28. ^{1}H and ^{13}C NMR spectra of **24.**

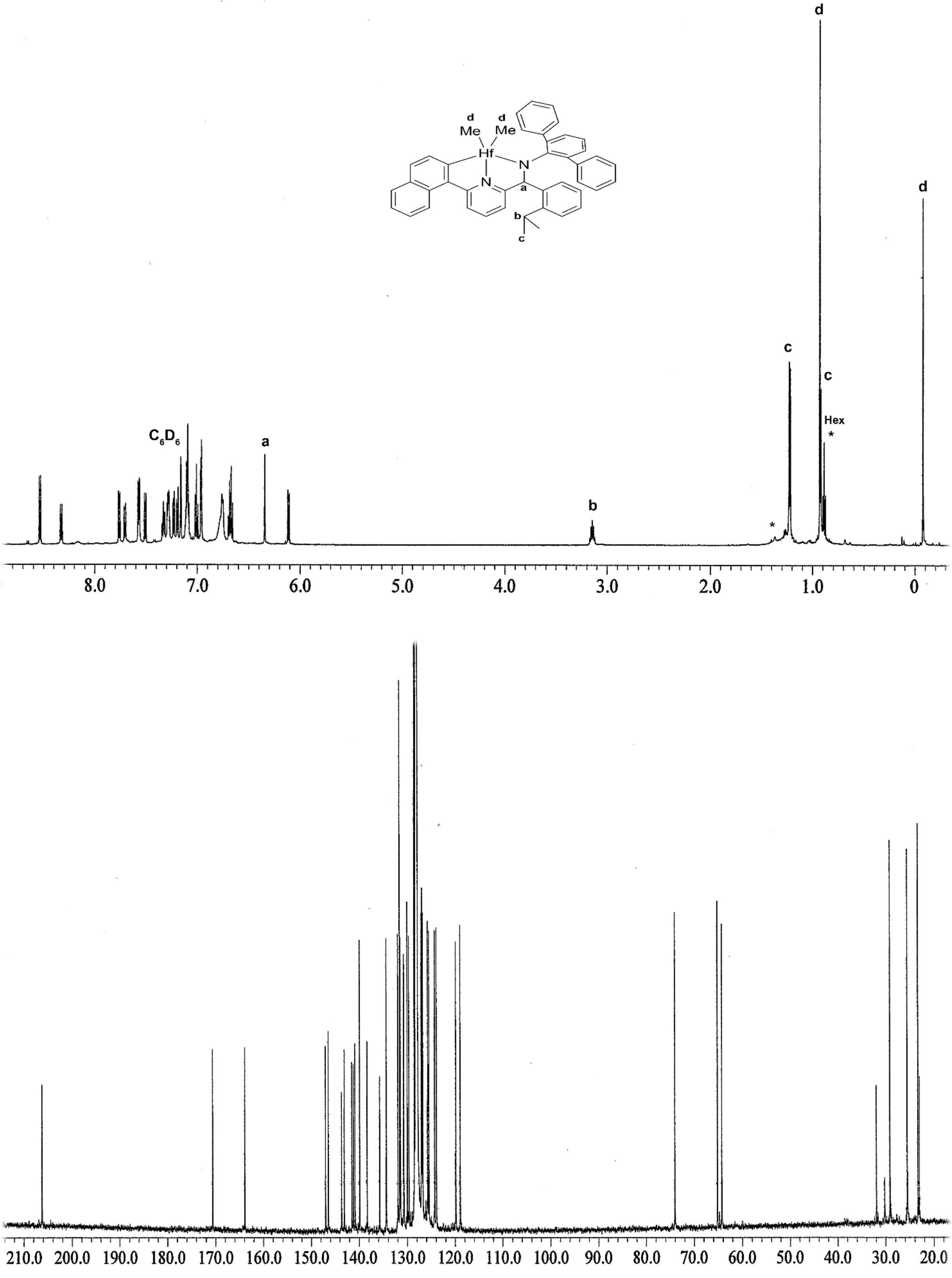

Figure S29. ^{1}H and ^{13}C NMR spectra of **25.**

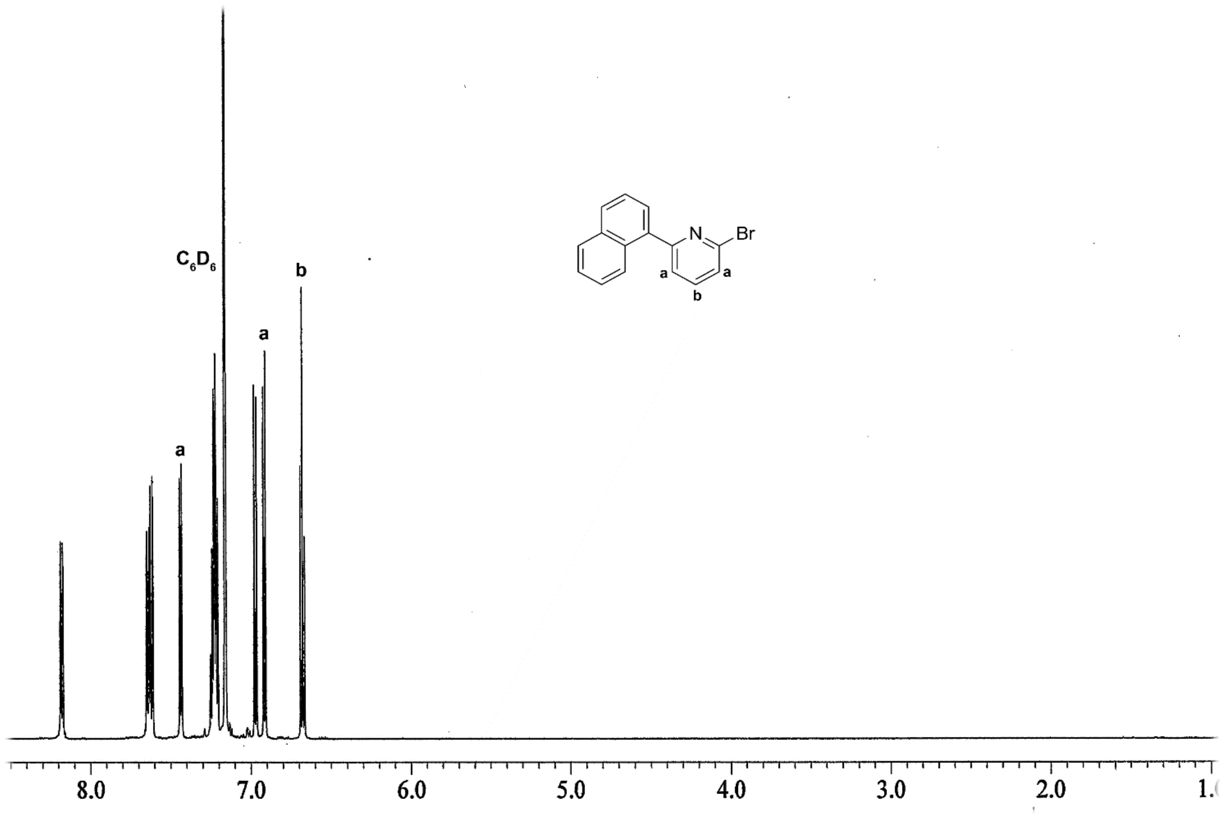

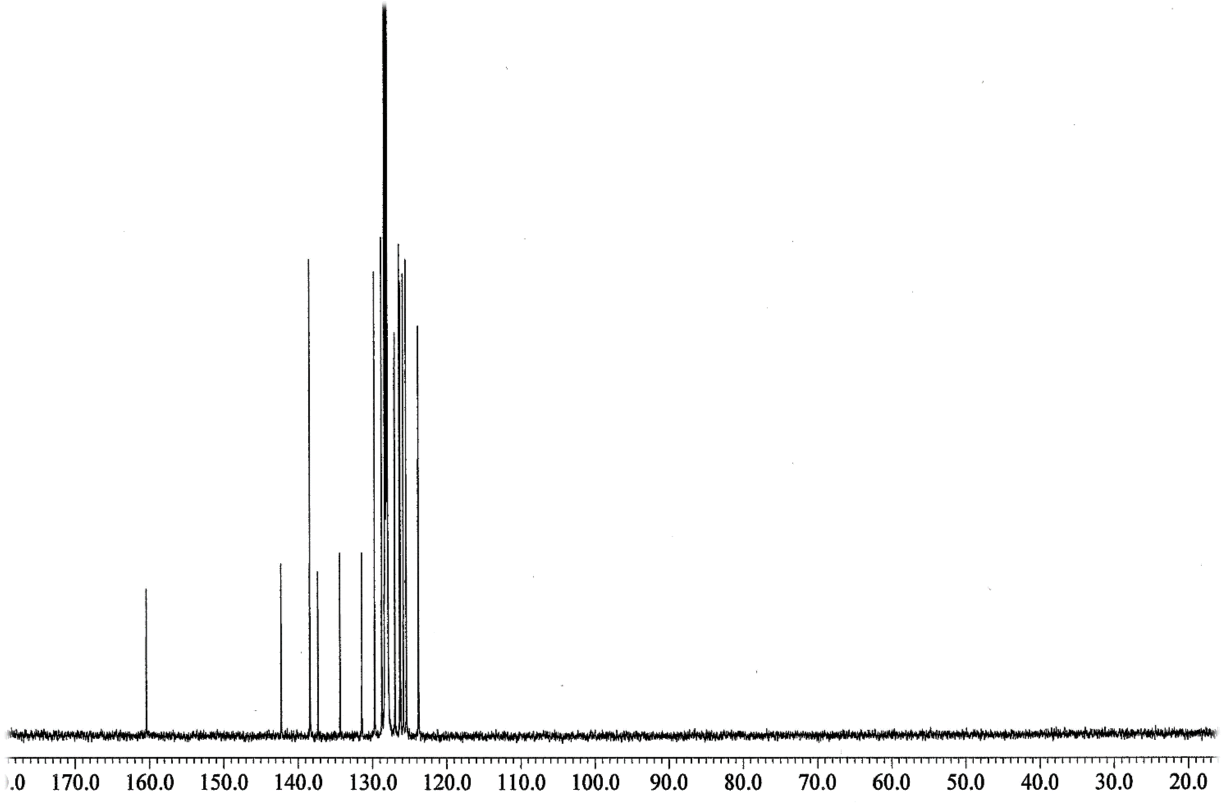

Figure S30. ^{1}H and ^{13}C NMR spectra of **26.**

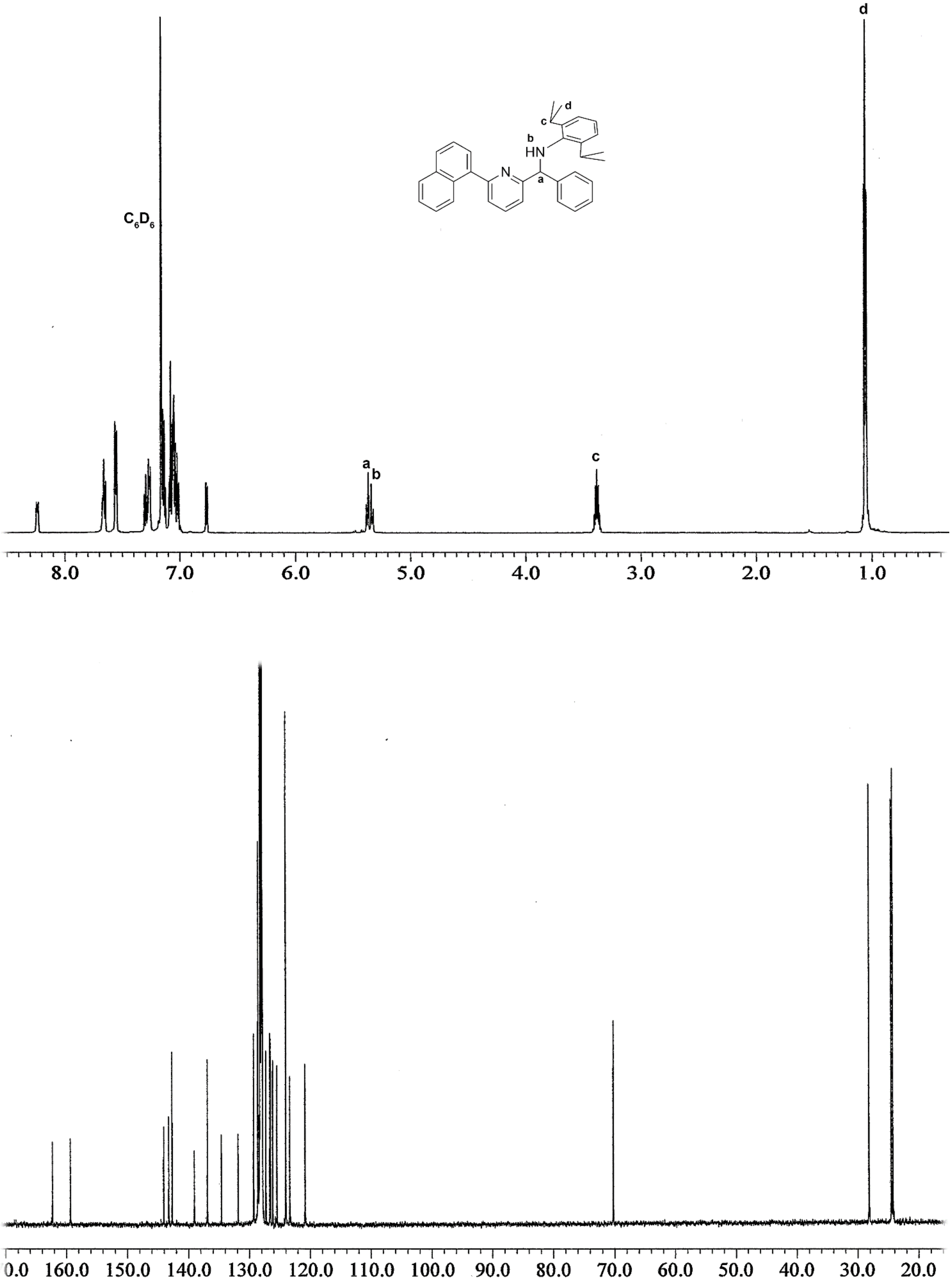

Figure S31. ^{1}H and ^{13}C NMR spectra of **27.**

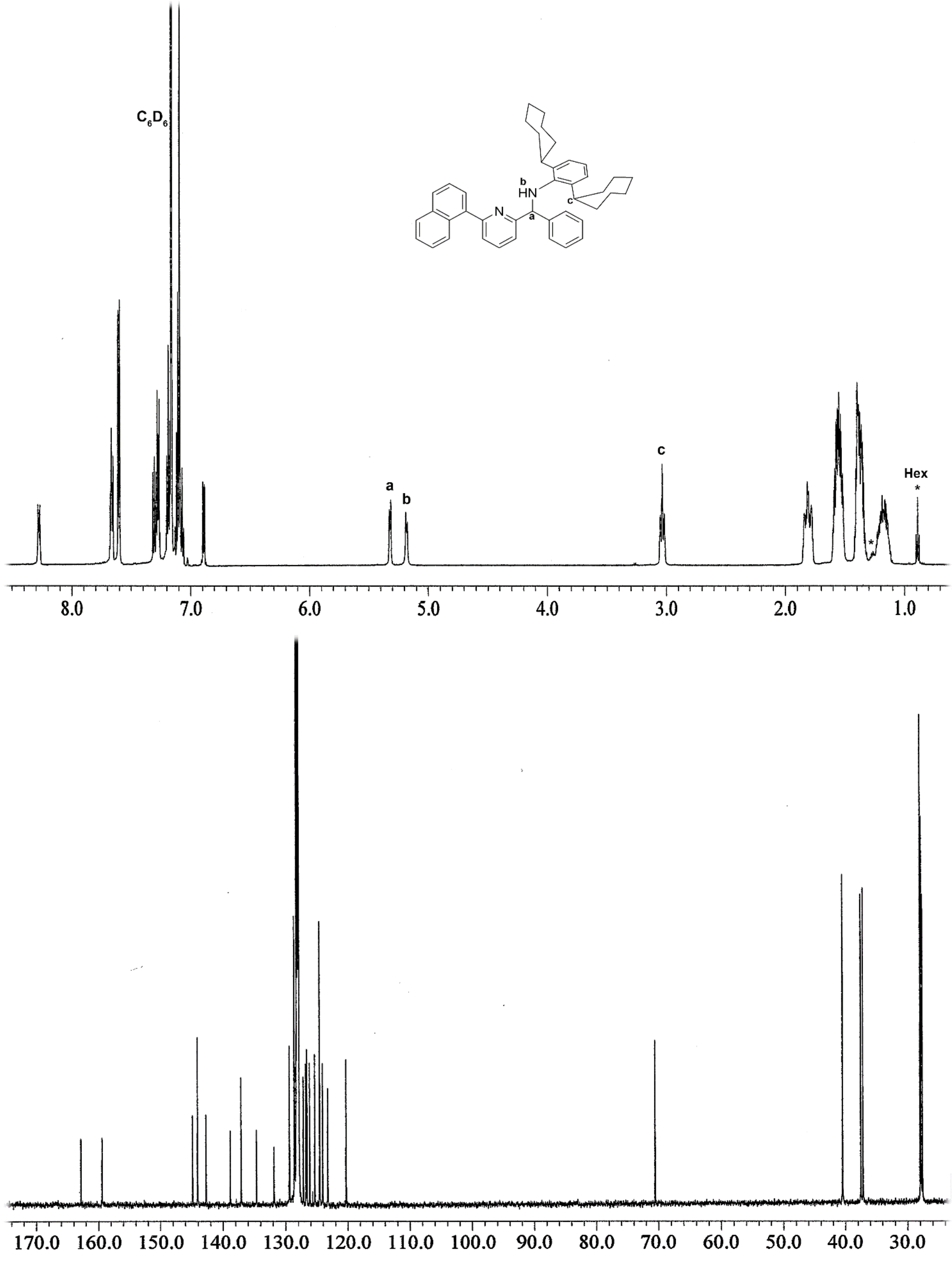

Figure S32. ^{1}H and ^{13}C NMR spectra of **28.**

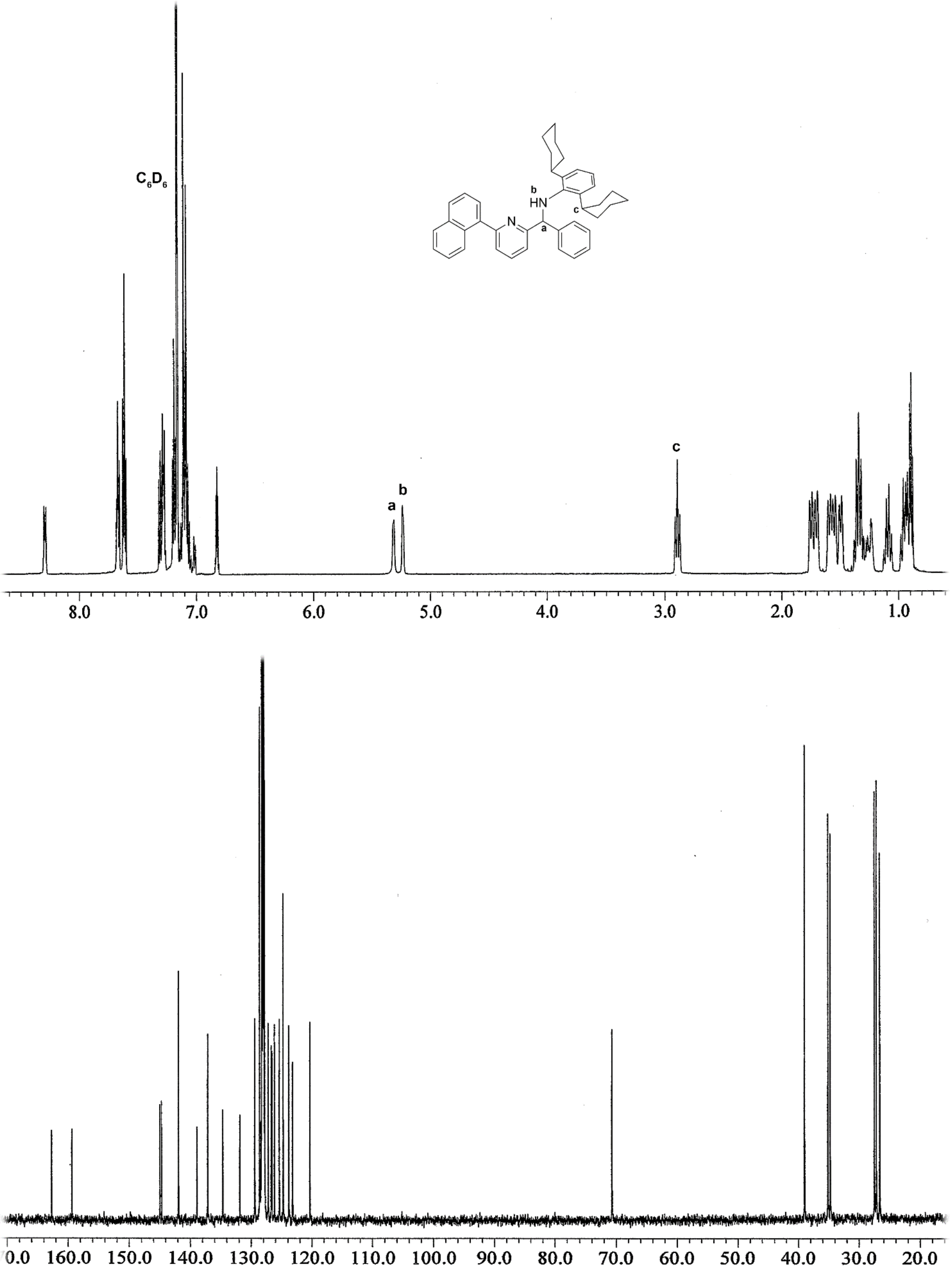

Figure S33. ^{1}H and ^{13}C NMR spectra of **29.**

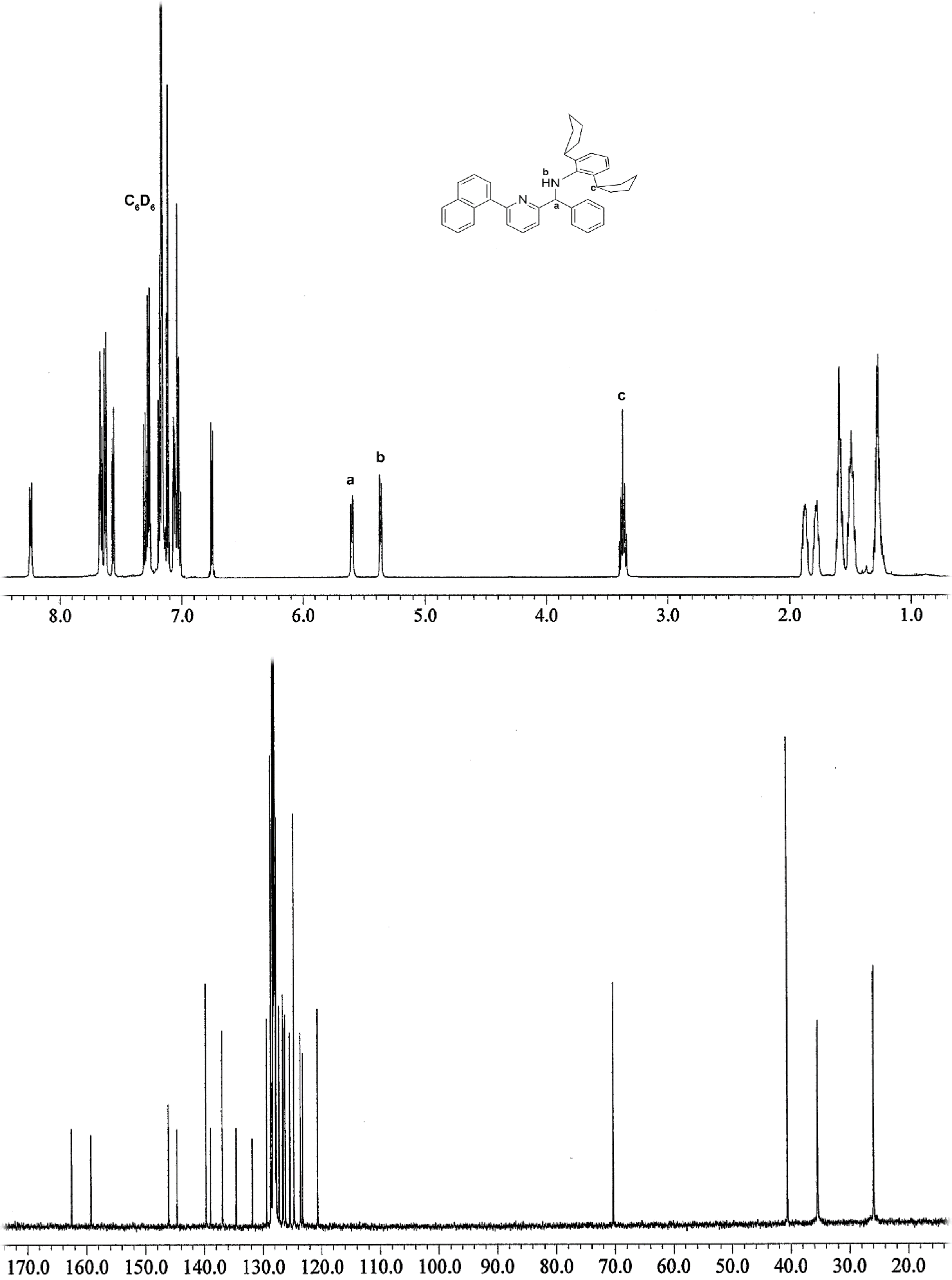

Figure S34. ^{1}H and ^{13}C NMR spectra of **30.**

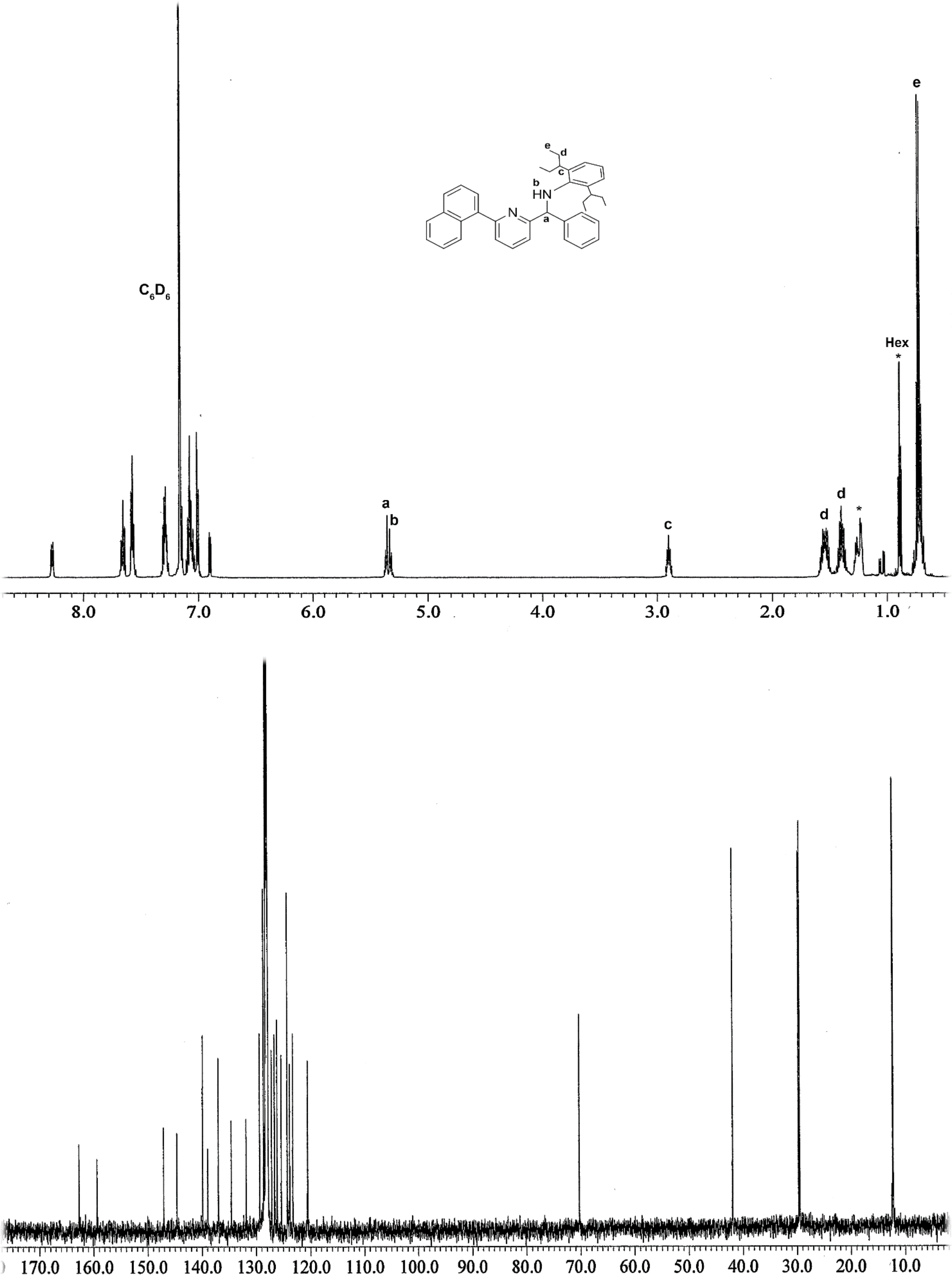

Figure S35. ^{1}H and ^{13}C NMR spectra of **31.**

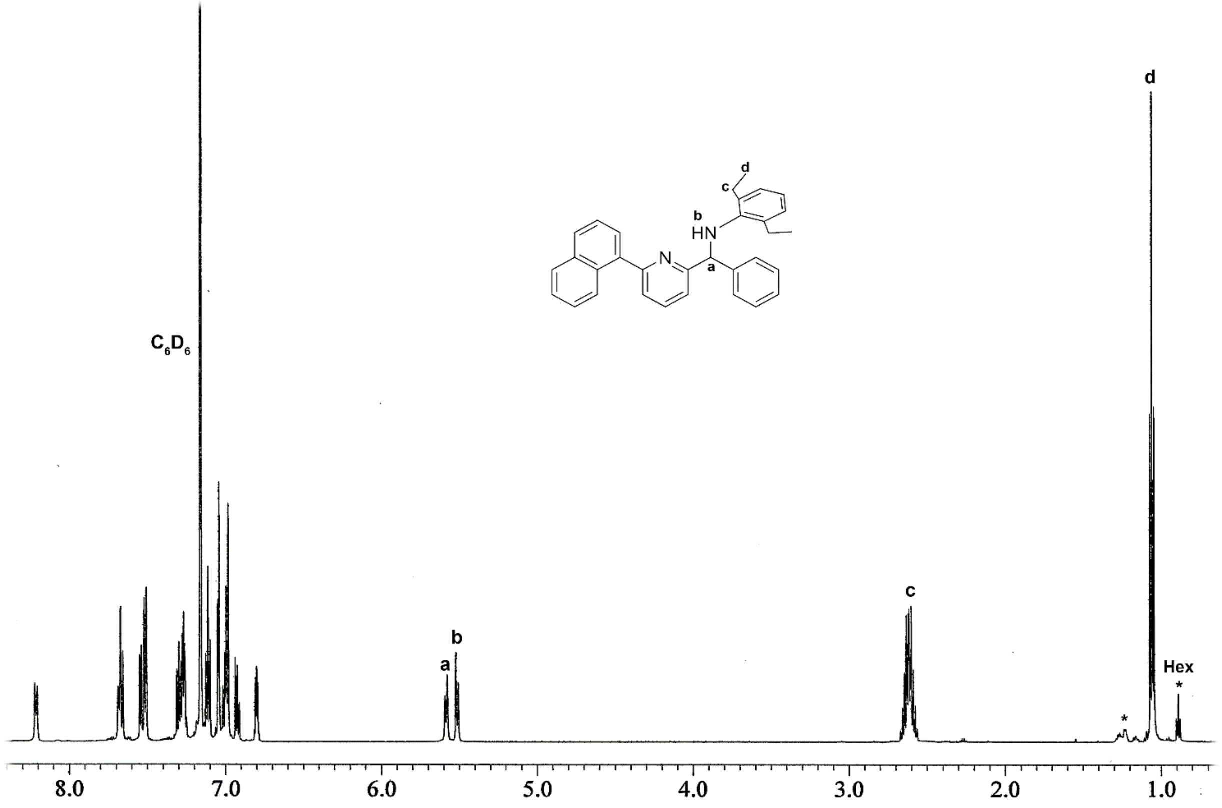

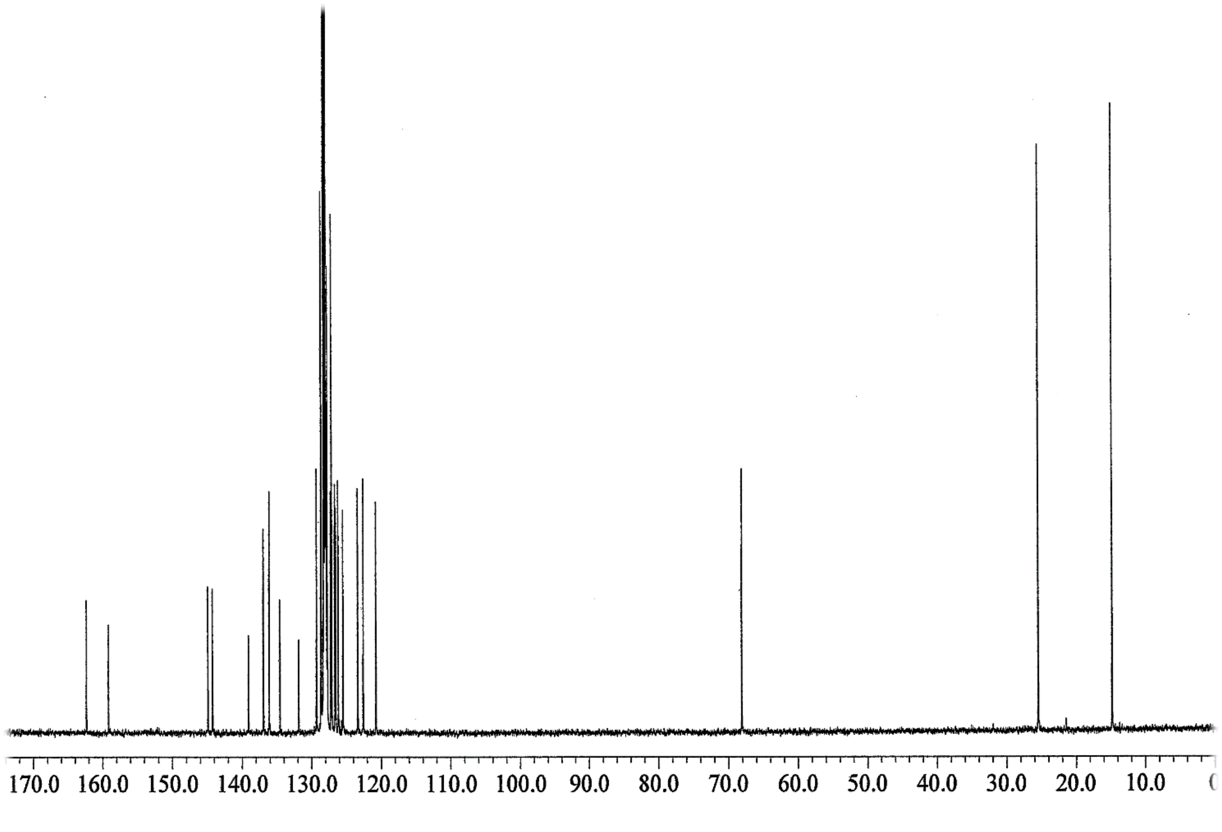

Figure S36. ^{1}H and ^{13}C NMR spectra of **32.**

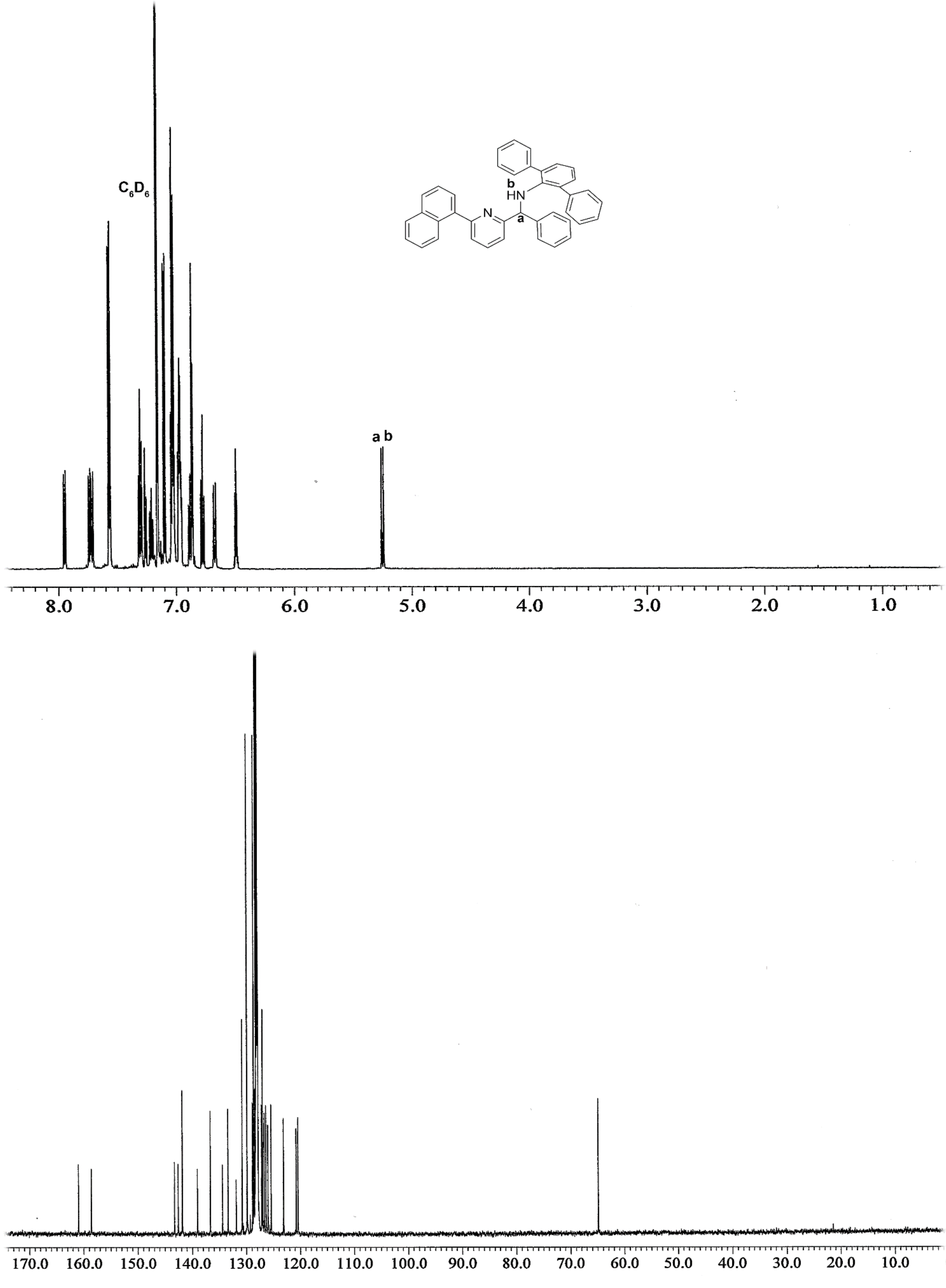

Figure S37. ^{1}H and ^{13}C NMR spectra of **33.**

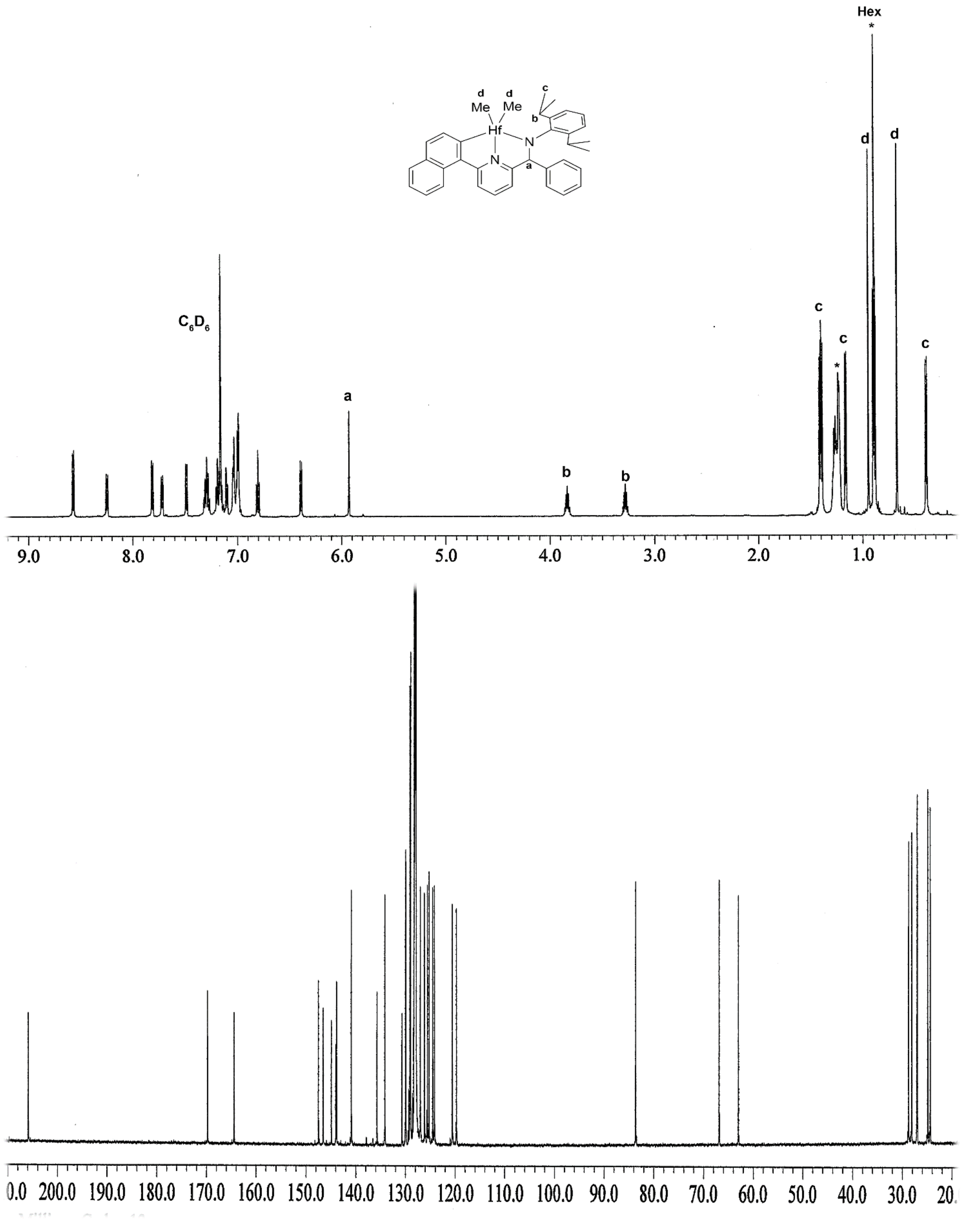

Figure 38 ^{1}H and ^{13}C NMR spectra of **34.**

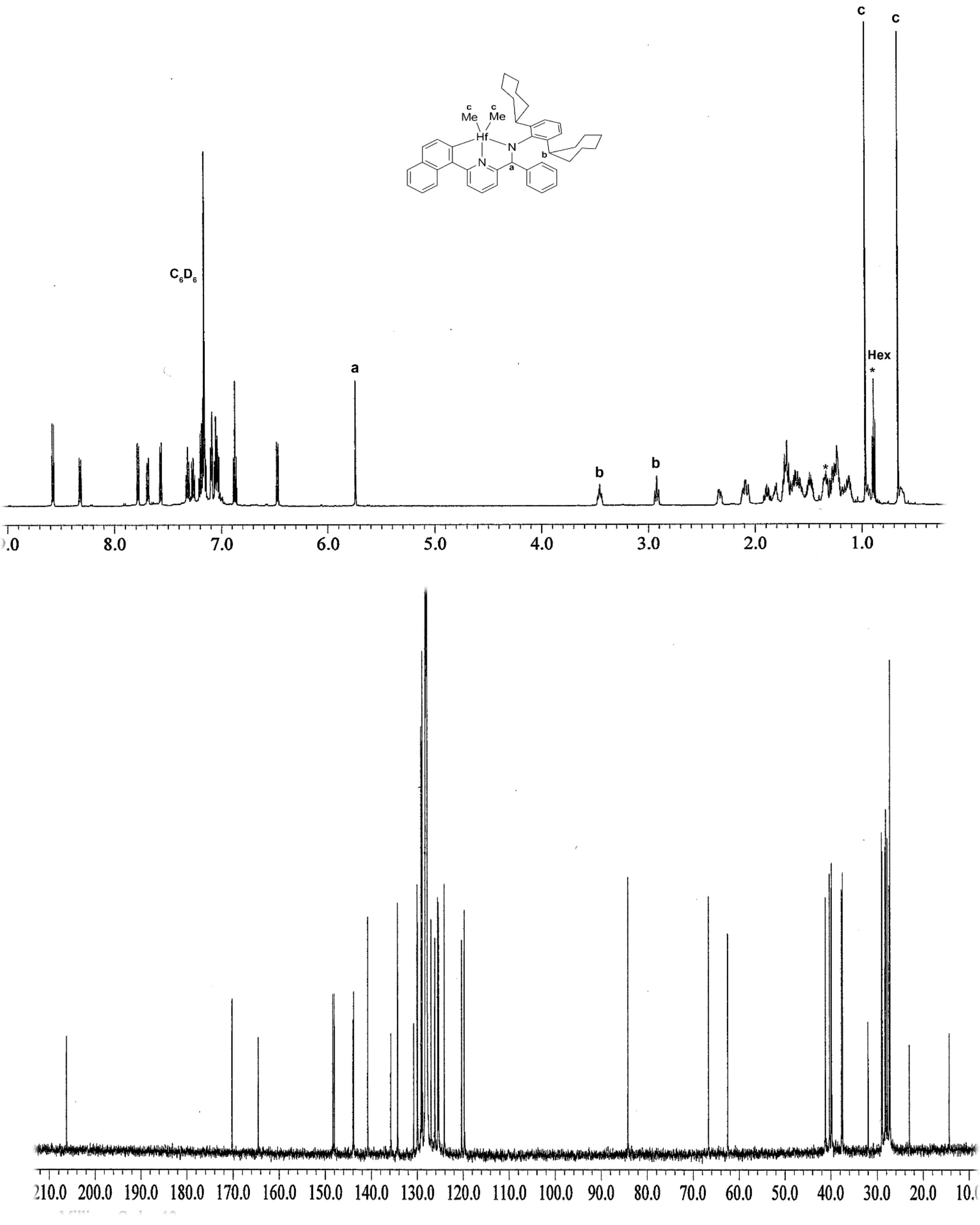

Figure S39. ^{1}H and ^{13}C NMR spectra of **35.**

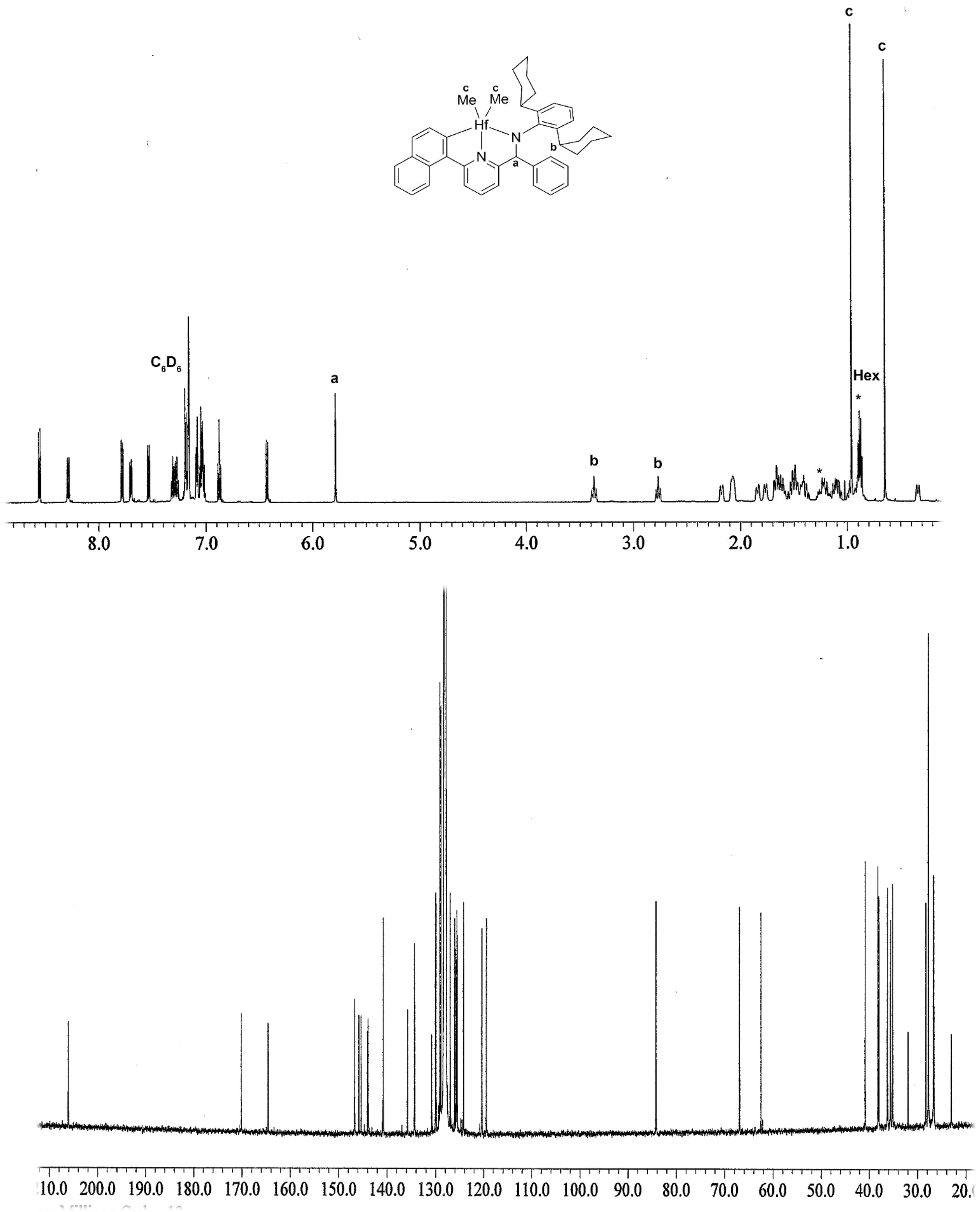

Figure S40. ^{1}H and ^{13}C NMR spectra of **36.**

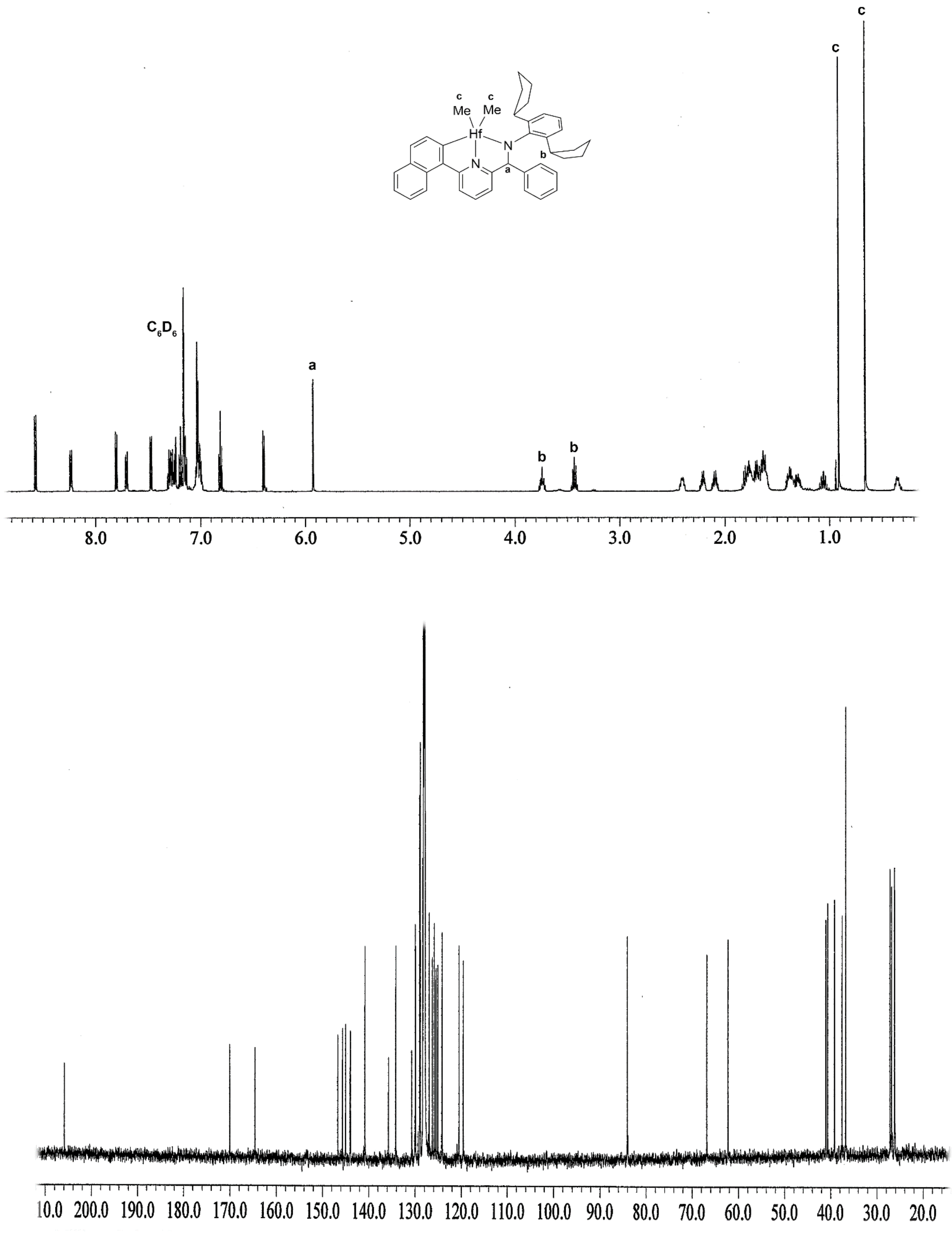

Figure S41. ^{1}H and ^{13}C NMR spectra of **37.**

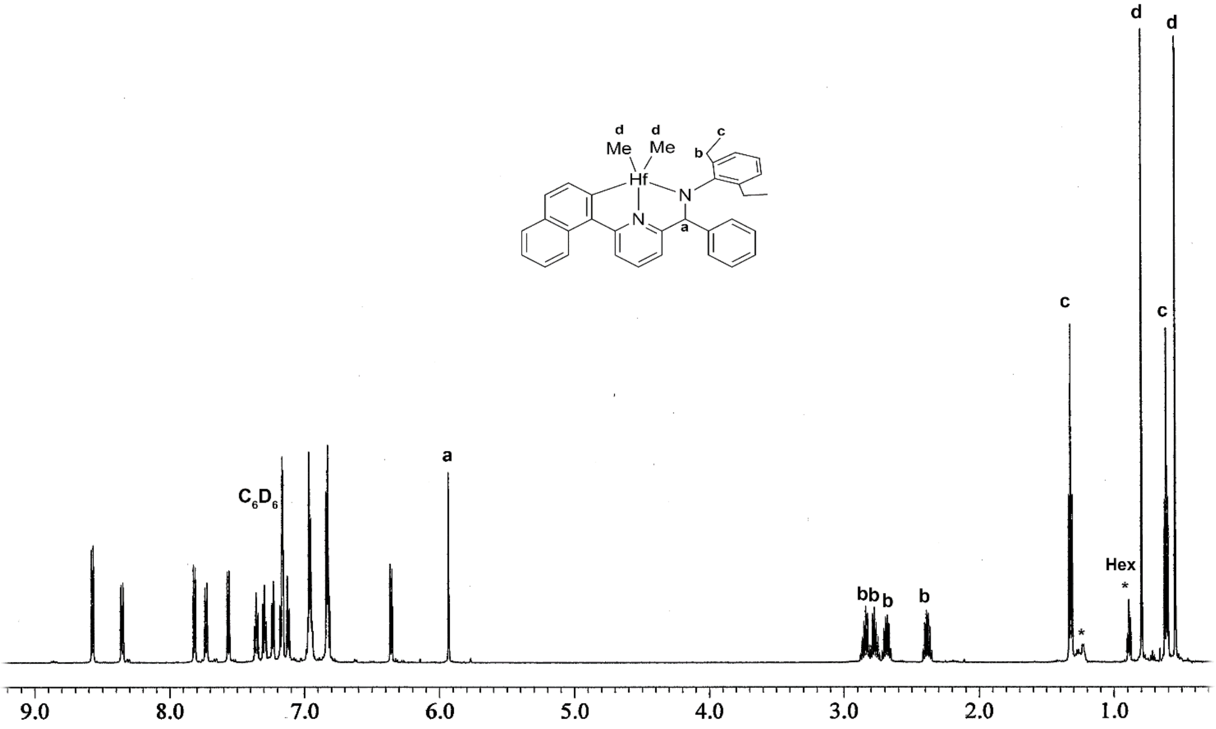
d
d
Me
Me
Hf
N
N
a
b
c
C6D6
a
bb
b
b
c
c
d
d
Hex
*
*
9.0
8.0
7.0
6.0
5.0
4.0
3.0
2.0
1.0

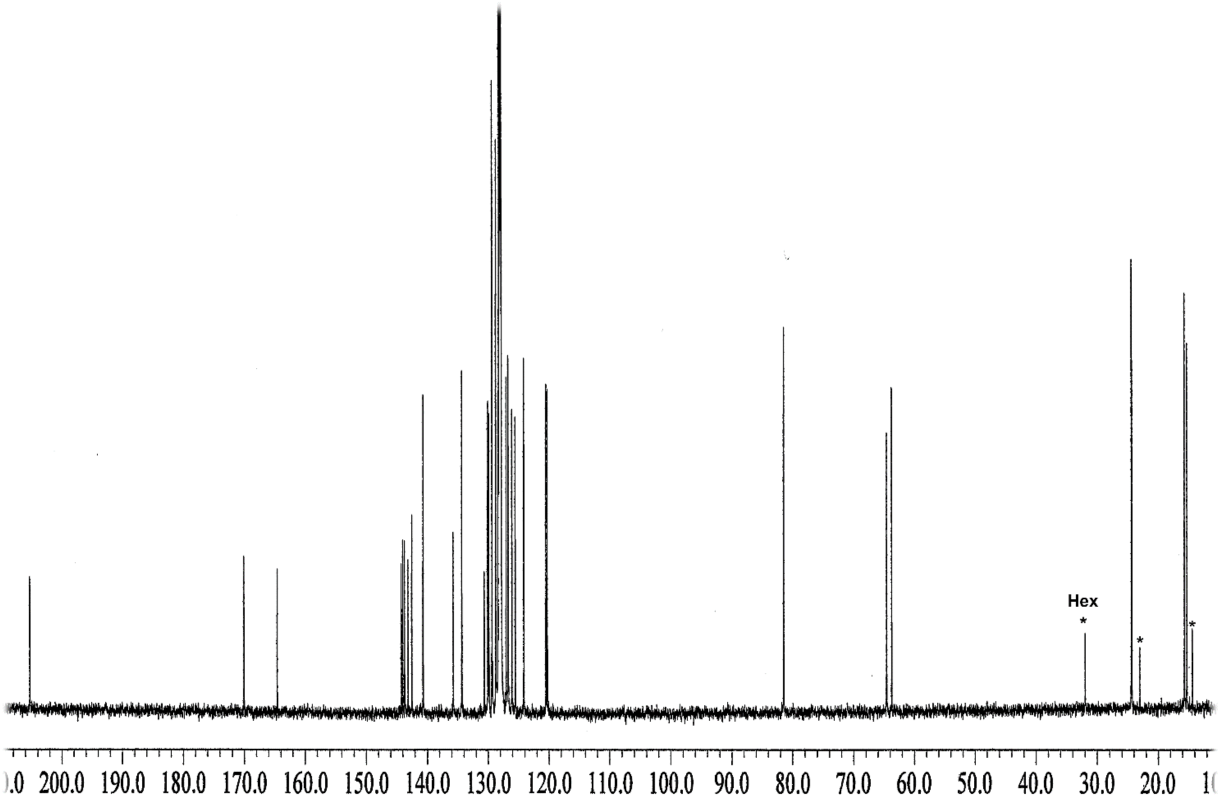
Hex
*
*
*
200.0
190.0
180.0
170.0
160.0
150.0
140.0
130.0
120.0
110.0
100.0
90.0
80.0
70.0
60.0
50.0
40.0
30.0
20.0
